# Childhood predictors of volunteering across 22 countries in the Global Flourishing Study

**DOI:** 10.1038/s41598-024-81639-w

**Published:** 2025-04-30

**Authors:** Julia S. Nakamura, Robert D. Woodberry, Cristina B. Gibson, Matthew T. Lee, Young-Il Kim, Koichiro Shiba, R. Noah Padgett, Byron R. Johnson, Tyler J. VanderWeele

**Affiliations:** 1 Department of Psychology, University of British Columbia, 2136 West Mall, V6T 1Z4 Vancouver, BC Canada; 2 Institute for Studies of Religion, Baylor University, Waco, USA; 3 Graziadio Business School, Pepperdine University, Malibu, USA; 4 Human Flourishing Program, Institute for Quantitative Social Science, Harvard University, Cambridge, USA; 5 Department of Social Work, George Fox University, Newberg, USA; 6 School of Public Health, Boston University, Boston, USA; 7 Department of Epidemiology, Harvard T.H. Chan School of Public Health, Boston, USA; 8 Department of Biostatistics, Harvard T.H. Chan School of Public Health, Boston, USA

**Keywords:** Global Flourishing Study, Volunteering, Prosocial behavior, Cross-national, Childhood, Adolescence, Epidemiology, Human behaviour

## Abstract

While prior work documents individual and societal benefits to volunteering, less is known about how childhood experiences shape volunteering in adulthood around the world. Using data from the Global Flourishing Study, a diverse and international sample of 202,898 individuals across 22 countries, we examined 11 candidate childhood predictors of volunteering in adulthood, and whether these associations varied by country. Random effects meta-analyses showed evidence of associations between several childhood predictors and an increased likelihood of subsequent volunteering, including having a good relationship with one’s father, comfortable subjective financial status, childhood abuse, feeling like an outsider growing up, more frequent religious service attendance at age 12, and being male. Sensitivity analyses showed that several of these associations were at least moderately robust to unmeasured confounding. There was little evidence of several other predictors (e.g., relationship with mother, parental marital status, self-rated health, immigration status, and year of birth) being associated with adulthood volunteering. Associations between childhood predictors and volunteering varied between countries, reflecting diverse societal influences. These findings enhance our understanding of childhood predictors of volunteering in adulthood and, with further research, provide valuable evidence for informing policy and practice aimed at fostering volunteering globally.

**Supplementary Information:**

The online version contains supplementary material available at 10.1038/s41598-024-81639-w.

## Introduction

Millions of people volunteer worldwide every day^1^. Volunteering, broadly defined as “an activity undertaken by an individual that is uncoerced, unpaid, structured by an organization, and directed toward a community concern”^2^ is common internationally^3^ and associated with improvements to societal (e.g., contributing billions to economies annually)^4–6^ and individual well-being (e.g., a reduced risk of all-cause mortality and improved physical health, health behaviors, and psychosocial well-being^7,8^). In fact, physicians and policymakers are even being encouraged to “prescribe” volunteering to their willing and able patients as a way of enhancing individual and societal health^9,10^. As we continue to see the substantial contributions of volunteering to the well-being of individuals and societies, understanding the factors that influence individuals’ volunteering engagement is of great importance for policymakers, practitioners, and researchers alike. While some progress has been made in understanding the adulthood determinants of volunteering^11,12^, relatively little attention has been paid to the role of *early-life experiences* in shaping global *adulthood* volunteering. Understanding childhood predictors of volunteering in international samples is critical for developing effective intervention strategies targeted at promoting global volunteering.

Childhood is a critical period of development during which individuals form foundational beliefs, values, and social norms that shape their attitudes and behaviors throughout life^13,14^. A large body of research has established the importance of childhood experiences in predicting adulthood behavior and well-being (e.g., associations between childhood abuse and adverse health and behavioral outcomes in adulthood^15^). Given the impact of childhood experiences on a wide array of physical and psychosocial outcomes in adulthood, research has increasingly focused on ways to limit childhood experiences associated with negative outcomes and encourage those associated with positive outcomes. Indeed, the processes by which children and adolescents become kind, considerate, concerned, and helpful toward others have been an important area of inquiry^16^. However, few studies have systematically examined the influence of childhood experiences on volunteering in adulthood, particularly in a cross-national context.

We also have a limited understanding of how childhood factors that shape volunteering differ across diverse cultures, although the extant research does indicate great variation in volunteering rates across countries^3,17^. To understand the psychological and sociocultural factors influencing prosociality, a cultural developmental approach is needed^18^. Prosocial tendencies appear early in life across various cultures, suggesting that socialization processes shape these behaviors throughout development, rather than eliciting them^18^. Indeed, differences in prosocial behaviors are already evident during childhood, though most of this research has been conducted in preschoolers and young children^16,19^.

Many childhood factors may predict adulthood volunteering. In addition to early community engagement^20–22^, a variety of other factors in childhood might predict increased adulthood volunteering and prosociality (as indicated by research on prosociality in general, and on volunteering), including: year of birth (findings are mixed, such that some studies show evidence of generational differences in volunteering rates, and others do not^23–26^), family structure (e.g., the absence of parental separation^27,28^), religious factors (e.g., increased childhood religiosity and religious service attendance^29–33^), parental factors (e.g., increased parental volunteerism^33–35^ and stronger family and parental connections^36^), and better socioeconomic status (SES) (e.g., higher childhood SES^37^).

While existing studies have made meaningful contributions to our understanding of childhood predictors of volunteering in adulthood cross-nationally, there are several limitations we aim to improve upon. First, though some prior work has assessed childhood predictors of adulthood volunteering, there is limited research on several childhood predictors of volunteering in adulthood (e.g., self-rated health, paternal relationships with children) of volunteering in adulthood. For example, although mothers are typically recognized as the primary influencers in moral socialization^16^, there is a lack of research focusing on the roles of fathers in shaping volunteer activities. Second, there are few international studies on predictors of volunteering. Most studies have been done in the United States and selected European countries^38^, failing to address non-WEIRD (western, educated, industrialized, rich, democratic)^39^ populations. Third, many studies only examine a limited number of or a single childhood predictor, preventing us from understanding how several childhood predictors compare in the same sample. Finally, most studies use university student samples, without including adults in later life stages or those without tertiary education. Our study, to our knowledge, is the first to investigate a consistently operationalized set of predictors across a diverse group of countries, based on an inclusive, multistage process of variable selection informed by experts from around the world and in several of the countries under study (see^40,41^).

In the present study, we used an exploratory, theory-generating approach to identify promising childhood predictors of volunteering, in order to inform further investigation in future research. First, we used data from a diverse and international sample of 202,898 individuals across 22 countries to evaluate 11 childhood candidate predictors (e.g., childhood religious service attendance, childhood abuse; see “Methods” section for full list) of volunteering in adulthood. We anticipated that some childhood factors would show meaningful associations with volunteering in adulthood, and we did not expect all such associations to be of equal strength. Second, we assessed if these associations varied by country. We anticipated that the strength of associations would vary by country, reflecting the influence of diverse sociocultural, economic, and health contexts that characterize each nation. Third, we anticipated that the observed associations would be robust against potential unmeasured confounding, as assessed by *E*-values.

## Methods

The description of the methods below have been adapted from VanderWeele et al. (2024)^42^. Further methodological detail is available elsewhere^40,43–48^.

### Study population

The Global Flourishing Study (GFS) is a study of 202,898 participants from 22 geographically and culturally diverse countries, with nationally representative sampling within each country, concerning the distribution of determinants of well-being. Wave 1 of the data included the following countries and territories: Argentina, Australia, Brazil, Egypt, Germany, Hong Kong, India, Indonesia, Israel, Japan, Kenya, Mexico, Nigeria, the Philippines, Poland, South Africa, Spain, Sweden, Tanzania, Turkey, United Kingdom, and the United States. The countries were selected to (a) maximize coverage of the world’s population, (b) ensure geographic, cultural, and religious diversity, and (c) prioritize feasibility and existing data collection infrastructure. Data collection was carried out by Gallup Inc. Data for Wave 1 were collected principally during 2023, with some countries beginning data collection in 2022 and exact dates varying by country^45^. Four additional waves of panel data on the participants will be collected annually from 2024 to 2027. The precise sampling design to ensure nationally representative samples varied by country and further details are available elsewhere^45^. Survey items included aspects of well-being such as happiness, health, meaning, character, relationships, and financial stability^49^, along with other demographic, social, economic, political, religious, personality, childhood, community, health, and well-being variables. The data are publicly available through the Center for Open Science (https://www.cos.io/gfs). During the translation process, Gallup adhered to the TRAPD model (translation, review, adjudication, pretesting, and documentation) for cross-cultural survey research (ccsg.isr.umich.edu/chapters/translation/overview).

### Measures

*Childhood variables*: Relationship with mother during childhood was assessed with the question: “Please think about your relationship with your mother when you were growing up. In general, would you say that relationship was very good, somewhat good, somewhat bad, or very bad?” Responses were dichotomized to very/somewhat good versus very/somewhat bad. An analogous variable was used for relationship with father. “Does not apply” was treated as a dichotomous control variable for respondents who did not have a mother or father due to death or absence. Parental marital status during childhood was assessed with responses of married, divorced, never married, and one or both had died. Subjective financial status was measured by asking participants: “Which one of these phrases comes closest to your own feelings about your family’s household income when you were growing up, such as when YOU were around 12 years old?” Responses were lived comfortably, got by, found it difficult, and found it very difficult. Childhood abuse was assessed with yes/no responses to “Were you ever physically or sexually abused when you were growing up?” To evaluate feeling like an outsider while growing up, participants were asked: “When you were growing up, did you feel like an outsider in your family?”

Childhood health was assessed with the item: “In general, how was your health when you were growing up? Was it excellent, very good, good, fair, or poor?” Immigration status was assessed by asking: “Were you born in this country, or not?” Religious attendance during childhood was assessed with the item: “How often did YOU attend religious services or worship at a temple, mosque, shrine, church, or other religious building when YOU were around 12 years old?” with responses of at least once/week, one-to-three times/month, less than once/month, or never.

Gender was assessed as male, female, or other. Continuous age (year of birth) was classified as 18–24, 25–29, 30–39, 40–49, 50–59, 60–69, 70–79, and 80 or older. Childhood religious tradition/affiliation had response categories of Christianity, Islam, Hinduism, Buddhism, Judaism, Sikhism, Baha’i, Jainism, Shinto, Taoism, Confucianism, Primal/Animist/Folk religion, Spiritism, African-Derived, some other religion, or no religion/atheist/agnostic; precise response categories varied by country^50^. When the category no religion/atheist/agnostic had more than 5% of the within country sample size, this was used as the reference category; otherwise, the most prominent religious group was used. Additionally, all religious categories endorsed by less than 3% of the within country sample size were collapsed into a single religious category. Racial/ethnic identity was assessed in some, but not all, countries, and response categories were unique to each country. For inclusion in the childhood predictor regression analyses, race/ethnic identity was collapsed into a binary variable of whether an individual was in the most prominent group versus a minority group (race plurality).

These analyses are part of a coordinated set of papers that are conducting analyses in parallel, in which we aimed to be as consistent as possible across all studies with analytic methods (so that any observed differences can be attributed to differences in constructs and not differences in analytic choices). The final set of variables was carefully selected to broadly represent potential childhood determinants of any outcome. Still, multicollinearity issues led to some conceptually distinct variables being omitted or modified (though issues may still occur for specific outcomes and countries), and thus future work may benefit from exploring specific factors more deeply. For additional details on the assessments see the COS GFS codebook or Crabtree et al.^40^.

*Outcome variable*: Volunteering was assessed with a single item, which asked, “In the past month, have you volunteered your time to an organization?” Response options were binary (yes/no).

### Statistical analysis

We estimated descriptive statistics for the observed sample, weighted to be nationally representative within country, for each childhood category. We fit a weighted modified Poisson regression model with complex survey adjusted standard errors within each country of volunteering on all of the aforementioned childhood predictor variables simultaneously. In the primary analyses, random effects meta-analyses of the regression coefficients^51,52^ along with confidence intervals, estimated proportions of effects across countries with effect sizes larger than 1.1 and smaller than 0.9, and I^2^ for evidence concerning variation within a given category across countries^53^. Forest plots of estimates are available in the online supplement.

Religious affiliation/tradition and race/ethnicity were used within country as control variables, when available, but these coefficients themselves were not included in the meta-analyses since categories/responses varied by country. All meta-analyses were conducted in **R** (R Core Team, 2024) using the metafor package^54^. Within each country, we conducted a global test of association of each childhood predictor variable group with volunteering, and we reported a pooled *p*-value^55^ across countries concerning evidence for association within any country. Bonferroni corrected *p*-value thresholds are provided based on the number of childhood variables^56,57^. For each childhood predictor, we also calculated *E*-values to evaluate the sensitivity of results to unmeasured confounding. An *E*-value is the minimum strength of the association an unmeasured confounder must have with both the outcome and the predictor, above and beyond all measured covariates, for an unmeasured confounder to explain away an association^58^. As a supplementary analysis, we conducted population weighted meta-analyses of the regression coefficients. All analyses were pre-registered with COS prior to data access, with only slight subsequent modification in the regression analyses due to multicollinearity (https://doi.org/10.17605/OSF.IO/5F46A); all code to reproduce analyses are openly available in an online repository^44^.

### Missing data

Missing data on all variables was imputed using multivariate imputation by chained equations, and five imputed datasets were used^59–62^. To account for variation in the assessment of certain variables across countries (e.g., religious affiliation/tradition and race/ethnicity), the imputation process was conducted separately in each country. This within-country imputation approach ensured that the imputation models accurately reflected country-specific contexts and assessment methods. Sampling weights were included in the imputation models to account for specific-variable missingness that may have been related to probability of inclusion in the study.

### Accounting for complex sampling design

The GFS used different sampling schemes across countries based on availability of existing panels and recruitment needs^45^. All analyses accounted for the complex survey design components by including weights, primary sampling units, and strata. Additional methodological detail, including accounting for the complex sampling design, is provided elsewhere^47^.

## Results

Table 1 provides nationally representative descriptive statistics on childhood predictors for the overall sample (all 22 countries combined) and other key sample characteristics. The sample comprised 51% females and was mostly evenly distributed across age groups (except for those aged 80+). Participants more often reported having a very good relationship with their mother (63%) and father (53%), growing up in families with married parents (75%), perceiving their family’s financial status as “getting by” (41%), not experiencing abuse (82%), and not feeling like an outsider during childhood (84%). Participants also more often rated their health as excellent (33%), identified as native to their country (94%), and attended religious services at least 1/week at age 12 (41%). 23% of participants reported volunteering. The Supplementary Material provides nationally representative descriptive statistics by country (see Supplementary Tables S1a–S22a).

**Table 1:** Nationally representative childhood descriptive statistics and other key sample characteristics.

Characteristic	*N* = 202,898^1^
Relationship with mother	
Very good	127,836 (63%)
Somewhat good	52,439 (26%)
Somewhat bad	11,060 (5.5%)
Very bad	4642 (2.3%)
Does not apply	5965 (2.9%)
Missing	956 (0.5%)
Relationship with father	
Very good	107,742 (53%)
Somewhat good	55,714 (27%)
Somewhat bad	15,807 (7.8%)
Very bad	8278 (4.1%)
Does not apply	13,985 (6.9%)
Missing	1372 (0.7%)
Parent marital status	
Yes, married	152,001 (75%)
No, divorced	17,726 (8.7%)
Never married	15,534 (7.7%)
No, one or both of them had died	7794 (3.8%)
Missing	9843 (4.9%)
Subjective financial status of family growing up	
Lived comfortably	70,861 (35%)
Got by	82,905 (41%)
Found it difficult	35,852 (18%)
Found it very difficult	12,606 (6.2%)
Missing	674 (0.3%)
Abuse	
Yes	29,139 (14%)
No	167,279 (82%)
Missing	6479 (3.2%)
Outsider growing up	
Yes	28,732 (14%)
No	170,577 (84%)
Missing	3589 (1.8%)
Self-rated health growing up	
Excellent	67,121 (33%)
Very good	63,086 (31%)
Good	47,378 (23%)
Fair	19,877 (9.8%)
Poor	4906 (2.4%)
Missing	530 (0.3%)
Immigration status	
Born in this country	190,998 (94%)
Born in another country	9791 (4.8%)
Missing	2110 (1.0%)
Age 12 religious service attendance	
At least 1/week	83,237 (41%)
1–3/month	33,308 (16%)
< 1/month	36,928 (18%)
Never	47,445 (23%)
Missing	1980 (1.0%)
Age group	
1998–2005; current age: 18–24	27,007 (13%)
1993–1998; age: 25–29	20,700 (10%)
1983–1993; age: 30–39	40,256 (20%)
1973–1983; age 40–49	34,464 (17%)
1963–1973; age 50–59	31,793 (16%)
1953–1963; age 60–69	27,763 (14%)
1943–1953; age 70–79	16,776 (8.3%)
1943 or earlier; age 80+	4119 (2.0%)
Missing	20 (< 0.1%)
Gender	
Male	98,411 (49%)
Female	103,488 (51%)
Other	602 (0.3%)
Missing	397 (0.2%)
Country	
Argentina	6724 (3.3%)
Australia	3844 (1.9%)
Brazil	13,204 (6.5%)
Egypt	4729 (2.3%)
Germany	9506 (4.7%)
India	12,765 (6.3%)
Indonesia	6992 (3.4%)
Israel	3669 (1.8%)
Japan	20,543 (10%)
Kenya	11,389 (5.6%)
Mexico	5776 (2.8%)
Nigeria	6827 (3.4%)
Philippines	5292 (2.6%)
Poland	10,389 (5.1%)
South Africa	2651 (1.3%)
Spain	6290 (3.1%)
Tanzania	9075 (4.5%)
Turkey	1473 (0.7%)
United Kingdom	5368 (2.6%)
United States	38,312 (19%)
Sweden	15,068 (7.4%)
Hong Kong	3012 (1.5%)
Volunteering	
Yes	45,914 (23%)
No	156,368 (77%)
Missing	616 (0.3%)

^1^n (%).

Table 2 shows the meta-analytic estimates of the associations between the 11 childhood candidate predictors and volunteering in adulthood. In support of the first set of relationships we anticipated, several childhood factors were associated with an increased likelihood of volunteering in adulthood. Having a very good/somewhat good vs. a very bad/somewhat bad relationship with father was associated with a 9% increased likelihood of volunteering in adulthood (RR = 1.09, 95% CI: 1.04, 1.15). Living comfortably (vs. getting by) in terms of subjective financial status of family growing up was associated with a 10% increased likelihood of volunteering (RR = 1.10, 95% CI: 1.04, 1.16). Experiencing physical or sexual abuse (RR = 1.16, 95% CI: 1.10, 1.23) and feeling like an outsider growing up (RR = 1.17, 95% CI: 1.11, 1.23) were associated with a 16% and 17% increased likelihood of volunteering, respectively.

**Table 2:** Random effects meta-analysis of regression of volunteering on childhood predictors.

Variable	Category	RR	95% CI	Estimated proportion of effects by threshold		I^2^	Global *p*-value
				< 0.90	> 1.10		
Relationship with mother	(Ref: Very bad/somewhat bad)						< 0.001**
	Very good/somewhat good	1.02	(0.95, 1.10)	0.09	0.18	38.2	
Relationship with father	(Ref: Very bad/somewhat bad)						< 0.001**
	Very good/somewhat good	1.09	(1.04, 1.15)	0.00	0.55	23.9	
Parent marital status	(Ref: Parents married)						< 0.001**
	No, divorced	0.96	(0.91, 1.02)	0.09	0.00	22.1	
	Single, never married	1.00	(0.94, 1.07)	0.09	0.18	37.2	
	No, one or both of them had died	1.02	(0.96, 1.08)	0.00	0.05	12.9	
Subjective financial status of family growing up	(Ref: Got by)						< 0.001**
	Lived comfortably	1.10	(1.04, 1.16)	0.00	0.45	73.4	
	Found it difficult	0.98	(0.95, 1.02)	0.00	0.00	< 0.1ǂ	
	Found it very difficult	1.02	(0.95,1.10)	0.14	0.27	33.3	
Abuse	(Ref: No)						< 0.001**
	Yes	1.16	(1.10, 1.23)	0.00	0.62	65.0	
Outsider growing up	(Ref: No)						< 0.001**
	Yes	1.17	(1.11, 1.23)	0.00	0.82	57.0	
Self-rated health growing up	(Ref: Good)						< 0.001**
	Excellent	1.07	(1.00, 1.15)	0.09	0.41	75.1	
	Very good	1.04	(0.98, 1.11)	0.09	0.36	68.9	
	Fair	1.02	(0.93, 1.12)	0.18	0.27	72.5	
	Poor	1.09	(0.99, 1.20)	0.00	0.41	25.3	
Immigration status	(Ref: Born in this country)						< 0.001**
	Born in another country	0.56	(0.18, 1.77)	0.41	0.32	99.7	
Age 12 religious service attendance	(Ref: Never)						< 0.001**
	At least 1/week	1.61	(1.42, 1.82)	0.00	1.00	84.3	
	1–3/month	1.48	(1.33, 1.64)	0.00	0.95	74.4	
	< 1/month	1.25	(1.15, 1.35)	0.00	0.82	56.5	
Year of birth	(Ref: 1998–2005; age 18–24)						< 0.001**
	1993–1998; age 25–29	0.98	(0.93, 1.05)	0.23	0.14	47.6	
	1983–1993; age 30–39	0.95	(0.88, 1.03)	0.36	0.09	74.8	
	1973–1983; age 40–49	1.01	(0.93, 1.09)	0.18	0.27	69.5	
	1963–1973; age 50–59	1.00	(0.92, 1.09)	0.27	0.27	70.7	
	1953–1963; age 60–69	0.97	(0.86, 1.10)	0.32	0.32	80.4	
	1943–1953; age 70–79	0.92	(0.79, 1.06)	0.55	0.14	72.2	
	1943 or earlier; age 80+^ǂǂ^	0.22	(0.04, 1.17)	0.55	0.14	99.5	
Gender	(Ref: Male)						< 0.001**
	Female	0.87	(0.80, 0.95)	0.59	0.09	92.1	
	Other^ǂǂ^	0.15	(0.02, 1.41)	0.44	0.39	99.6	

*Note. N* = 202,898. *p < 0.05; **p < 0.004 (Bonferroni corrected threshold); ^‡^estimate of heterogeneity is likely unstable, please see our online supplement forest plots for more detail on heterogeneity of effects. ^‡‡^Group is very small (< 0.1% of the observed sample) within several countries leading large uncertainty in this estimate or even complete separation—be cautious about interpreting this estimate; CI = confidence interval; the estimated proportion of effects is the estimated proportion of effects above (or below) a threshold based on the calibrated effect sizes (Mathur & VanderWeele^53^), ; *I*^2^ is an estimate of the variability in means due to heterogeneity across countries vs. sampling variability; the Global *p*-value corresponds to the joint test of the null hypothesis that the country-specific joint parameter Wald tests (all parameters within variable groups are zero) are all null all 22 countries; and additional details of heterogeneity of effects are available in the forest plots of our online supplemental material.

We also observed an association between religious service attendance and likelihood of volunteering, such that greater frequency of religious service attendance at age 12 was associated with an increased likelihood of volunteering in adulthood. For those who attended religious services < 1/month (RR = 1.25, 95% CI: 1.15, 1.35), 1–3/month (RR = 1.48, 95% CI: 1.33, 1.64), and at least 1/week (RR = 1.61, 95% CI: 1.42, 1.82), as compared to never, there was an increasing likelihood of volunteering. On the other hand, being female, as compared to being male, was associated with a 13% decreased likelihood of volunteering (RR = 0.87, 95% CI: 0.80, 0.95). There was little evidence of several other predictors, including relationship with mother, parental marital status, self-rated health, immigration status, and year of birth being associated with adulthood volunteering.

Consistent with the second set of relationships we anticipated, there was evidence of variation in differences between countries across all candidate childhood predictors as evidenced by estimates of proportion of effects by thresholds and heterogeneity (see Supplemental Figures S1–S27), possibly representing the inherent variation in culture, socioeconomic, and geographic differences across the 22 countries of interest.

Supplementary Tables S1b–S22b provide the country-specific associations between candidate childhood predictors and volunteering, and Supplementary Tables S1c–S22c contain *E*-value analyses for these associations. Supplementary Figures S1–S27 provide forest plots for candidate childhood predictors by country. In 16 countries, there was considerable evidence that attending religious services 1/week (vs. never) at age 12 was associated with an increased likelihood of volunteering, though the association was much stronger in some countries (e.g., Sweden; RR = 3.53, 95% CI: 2.90, 4.32) compared to others (e.g., Germany; RR = 1.21, 95% CI: 1.04, 1.39).

Across nine countries, there was considerable evidence that living comfortably financially growing up (vs. getting by) was associated with an increased likelihood of volunteering in adulthood, with the largest increases being in Poland (RR = 1.49, 95% CI: 1.17, 1.89) and Egypt (RR = 1.48, 95% CI: 1.04, 2.08). Notably, very difficult subjective financial status growing up (vs. getting by) had high heterogeneity and was associated with likelihood of volunteering in two countries, but in opposite directions: very difficult subjective financial status was associated with a decreased likelihood of volunteering in Nigeria (RR = 0.80, 95% CI: 0.66, 0.97), but an increased likelihood of volunteering in the United States (RR = 1.22, 95% CI: 1.01, 1.48).

In nine countries, there was notable evidence that physical or sexual abuse in childhood was associated with increased volunteering in adulthood, ranging from an 11% increased likelihood of volunteering in the United States (RR = 1.11, 95% CI: 1.01, 1.21) to a 57% increased likelihood in Japan (RR = 1.57, 95% CI: 1.31, 1.87). Likewise, there was notable evidence that feeling like an outsider growing up was similarly associated with an increased likelihood of volunteering in nine countries ranging from an 11% increased likelihood in Kenya (RR = 1.11, 95% CI: 1.01, 1.22) to a 51% increased likelihood in Japan (RR = 1.51, 95% CI: 1.27, 1.78).

There was notable evidence that being female was negatively associated with adulthood volunteering in 12 out of 22 countries; in contrast, the one country with notable evidence in the opposite direction was Poland, where being female (vs. male) was associated with a 34% increased likelihood of volunteering (RR = 1.34, 95% CI: 1.12, 1.61).

Findings for self-rated health were very mixed between countries. Experiencing excellent self-rated health growing up was associated with an increased likelihood of volunteering in adulthood in many countries, such as Argentina (RR = 1.22, 95% CI: 1.01, 1.48), Indonesia (RR = 1.23, 95% CI: 1.13, 1.36), Mexico (RR = 1.26, 95% CI: 1.05, 1.52), Hong Kong (RR = 1.55, 95% CI: 1.23, 1.96), United States (RR = 1.22, 95% CI: 1.09, 1.37) and Sweden (RR = 1.23, 95% CI: 1.02, 1.49). Notably, in some countries (e.g. India, Argentina, Mexico) better and worse (e.g., fair and/or poor) self-rated health growing up were associated with an increased likelihood of volunteering. In Israel, experiencing excellent (vs. good) self-rated health growing up was associated with a decreased likelihood of volunteering in adulthood (RR = 0.72, 95% CI: 0.57, 0.91), as was very good health (RR = 0.75, 95% CI:0.59, 0.95) and fair self-rated health (RR = 0.58, 95% CI: 0.35, 0.96).

Immigration status also showed high heterogeneity. For three countries (India, South Africa, and the United States) being an immigrant was associated with a decreased likelihood of volunteering, while in Poland (RR = 2.29, 95% CI: 1.26, 4.16), it was associated with an increased likelihood of volunteering. In Egypt, there was complete separation (i.e., we do not have information on any Egyptian respondents who are immigrants and selected volunteering), so we are unable to estimate the change in likelihood of volunteering for these individuals.

Findings across age groups were also mixed. In some countries (e.g., Israel, United Kingdom), older age groups were mostly associated with a decreased likelihood of volunteering. In other countries (e.g., Tanzania), some older age groups (e.g., 50–59, 60–69) were associated with an increased likelihood of volunteering.

Having a very good/somewhat good (vs. very bad/somewhat bad) relationship with mother was associated with a 183% increased likelihood of volunteering in Turkey (RR = 2.83, 95% CI: 1.40, 5.67), but there was no such evidence in any other countries. In contrast, there was strong evidence that having a very good/somewhat good (vs. very bad/somewhat bad) relationship with father was associated with an increased likelihood of volunteering in Argentina, India, Japan, and Mexico. Parents being divorced (vs. married) was associated with a 30% increased likelihood of volunteering in Argentina (RR = 1.30, 95% CI: 1.01, 1.67), but a decreased likelihood of volunteering in Australia (RR = 0.76, 95% CI: 0.61, 0.97) and the United States (RR = 0.82, 95% CI: 0.73, 0.92). Having a parent die was associated with an increased likelihood of volunteering in Japan (RR = 1.32, 95% CI: 1.01, 1.74), and having single, never married parents was associated with a decreased likelihood of volunteering in Sweden (RR = 0.75, 95% CI: 0.59, 0.95) and the United Kingdom (RR = 0.66, 95% CI: 0.47, 0.94). 

Childhood religious affiliation and minority race/ethnicity status were associated with adulthood volunteering in some countries. In some countries, minority status was associated with a lower likelihood of adulthood volunteering (e.g., Indonesia, United States), while in others, minority status was associated with a greater likelihood of volunteering (e.g., Kenya, Philippines). In some countries, any religious affiliation, as compared to none, was associated with an increased likelihood of volunteering (e.g., Germany, Hong Kong, Japan, Spain).

Finally, consistent with the third set of relationships anticipated, Table 3 shows *E*-values which suggested that many of the observed associations were moderately robust to unmeasured confounding. For example, for childhood abuse, an unmeasured confounder that was associated with both childhood abuse and volunteering by risk ratios of 1.59 each (above and beyond the covariates already adjusted for) could explain away the association, but weaker joint confounder associations could not. Further, to shift the confidence interval to include the null, an unmeasured confounder associated with both childhood abuse and volunteering by risk ratios of 1.42 each could suffice, but weaker joint confounder associations could not. Results for meta-analyses using a population weighted approach and E-values for these analyses are presented in Supplementary Tables S23-S24, respectively.

**Table 3:** Sensitivity of meta-analyzed childhood predictors to unmeasured confounding.

Variable	Category	E-value for estimate	E-value for 95% CI
Relationship with mother	(Ref: Very bad/somewhat bad)		
	Very good/somewhat good	1.17	1.00
Relationship with father	(Ref: Very bad/somewhat bad)		
	Very good/somewhat good	1.41	1.24
Parent marital status	(Ref: Parents married)		
	No, divorced	1.23	1.00
	Single, never married	1.05	1.00
	No, one or both of them had died	1.14	1.00
Subjective financial status of family growing up	(Ref: Got by)		
	Lived comfortably	1.42	1.24
	Found it difficult	1.16	1.00
	Found it very difficult	1.17	1.00
Abuse	(Ref: No)		
	Yes	1.59	1.42
Outsider growing up	(Ref: No)		
	Yes	1.61	1.45
Self-rated health growing up	(Ref: Good)		
	Excellent	1.34	1.00
	Very good	1.25	1.00
	Fair	1.18	1.00
	Poor	1.40	1.00
Immigration status	(Ref: Born in this country)		
	Born in another country	2.95	1.00
Age 12 religious service attendance	(Ref: Never)		
	At least 1/week	2.59	2.20
	1–3/month	2.32	2.00
	< 1/month	1.80	1.57
Year of birth	(Ref: 1998–2005; age 18–24)		
	1993–1998; age 25–29	1.14	1.00
	1983–1993; age 30–39	1.27	1.00
	1973–1983; age 40–49	1.09	1.00
	1963–1973; age 50–59	1.02	1.00
	1953–1963; age 60–69	1.21	1.00
	1943–1953; age 70–79	1.41	1.00
	1943 or earlier; age 80+	8.40	1.00
Gender	(Ref: Male)		
	Female	1.56	1.30
	Other	13.06	1.00

## Discussion

Using data from an international sample of 202,898 individuals across 22 countries, we assessed the associations between 11 childhood candidate predictors and volunteering in adulthood. As expected, several childhood factors were associated with an increased likelihood of volunteering in adulthood. Our findings converged with prior research, such that higher SES (living comfortably [vs. getting by] for subjective family financial status)^37^, and more frequent religious service attendance^32^ were associated with increased volunteering. Overall, there were a number of small effect sizes (though many are comparable to those found in prior research on adulthood predictors of volunteering)^12^ and some surprising findings (e.g., experiencing childhood abuse being associated with more volunteering). Potentially, some findings may be due to limitations associated with methodological constraints, such as self-report/retrospective bias, and many of these findings point to the need for more rigorous designs to evaluate these associations in future research. Thus, many potential explanations offered below are speculative.

This convergence with previous research applies to the analysis of the 22-country sample overall, but as expected, we found more variation when we compared results across countries, consistent with the second set of relationships we anticipated. Future research is needed to understand why such disparate patterns emerged, such as the positive association of religious service attendance at least 1/week vs. never on volunteering in 16 countries but not in the other six. It is possible that religious organizations in some countries encourage the direct expression of prosocial activities to support members of the community or family members, instead of encouraging attendees to volunteer for other organizations. If this is the case, then the overall amount of prosocial activity in these countries might not be diminished, even if the social distribution of volunteering is quite different.

Our data do not speak directly to this issue, but we point to cross-national data on one form of volunteering, blood donation, as an example of how volunteering might be better understood not only as a purposive individual choice, but also an outcome of social structures. Comparing patterns of blood donation in European Union countries, for example, suggests that “blood can be seen not so much as something that individuals donate, but as something that organizations collect”^63^ (p. 1634). Some countries rely on a large number of people who give blood infrequently, while others get most of their blood supply from a small number of repeat donors. If blood donation was a “pure” example of individual altruism, we would not find these striking differences across collection regimes. But the differences that do exist may be explained by structural variables. For example, countries that rely on the Red Cross to collect blood may tap into a small pool of regular donors who are recruited from networks of volunteers. These donors tend to be involved in churches and active in a number of voluntary organizations. The act of donating blood is seen as consistent with these altruistic activities. Conversely, countries that rely on state-run agencies to collect blood may recruit more broadly from the population, but donors may be less likely to give repeatedly. Similar dynamics might help explain the cross-national variations that we observed, but confirmation of this hypothesis awaits further research.

Regardless of country-specific nuances, we found that increasing frequency of religious service attendance at age 12 was the strongest predictor of adulthood volunteering (RR = 1.61, 95% CI: 1.42, 1.82, *E*-value = 2.59). This association was observed for all frequencies of attendance, as compared to never attending religious services, although more frequent attendance was associated with higher increases. This is consistent with previous literature that points to a strong positive association between childhood religiosity and religious service attendance and volunteering in adulthood^29,30^, even when adult religious involvement is accounted for^31^, and has emerged as an important predictor above and beyond other sociodemographic factors in prior research (e.g., gender, marital status, or country of birth^32^).

There are many possible factors that could contribute to this strong association, including: (a) increased access to more volunteer work through increased social and religious networks^64,65^, (b) cultural norms and values imparted by religion, (c) continuation of religious beliefs and practices into adulthood, and (d) modeling—in which children who viewed their religious parents acting in prosocial ways or directly volunteering may come to learn to do it themselves as well^16^—amongst other potential explanations. Associations between childhood religious service attendance and adulthood volunteering differed between countries: in all countries, any (as compared to no) religious service attendance in childhood was associated with an increased (not decreased in any countries) likelihood of volunteering in adulthood, but with differences in magnitude. For attending at least 1/week, estimates ranged from a 253% increased likelihood of volunteering in Sweden, a 197% increased likelihood in Japan, and a 108% increased likelihood in Israel, to a 21% increased likelihood in Germany.

Other aspects of childhood environments may promote volunteering in adulthood, including financial status, relationships with parents, and gender. Living comfortably (vs. getting by) in terms of subjective financial status of family, was associated with an increased likelihood of volunteering in the overall sample and in several individual countries. Indeed, in prior research, fewer financial resources in childhood has been associated with less volunteering and civic engagement in adulthood^37^. Greater monetary resources during childhood may translate to greater monetary resources in adulthood and the ability to devote time to volunteer work. Notably, in only the United States, very difficult subjective financial status of family (vs. getting by) was associated with a greater likelihood of volunteering in adulthood. It is possible that lower SES and working-class families may have more holistic thinking and interdependent views as compared to other Americans – that is, thinking of oneself as not separate from the whole may prompt people to attempt to benefit the whole (e.g., through volunteering), especially if it is within their in-group^66^. However, in Nigeria, very difficult subjective financial status of family (vs. getting by) was associated with a decreased likelihood of volunteering in adulthood – potentially pointing to differences in the role of subjective financial status of family in developing vs. developed countries.

In many countries, excellent self-rated health in childhood was associated with an increased likelihood of volunteering in adulthood. A foundation of adequate physical health in childhood may help foster the development of key cognitive, emotional, and social skills that then facilitates volunteer work in adulthood. It is also plausible that excellent childhood health enables participation in athletic teams, many of which participate in volunteering activities, which may foster the same behavior in adulthood. However, excellent self-rated health does not appear to be necessary for adulthood volunteering, as evidenced by several countries in which fair/poor self-rated health was associated with increased volunteering.

Additionally, having a good relationship with one’s father was associated with an increased likelihood of volunteering in the overall sample, and in four countries: Argentina, India, Japan, and Mexico. A good relationship with one’s father might foster a sense of security and greater emotional/financial resource availability, all of which may encourage subsequent volunteerism. Further, if fathers in these countries are themselves more likely to volunteer (which may be possible based on gender differences in volunteering^41^), their children might be more inclined to adopt similar behaviors through modeling. Children may learn prosocial behaviors through observation, imitation, and reinforcement from their parents^18,16^, especially when parental relationships are good. Indeed, children and adolescents demonstrate a greater tendency to volunteer if their parents also do so^33–35^ and these effects seem to endure into adulthood. Engagement with a parent in community groups like scouts^21,22^ or 4-H^67^ and volunteering together in the community has been associated with adulthood volunteering and community engagement,^33^ and stronger family connections may be associated with more volunteering^36^. These countries may also have cultural norms regarding family roles and community involvement which may influence how good paternal relationships impact volunteering. Notably, having a good relationship with one’s mother was not associated with an increased likelihood of volunteering in the overall sample, or in any countries besides Turkey, which aligns with prior research which found no direct effect between maternal intimacy and adolescent volunteering^68^. However, some of this may be due to these variables being highly correlated and potentially colinear in several countries.

Finally, being female was associated with a decreased likelihood of volunteering in the overall sample and most countries (but not Poland, where being female was associated with an increased likelihood of volunteering). As described elsewhere^41^, males often have more social capital (e.g., trust and social networks) and resources that facilitate volunteering and females may do more “invisible work” in their homes (e.g., cleaning, taking care of children) that may make them less available to volunteer.

While there was evidence amongst several candidate childhood predictors that more healthy childhood environments (e.g., more comfortable financial status, good relationships with parents) were associated with increased volunteering, there was also evidence that some adverse childhood factors (namely, experiencing physical or sexual abuse and feeling like an outsider) were associated with increased volunteering. These findings were somewhat unexpected, as a large body of prior research shows that adverse childhood experiences may be associated with adverse health and behavioral outcomes in adulthood (e.g., drug abuse, smoking, physical inactivity^15^).

There are a few possible explanations for this finding. First, those who have experienced childhood abuse may have increased empathy and compassion, and/or the need for a sense of purpose and meaning from the adversities, which may promote prosociality^69^. Indeed, childhood trauma has been associated with more civic engagement (as a volunteer or for pay^70^), potentially because some who have experienced this kind of trauma are often motivated to help others (altruism born of suffering^69,71,72^). An emerging body of research explains such *posttraumatic growth*^73^, part of a more general category of *growth through loss and adversity*^74^, as being the result of the emergence of “wisdom,” an “appreciation of paradox”^74^ (p. 10), “insight into the meaning of life and the importance of relationships”^74^ (p. 7). Such expressions of growth in transcending crisis are inspiring to others—many great historical leaders experienced trauma or loss as a turning point in their lives—and they occur among people who experience a variety of forms of adversity, from physical disability and serious health conditions, to grief over the death or trauma of a loved one, as well as the direct experience of trauma^75^. How and why specific countries in our sample encourage such growth among those who experienced abuse as a child or who felt like an outsider during childhood requires further research.

A second explanation is that engaging in volunteering may serve as an alternative means for children who experienced childhood adversity to establish positive roles, relationships, and social connection^76^. Notably, these associations differ between countries: in many countries, physical or sexual abuse in childhood was associated with an increased likelihood of volunteering in adulthood (feeling like an outsider was likewise associated with an increased of volunteering in several countries), but these associations varied meaningfully in magnitude (see “Results”). This may be due to cultural differences in social acceptability and norms of disclosing abuse. For example, in Japan, experiencing abuse was associated with a 57% increased likelihood of volunteering. Japanese culture emphasizes keeping face among the community and may be more inclined to shame^77^. This may discourage people in Japan from seeking out support for their abuse, and instead looking for social connection outside of one’s family, such as through volunteering. It is possible that people in other countries where this association was weaker (e.g., the United States) are more likely to derive the social support they need from social networks and supports other than volunteering. Notably, there are many potential other explanations which will need to be explored with more rigorous designs in future research.

Our study has several limitations. First, the childhood predictors were reported retrospectively, and thus may be subject to recall bias, which is a serious potential limitation. However, for recall bias to completely explain away the observed associations would require that the effect of volunteering on biasing retrospective assessments of the childhood predictors would essentially have to be at least as strong as the observed associations themselves^78^, and several of these were quite substantial. Second, we did not assess all possible candidate predictors – many other childhood factors may predict adulthood volunteering (e.g., volunteering itself in childhood and adolescence has been repeatedly associated with adulthood volunteering, social capital, and community engagement^20–22^). Third, we measured monthly prevalence of volunteering (i.e., yes or no), which is admittedly crude and does not capture other important aspects of volunteering (e.g., frequency, scope, duration, regularity). Future work should assess these aspects of volunteering to better understand how childhood predictors are associated with adulthood volunteering. Fourth, there is always potential for confounding by unmeasured variables in observational research. However, we controlled for a range of potential confounding variables, and *E*-value analyses suggested that many of the observed associations were moderately robust to unmeasured confounding, consistent with the third set of relationships we anticipated. Fifth, other contextual factors must be considered when assessing associations between childhood factors and volunteering in adulthood. With data collection in 2022 and 2023, it is possible that many volunteering opportunities were in the process of being restarted or reshaped in post COVID-19 contexts, potentially altering the availability and nature of volunteering opportunities during the study period. Wave 2 of the GFS (data collection currently underway) will help assess childhood predictors of volunteering in post-pandemic contexts. Further, availability of volunteer opportunities may vary meaningfully between countries. This variation may influence the cross-national variation we observed in the associations between childhood predictors and adulthood volunteering. We analyze possible reasons for this cross-national variation in volunteering elsewhere^41^. This study also had many strengths: it was among the first studies to evaluate childhood predictors of volunteering in adulthood cross-nationally, using large, diverse, and nationally representative samples from 22 countries.

Our findings highlight the potential impact of early-life experiences, personal attributes, and familial or social circumstances that may shape adulthood volunteering. Both positive aspects of childhood environments (e.g., comfortable subjective financial status, good relationship with father, etc.) and negative aspects of childhood (e.g., physical or sexual abuse, feeling like an outsider) were associated with an increased likelihood of volunteering in adulthood, and there was meaningful variation between countries in the magnitude and direction of associations. With further research, these findings may inform the development of programs and policies designed to foster volunteering, and in-turn promote societal and individual well-being, around the world.

## Electronic supplementary material

Below is the link to the electronic supplementary material.

Supplementary Material 1

## Data Availability

Data for Wave 1 of the Global Flourishing Study is available through the Center for Open Science upon submission of a pre-registration (https://doi.org/10.17605/OSF.IO/3JTZ8). Please see https://www.cos.io/gfs-access-data for more information about data access. All analyses were pre-registered with COS prior to data access (https://doi.org/10.17605/OSF.IO/5F46A); all code to reproduce analyses are openly available in an online repository (https://doi.org/10.17605/OSF.IO/VBYPE).
