# Supplementary material for: Childhood predictors of volunteering across 22 countries in the Global Flourishing Study
Source: Sci Rep. 2025 Apr 30;15:14797. doi: 10.1038/s41598-024-81639-w (PMC12043808; doi:10.1038/s41598-024-81639-w)

**Supplementary Material**

***Childhood Predictors of Volunteering: A Cross-National Analysis in the Global Flourishing Study***

This online supplement to the Global Flourishing Study paper on volunteering has several important caveats to interpretation. First, estimating the within country group means can be unstable if the group size is small (<1%) of the country sample size. In such cases, the uncertainty in the estimate leads to a multiple imputation adjusted degrees of freedom less than 1. This means there is not enough information to evaluate the uncertainty in the estimate. We flagged such cases with a “*”. Secondly, in rare instances for childhood predictor analyses the confidence interval of the effect estimate can contradict the reported global p-value (e.g., for the single-category effects of relationship with mother). In such cases, the reported confidence interval is more robust with corrected degrees of freedom from the pooling across multiple imputations, whereas the global p-value is based on a Wald-type test and is less robust to uncertainty attributable to multiple imputation. Third, comparing results across countries should be done with caution due to possible measurement non-invariance and differences in translation.

**Supplementary Tables**

**Table S1a.** Nationally representative descriptive statistics for Argentina

**Table S1b.** Regression of volunteering on childhood predictors for Argentina

**Table S1c.** Sensitivity to unmeasured confounding of childhood predictors in Argentina

**Table S2a.** Nationally representative descriptive statistics for Australia

**Table S2b.** Regression of volunteering on childhood predictors for Australia

**Table S2c.** Sensitivity to unmeasured confounding of childhood predictors in Australia

**Table S3a.** Nationally representative descriptive statistics for Brazil

**Table S3b.** Regression of volunteering on childhood predictors for Brazil

**Table S3c.** Sensitivity to unmeasured confounding of childhood predictors in Brazil

**Table S4a.** Nationally representative descriptive statistics for Egypt

**Table S4b.** Regression of volunteering on childhood predictors for Egypt

**Table S4c.** Sensitivity to unmeasured confounding of childhood predictors in Egypt

**Table S5a.** Nationally representative descriptive statistics for Germany

**Table S5b.** Regression of volunteering on childhood predictors for Germany

**Table S5c.** Sensitivity to unmeasured confounding of childhood predictors in Germany

**Table S6a.** Nationally representative descriptive statistics for Hong Kong

**Table S6b.** Regression of volunteering on childhood predictors for Hong Kong

**Table S6c.** Sensitivity to unmeasured confounding of childhood predictors in Hong Kong

**Table S7a.** Nationally representative descriptive statistics for India

**Table S7b.** Regression of volunteering on childhood predictors for India

**Table S7c.** Sensitivity to unmeasured confounding of childhood predictors in India

**Table S8a.** Nationally representative descriptive statistics for Indonesia

**Table S8b.** Regression of volunteering on childhood predictors for Indonesia

**Table S8c.** Sensitivity to unmeasured confounding of childhood predictors in Indonesia

**Table S9a.** Nationally representative descriptive statistics for Israel

**Table S9b.** Regression of volunteering on childhood predictors for Israel

**Table S9c.** Sensitivity to unmeasured confounding of childhood predictors in Israel

**Table S10a.** Nationally representative descriptive statistics for Japan

**Table S10b.** Regression of volunteering on childhood predictors for Japan

**Table S10c.** Sensitivity to unmeasured confounding of childhood predictors in Japan

**Table S11a.** Nationally representative descriptive statistics for Kenya

**Table S11b.** Regression of volunteering on childhood predictors for Kenya

**Table S11c.** Sensitivity to unmeasured confounding of childhood predictors in Kenya

**Table S12a.** Nationally representative descriptive statistics for Mexico

**Table S12b.** Regression of volunteering on childhood predictors for Mexico

**Table S12c.** Sensitivity to unmeasured confounding of childhood predictors in Mexico

**Table S13a.** Nationally representative descriptive statistics for Nigeria

**Table S13b.** Regression of volunteering on childhood predictors for Nigeria

**Table S13c.** Sensitivity to unmeasured confounding of childhood predictors in Nigeria

**Table S14a.** Nationally representative descriptive statistics for Philippines

**Table S14b.** Regression of volunteering on childhood predictors for Philippines

**Table S14c.** Sensitivity to unmeasured confounding of childhood predictors in Philippines

**Table S15a.** Nationally representative descriptive statistics for Poland

**Table S15b.** Regression of volunteering on childhood predictors for Poland

**Table S15c.** Sensitivity to unmeasured confounding of childhood predictors in Poland

**Table S16a.** Nationally representative descriptive statistics for South Africa

**Table S16b.** Regression of volunteering on childhood predictors for South Africa

**Table S16c.** Sensitivity to unmeasured confounding of childhood predictors in South Africa

**Table S17a.** Nationally representative descriptive statistics for Spain

**Table S17b.** Regression of volunteering on childhood predictors for Spain

**Table S17c.** Sensitivity to unmeasured confounding of childhood predictors in Spain

**Table S18a.** Nationally representative descriptive statistics for Sweden

**Table S18b.** Regression of volunteering on childhood predictors for Sweden

**Table S18c.** Sensitivity to unmeasured confounding of childhood predictors in Sweden

**Table S19a.** Nationally representative descriptive statistics for Tanzania

**Table S19b.** Regression of volunteering on childhood predictors for Tanzania

**Table S19c.** Sensitivity to unmeasured confounding of childhood predictors in Tanzania

**Table S20a.** Nationally representative descriptive statistics for Turkey

**Table S20b.** Regression of volunteering on childhood predictors for Turkey

**Table S20c.** Sensitivity to unmeasured confounding of childhood predictors in Turkey

**Table S21a.** Nationally representative descriptive statistics for United Kingdom

**Table S21b.** Regression of volunteering on childhood predictors for United Kingdom

**Table S21c.** Sensitivity to unmeasured confounding of childhood predictors in United Kingdom

**Table S22a.** Nationally representative descriptive statistics for United States

**Table S22b.** Regression of volunteering on childhood predictors for United States

**Table S22c.** Sensitivity to unmeasured confounding of childhood predictors in United States

**Table S23.** Population weighted meta-analysis of regression results

**Table S24.** Population weighted meta-analysis of E-values

**Supplementary Figures**

**Figure S1.** Forest plot for ‘Relationship with mother’ – ‘Very good/somewhat good’ effect

**Figure S2.** Forest plot for ‘Relationship with father’ – ‘Very good/somewhat good’ effect

**Figure S3.** Forest plot for ‘Parent marital status’ – ‘No, divorced’ effect

**Figure S4.** Forest plot for ‘Parent marital status’ – ‘Single, never married’ effect

**Figure S5.** Forest plot for ‘Parent marital status’ – ‘No, one or both of them has died’ effect

**Figure S6.** Forest plot for ‘Subjective financial status of family growing up’ – ‘Lived comfortably’ effect

**Figure S7.** Forest plot for ‘Subjective financial status of family growing up’ – ‘Found it difficult’ effect

**Figure S8.** Forest plot for ‘Subjective financial status of family growing up’ – ‘Found it very difficult’ effect

**Figure S9.** Forest plot for ‘Abuse’ – ‘Yes’ effect

**Figure S10.** Forest plot for ‘Outsider growing up’ – ‘Yes’ effect

**Figure S11.** Forest plot for ‘Self-rated health growing up’ – ‘Excellent’ effect

**Figure S12.** Forest plot for ‘Self-rated health growing up’ – ‘Very good effect

**Figure S13.** Forest plot for ‘Self-rated health growing up’ – ‘Fair’ effect

**Figure S14.** Forest plot for ‘Self-rated health growing up’ – ‘Poor’ effect

**Figure S15.** Forest plot for ‘Immigration status’ – ‘Born in another country’ effect

**Figure S16.** Forest plot for ‘Age 12 religious service attendance’ – ‘At least 1/week’ effect

**Figure S17.** Forest plot for ‘Age 12 religious service attendance’ – ‘1-3/month’ effect

**Figure S18.** Forest plot for ‘Age 12 religious service attendance’ – ‘<1/month’ effect

**Figure S19.** Forest plot for ‘Year of birth’ – ‘1993-1998; age 25-29’ effect

**Figure S20.** Forest plot for ‘Year of birth’ – ‘1983- 1993; age 30-39’ effect

**Figure S21.** Forest plot for ‘Year of birth’ – ‘1973-1983; age 40-49’ effect

**Figure S22.** Forest plot for ‘Year of birth’ – ‘1963-1973; age 50-59’ effect

**Figure S23.** Forest plot for ‘Year of birth’ – ‘1953-1963; age 60-69’ effect

**Figure S24.** Forest plot for ‘Year of birth’ – ‘1943 or earlier; age 80+’ effect

**Figure S25.** Forest plot for ‘Year of birth’ – ‘1943-1953; age 70-79’ effect

**Figure S26.** Forest plot for ‘Gender’ – ‘Female’ effect

**Figure S27.** Forest plot for ‘Gender’ – ‘Other’ effect

***Table S1a. Nationally representative descriptive statistics for Argentina***

| **Characteristic** | **N = 6,724**^1^ |
| --- | --- |
| **Relationship with mother** |  |
| Very good | 4,463 (66%) |
| Somewhat good | 1,436 (21%) |
| Somewhat bad | 299 (4.4%) |
| Very bad | 216 (3.2%) |
| Does not apply | 273 (4.1%) |
| Missing | 36 (0.5%) |
| **Relationship with father** |  |
| Very good | 3,612 (54%) |
| Somewhat good | 1,537 (23%) |
| Somewhat bad | 440 (6.5%) |
| Very bad | 401 (6.0%) |
| Does not apply | 694 (10%) |
| Missing | 39 (0.6%) |
| **Parent marital status** |  |
| Yes, married | 4,110 (61%) |
| No, divorced | 637 (9.5%) |
| Never married | 1,368 (20%) |
| No, one or both of them had died | 199 (3.0%) |
| Missing | 410 (6.1%) |
| **Subjective financial status of family growing up** |  |
| Lived comfortably | 2,042 (30%) |
| Got by | 2,305 (34%) |
| Found it difficult | 1,789 (27%) |
| Found it very difficult | 569 (8.5%) |
| Missing | 19 (0.3%) |
| **Abuse** |  |
| Yes | 1,302 (19%) |
| No | 5,271 (78%) |
| Missing | 151 (2.2%) |
| **Outsider growing up** |  |
| Yes | 1,165 (17%) |
| No | 5,458 (81%) |
| Missing | 101 (1.5%) |
| **Self-rated health growing up** |  |
| Excellent | 2,402 (36%) |
| Very good | 1,819 (27%) |
| Good | 1,830 (27%) |
| Fair | 505 (7.5%) |
| Poor | 156 (2.3%) |
| Missing | 12 (0.2%) |
| **Immigration status** |  |
| Born in this country | 6,346 (94%) |
| Born in another country | 348 (5.2%) |
| Missing | 29 (0.4%) |
| **Age 12 religious service attendance** |  |
| At least 1/week | 2,601 (39%) |
| 1-3/month | 1,204 (18%) |
| <1/month | 1,059 (16%) |
| Never | 1,808 (27%) |
| Missing | 53 (0.8%) |
| **Age group** |  |
| 1998-2005; current age: 18-24 | 1,108 (16%) |
| 1993-1998; age: 25-29 | 719 (11%) |
| 1983-1993; age: 30-39 | 1,432 (21%) |
| 1973-1983; age 40-49 | 1,254 (19%) |
| 1963-1973; age 50-59 | 1,014 (15%) |
| 1953-1963; age 60-69 | 730 (11%) |
| 1943-1953; age 70-79 | 356 (5.3%) |
| 1943 or earlier; age 80+ | 112 (1.7%) |
| Missing | 0 (0%) |
| **Gender** |  |
| Male | 3,143 (47%) |
| Female | 3,542 (53%) |
| Other | 21 (0.3%) |
| Missing | 18 (0.3%) |
| **Religious affiliation at age 12** |  |
| Buddhism | 3 (<0.1%) |
| Christianity | 5,805 (86%) |
| Hinduism | 2 (<0.1%) |
| Islam | 11 (0.2%) |
| Judaism | 51 (0.8%) |
| No religion/Atheist/Agnostic | 697 (10%) |
| Primal, Animist, or Folk religion | 17 (0.2%) |
| Sikhism | 5 (<0.1%) |
| Some other religion | 10 (0.2%) |
| Taoism | 1 (<0.1%) |
| Missing | 122 (1.8%) |
| **Race and ethnicity** |  |
| Asian | 43 (0.6%) |
| Black | 95 (1.4%) |
| Indigenous | 129 (1.9%) |
| Mestizo(a) | 1,801 (27%) |
| Mullato(a) | 75 (1.1%) |
| Other | 104 (1.5%) |
| White | 3,406 (51%) |
| Missing | 1,070 (16%) |
| ^1^n (%) | |

***Table S1b. Regression of volunteering on childhood predictors for Argentina***

| Variable | Category | Risk ratio | 95% CI | Global p-value |
| --- | --- | --- | --- | --- |
| Relationship with mother (Ref: Very bad/Somewhat bad) | Very good/Somewhat good | 0.97 | (0.74, 1.26) | 0.933 |
| Relationship with father (Ref: Very bad/Somewhat bad) | Very good/Somewhat good | 1.26 | (1.01, 1.58) | <.001 |
| Parent marital status (Ref: Parents married) | No, divorced | 1.30 | (1.01, 1.67) | 0.01 |
|  | Never | 1.13 | (0.93, 1.36) |  |
|  | No, one or both of them had died | 0.99 | (0.66, 1.50) |  |
| Subjective financial status of family growing up (Ref: Got by) | Lived comfortably | 1.23 | (1.05, 1.47) | 0.001 |
|  | Found it difficult | 1.14 | (0.95, 1.36) |  |
|  | Found it very difficult | 1.32 | (1.00, 1.74) |  |
| Abuse (Ref: No) | Yes | 1.19 | (1.00, 1.40) | 0.001 |
| Outsider growing up (Ref: No) | Yes | 1.09 | (0.91, 1.32) | 0.314 |
| Self-rated health growing up (Ref: Good) | Excellent | 1.22 | (1.01, 1.48) | 0.001 |
|  | Very good | 1.20 | (0.98, 1.47) |  |
|  | Fair | 1.46 | (1.12, 1.91) |  |
|  | Poor | 1.36 | (0.83, 2.25) |  |
| Immigration status (Ref: Born in this country) | Born in another country | 1.03 | (0.76, 1.40) | 0.998 |
| Age 12 religious service attendance (Ref: Never) | At least 1/week | 1.46 | (1.20, 1.78) | <.001 |
|  | 1-3/month | 1.65 | (1.33, 2.05) |  |
|  | <1/month | 1.36 | (1.08, 1.72) |  |
| Age group (Ref: 1998-2005; current age: 18-24) | 1993-1998; age: 25-29 | 1.01 | (0.76, 1.35) | <.001 |
|  | 1983-1993; age: 30-39 | 1.14 | (0.90, 1.44) |  |
|  | 1973-1983; age 40-49 | 1.03 | (0.81, 1.31) |  |
|  | 1963-1973; age 50-59 | 1.07 | (0.83, 1.39) |  |
|  | 1953-1963; age 60-69 | 0.83 | (0.60, 1.13) |  |
|  | 1943-1953; age 70-79 | 0.47 | (0.31, 0.71) |  |
|  | 1943 or earlier; age 80+ | 0.70 | (0.33, 1.50) |  |
| Gender (Ref: Male) | Female | 0.96 | (0.84, 1.11) | 1 |
|  | Other | 0.99 | (0.37, 2.65) |  |
| Religious affiliation at age 12 (Ref: No religion/Atheist/Agnostic) | Christianity | 1.00 | (0.76, 1.31) | 0.238 |
|  | Some other religion | 1.40 | (0.86, 2.29) |  |
| Race and ethnicity plurality (Ref: Majority) | Minority | 1.05 | (0.91, 1.21) | 0.72 |

***Table S1c. Sensitivity to unmeasured confounding of childhood predictors in Argentina***

| Variable | Category | E-value for Estimate | E-value for 95% CI |
| --- | --- | --- | --- |
| Relationship with mother (Ref: Very bad/Somewhat bad) | Very good/Somewhat good | 1.22 | 1.00 |
| Relationship with father (Ref: Very bad/Somewhat bad) | Very good/Somewhat good | 1.83 | 1.08 |
| Parent marital status (Ref: Parents married) | No, divorced | 1.92 | 1.09 |
|  | Never | 1.50 | 1.00 |
|  | No, one or both of them had died | 1.09 | 1.00 |
| Subjective financial status of family growing up (Ref: Got by) | Lived comfortably | 1.78 | 1.27 |
|  | Found it difficult | 1.53 | 1.00 |
|  | Found it very difficult | 1.97 | 1.07 |
| Abuse (Ref: No) | Yes | 1.65 | 1.04 |
| Outsider growing up (Ref: No) | Yes | 1.43 | 1.00 |
| Self-rated health growing up (Ref: Good) | Excellent | 1.74 | 1.11 |
|  | Very good | 1.69 | 1.00 |
|  | Fair | 2.29 | 1.50 |
|  | Poor | 2.07 | 1.00 |
| Immigration status (Ref: Born in this country) | Born in another country | 1.21 | 1.00 |
| Age 12 religious service attendance (Ref: Never) | At least 1/week | 2.29 | 1.70 |
|  | 1-3/month | 2.69 | 1.99 |
|  | <1/month | 2.07 | 1.39 |
| Age group (Ref: 1998-2005; current age: 18-24) | 1993-1998; age: 25-29 | 1.12 | 1.00 |
|  | 1983-1993; age: 30-39 | 1.54 | 1.00 |
|  | 1973-1983; age 40-49 | 1.21 | 1.00 |
|  | 1963-1973; age 50-59 | 1.35 | 1.00 |
|  | 1953-1963; age 60-69 | 1.73 | 1.00 |
|  | 1943-1953; age 70-79 | 3.68 | 2.16 |
|  | 1943 or earlier; age 80+ | 2.20 | 1.00 |
| Gender (Ref: Male) | Female | 1.23 | 1.00 |
|  | Other | 1.13 | 1.00 |
| Religious affiliation at age 12 (Ref: No religion/Atheist/Agnostic) | Christianity | 1.05 | 1.00 |
|  | Some other religion | 2.15 | 1.00 |
| Race and ethnicity plurality (Ref: Majority) | Minority | 1.28 | 1.00 |

***Table S2a. Nationally representative descriptive statistics for Australia***

| **Characteristic** | **N = 3,844**^1^ |
| --- | --- |
| **Relationship with mother** |  |
| Very good | 2,554 (66%) |
| Somewhat good | 925 (24%) |
| Somewhat bad | 218 (5.7%) |
| Very bad | 107 (2.8%) |
| Does not apply | 32 (0.8%) |
| Missing | 7 (0.2%) |
| **Relationship with father** |  |
| Very good | 2,032 (53%) |
| Somewhat good | 1,144 (30%) |
| Somewhat bad | 315 (8.2%) |
| Very bad | 196 (5.1%) |
| Does not apply | 148 (3.9%) |
| Missing | 9 (0.2%) |
| **Parent marital status** |  |
| Yes, married | 3,048 (79%) |
| No, divorced | 462 (12%) |
| Never married | 187 (4.9%) |
| No, one or both of them had died | 96 (2.5%) |
| Missing | 52 (1.4%) |
| **Subjective financial status of family growing up** |  |
| Lived comfortably | 1,756 (46%) |
| Got by | 1,496 (39%) |
| Found it difficult | 422 (11%) |
| Found it very difficult | 154 (4.0%) |
| Missing | 16 (0.4%) |
| **Abuse** |  |
| Yes | 995 (26%) |
| No | 2,790 (73%) |
| Missing | 59 (1.5%) |
| **Outsider growing up** |  |
| Yes | 756 (20%) |
| No | 3,062 (80%) |
| Missing | 26 (0.7%) |
| **Self-rated health growing up** |  |
| Excellent | 1,736 (45%) |
| Very good | 1,087 (28%) |
| Good | 603 (16%) |
| Fair | 308 (8.0%) |
| Poor | 106 (2.8%) |
| Missing | 4 (<0.1%) |
| **Immigration status** |  |
| Born in this country | 2,953 (77%) |
| Born in another country | 885 (23%) |
| Missing | 6 (0.2%) |
| **Age 12 religious service attendance** |  |
| At least 1/week | 1,362 (35%) |
| 1-3/month | 486 (13%) |
| <1/month | 600 (16%) |
| Never | 1,307 (34%) |
| Missing | 90 (2.3%) |
| **Age group** |  |
| 1998-2005; current age: 18-24 | 345 (9.0%) |
| 1993-1998; age: 25-29 | 282 (7.3%) |
| 1983-1993; age: 30-39 | 641 (17%) |
| 1973-1983; age 40-49 | 618 (16%) |
| 1963-1973; age 50-59 | 691 (18%) |
| 1953-1963; age 60-69 | 589 (15%) |
| 1943-1953; age 70-79 | 498 (13%) |
| 1943 or earlier; age 80+ | 178 (4.6%) |
| Missing | 2 (<0.1%) |
| **Gender** |  |
| Male | 1,861 (48%) |
| Female | 1,941 (50%) |
| Other | 36 (0.9%) |
| Missing | 6 (0.2%) |
| **Religious affiliation at age 12** |  |
| Baha’i | 5 (0.1%) |
| Buddhism | 16 (0.4%) |
| Christianity | 2,678 (70%) |
| Hinduism | 39 (1.0%) |
| Islam | 48 (1.2%) |
| Judaism | 29 (0.8%) |
| No religion/Atheist/Agnostic | 990 (26%) |
| Primal, Animist, or Folk religion | 4 (<0.1%) |
| Sikhism | 6 (0.2%) |
| Some other religion | 8 (0.2%) |
| Taoism | 1 (<0.1%) |
| Missing | 21 (0.5%) |
| **Race and ethnicity** |  |
| Aboriginal | 53 (1.4%) |
| Australian | 1,946 (51%) |
| Australian British/European | 1,047 (27%) |
| Chinese | 75 (1.9%) |
| Indian | 58 (1.5%) |
| Japanese | 1 (<0.1%) |
| Malay | 11 (0.3%) |
| New Zealander | 91 (2.4%) |
| Other | 163 (4.2%) |
| Other European | 357 (9.3%) |
| Russian | 7 (0.2%) |
| Samoan | 4 (0.1%) |
| Sinhalese | 1 (<0.1%) |
| Spanish | 2 (<0.1%) |
| Sri Lankan Moor | 1 (<0.1%) |
| Sri Lankan Tamil | 7 (0.2%) |
| Vietnamese | 7 (0.2%) |
| Missing | 14 (0.4%) |
| ^1^n (%) | |

***Table S2b. Regression of volunteering on childhood predictors for Australia***

| Variable | Category | Risk ratio | 95% CI | Global p-value |
| --- | --- | --- | --- | --- |
| Relationship with mother (Ref: Very bad/Somewhat bad) | Very good/Somewhat good | 1.03 | (0.82, 1.30) | 0.865 |
| Relationship with father (Ref: Very bad/Somewhat bad) | Very good/Somewhat good | 0.94 | (0.78, 1.13) | 0.161 |
| Parent marital status (Ref: Parents married) | No, divorced | 0.76 | (0.61, 0.97) | 0.032 |
|  | Never | 0.88 | (0.60, 1.28) |  |
|  | No, one or both of them had died | 0.92 | (0.63, 1.35) |  |
| Subjective financial status of family growing up (Ref: Got by) | Lived comfortably | 0.90 | (0.80, 1.01) | 0.346 |
|  | Found it difficult | 0.96 | (0.79, 1.17) |  |
|  | Found it very difficult | 1.02 | (0.75, 1.39) |  |
| Abuse (Ref: No) | Yes | 1.07 | (0.94, 1.23) | 0.212 |
| Outsider growing up (Ref: No) | Yes | 0.95 | (0.80, 1.12) | 0.806 |
| Self-rated health growing up (Ref: Good) | Excellent | 1.03 | (0.88, 1.22) | 0.038 |
|  | Very good | 0.89 | (0.74, 1.06) |  |
|  | Fair | 1.04 | (0.81, 1.34) |  |
|  | Poor | 1.15 | (0.79, 1.68) |  |
| Immigration status (Ref: Born in this country) | Born in another country | 0.87 | (0.75, 1.01) | 0.003 |
| Age 12 religious service attendance (Ref: Never) | At least 1/week | 1.45 | (1.23, 1.69) | <.001 |
|  | 1-3/month | 1.39 | (1.15, 1.69) |  |
|  | <1/month | 1.22 | (1.01, 1.49) |  |
| Age group (Ref: 1998-2005; current age: 18-24) | 1993-1998; age: 25-29 | 1.01 | (0.67, 1.51) | <.001 |
|  | 1983-1993; age: 30-39 | 1.09 | (0.79, 1.52) |  |
|  | 1973-1983; age 40-49 | 1.46 | (1.08, 1.98) |  |
|  | 1963-1973; age 50-59 | 1.15 | (0.84, 1.56) |  |
|  | 1953-1963; age 60-69 | 1.38 | (1.02, 1.87) |  |
|  | 1943-1953; age 70-79 | 1.51 | (1.10, 2.05) |  |
|  | 1943 or earlier; age 80+ | 1.54 | (1.09, 2.17) |  |
| Gender (Ref: Male) | Female | 1.05 | (0.94, 1.17) | 0.323 |
|  | Other | 0.58 | (0.24, 1.41) |  |
| Religious affiliation at age 12 (Ref: No religion/Atheist/Agnostic) | Christianity | 0.93 | (0.78, 1.11) | 0.445 |
|  | Some other religion | 0.77 | (0.53, 1.12) |  |
| Race and ethnicity plurality (Ref: Majority) | Minority | 1.08 | (0.96, 1.22) | 0.1 |

***Table S2c. Sensitivity to unmeasured confounding of childhood predictors in Australia***

| Variable | Category | E-value for Estimate | E-value for 95% CI |
| --- | --- | --- | --- |
| Relationship with mother (Ref: Very bad/Somewhat bad) | Very good/Somewhat good | 1.22 | 1.00 |
| Relationship with father (Ref: Very bad/Somewhat bad) | Very good/Somewhat good | 1.33 | 1.00 |
| Parent marital status (Ref: Parents married) | No, divorced | 1.93 | 1.22 |
|  | Never | 1.54 | 1.00 |
|  | No, one or both of them had died | 1.38 | 1.00 |
| Subjective financial status of family growing up (Ref: Got by) | Lived comfortably | 1.47 | 1.00 |
|  | Found it difficult | 1.23 | 1.00 |
|  | Found it very difficult | 1.18 | 1.00 |
| Abuse (Ref: No) | Yes | 1.37 | 1.00 |
| Outsider growing up (Ref: No) | Yes | 1.29 | 1.00 |
| Self-rated health growing up (Ref: Good) | Excellent | 1.22 | 1.00 |
|  | Very good | 1.52 | 1.00 |
|  | Fair | 1.25 | 1.00 |
|  | Poor | 1.56 | 1.00 |
| Immigration status (Ref: Born in this country) | Born in another country | 1.55 | 1.00 |
| Age 12 religious service attendance (Ref: Never) | At least 1/week | 2.24 | 1.77 |
|  | 1-3/month | 2.13 | 1.56 |
|  | <1/month | 1.75 | 1.12 |
| Age group (Ref: 1998-2005; current age: 18-24) | 1993-1998; age: 25-29 | 1.10 | 1.00 |
|  | 1983-1993; age: 30-39 | 1.43 | 1.00 |
|  | 1973-1983; age 40-49 | 2.28 | 1.36 |
|  | 1963-1973; age 50-59 | 1.56 | 1.00 |
|  | 1953-1963; age 60-69 | 2.11 | 1.16 |
|  | 1943-1953; age 70-79 | 2.38 | 1.44 |
|  | 1943 or earlier; age 80+ | 2.45 | 1.41 |
| Gender (Ref: Male) | Female | 1.27 | 1.00 |
|  | Other | 2.83 | 1.00 |
| Religious affiliation at age 12 (Ref: No religion/Atheist/Agnostic) | Christianity | 1.35 | 1.00 |
|  | Some other religion | 1.92 | 1.00 |
| Race and ethnicity plurality (Ref: Majority) | Minority | 1.38 | 1.00 |

***Table S3a. Nationally representative descriptive statistics for Brazil***

| **Characteristic** | **N = 13,204**^1^ |
| --- | --- |
| **Relationship with mother** |  |
| Very good | 8,369 (63%) |
| Somewhat good | 3,559 (27%) |
| Somewhat bad | 483 (3.7%) |
| Very bad | 214 (1.6%) |
| Does not apply | 507 (3.8%) |
| Missing | 73 (0.6%) |
| **Relationship with father** |  |
| Very good | 6,364 (48%) |
| Somewhat good | 3,654 (28%) |
| Somewhat bad | 1,035 (7.8%) |
| Very bad | 756 (5.7%) |
| Does not apply | 1,303 (9.9%) |
| Missing | 93 (0.7%) |
| **Parent marital status** |  |
| Yes, married | 8,546 (65%) |
| No, divorced | 1,384 (10%) |
| Never married | 1,985 (15%) |
| No, one or both of them had died | 508 (3.8%) |
| Missing | 781 (5.9%) |
| **Subjective financial status of family growing up** |  |
| Lived comfortably | 4,998 (38%) |
| Got by | 4,616 (35%) |
| Found it difficult | 2,484 (19%) |
| Found it very difficult | 1,027 (7.8%) |
| Missing | 79 (0.6%) |
| **Abuse** |  |
| Yes | 2,606 (20%) |
| No | 10,147 (77%) |
| Missing | 451 (3.4%) |
| **Outsider growing up** |  |
| Yes | 1,659 (13%) |
| No | 11,234 (85%) |
| Missing | 311 (2.4%) |
| **Self-rated health growing up** |  |
| Excellent | 5,312 (40%) |
| Very good | 3,392 (26%) |
| Good | 2,873 (22%) |
| Fair | 1,368 (10%) |
| Poor | 228 (1.7%) |
| Missing | 30 (0.2%) |
| **Immigration status** |  |
| Born in this country | 12,688 (96%) |
| Born in another country | 153 (1.2%) |
| Missing | 363 (2.7%) |
| **Age 12 religious service attendance** |  |
| At least 1/week | 6,306 (48%) |
| 1-3/month | 2,491 (19%) |
| <1/month | 2,629 (20%) |
| Never | 1,707 (13%) |
| Missing | 71 (0.5%) |
| **Age group** |  |
| 1998-2005; current age: 18-24 | 1,986 (15%) |
| 1993-1998; age: 25-29 | 1,468 (11%) |
| 1983-1993; age: 30-39 | 2,908 (22%) |
| 1973-1983; age 40-49 | 2,638 (20%) |
| 1963-1973; age 50-59 | 2,131 (16%) |
| 1953-1963; age 60-69 | 1,435 (11%) |
| 1943-1953; age 70-79 | 510 (3.9%) |
| 1943 or earlier; age 80+ | 126 (1.0%) |
| Missing | 0 (0%) |
| **Gender** |  |
| Male | 6,320 (48%) |
| Female | 6,820 (52%) |
| Other | 35 (0.3%) |
| Missing | 30 (0.2%) |
| **Religious affiliation at age 12** |  |
| Baha’i | 1 (<0.1%) |
| Buddhism | 27 (0.2%) |
| Christianity | 11,403 (86%) |
| Confucianism | 7 (<0.1%) |
| Hinduism | 1 (<0.1%) |
| Islam | 15 (0.1%) |
| Jainism | 4 (<0.1%) |
| Judaism | 40 (0.3%) |
| No religion/Atheist/Agnostic | 908 (6.9%) |
| Primal, Animist, or Folk religion | 17 (0.1%) |
| Shinto | 4 (<0.1%) |
| Some other religion | 87 (0.7%) |
| Spiritism | 336 (2.5%) |
| Taoism | 1 (<0.1%) |
| Umbanda, Candomblé, and other African-derived religions | 262 (2.0%) |
| Missing | 94 (0.7%) |
| **Race and ethnicity** |  |
| Amarela | 238 (1.8%) |
| Branca | 5,169 (39%) |
| Indígena | 131 (1.0%) |
| Other | 61 (0.5%) |
| Parda | 5,125 (39%) |
| Preta | 1,615 (12%) |
| Missing | 865 (6.6%) |
| ^1^n (%) | |

***Table S3b. Regression of volunteering on childhood predictors for Brazil***

| Variable | Category | Risk ratio | 95% CI | Global p-value |
| --- | --- | --- | --- | --- |
| Relationship with mother (Ref: Very bad/Somewhat bad) | Very good/Somewhat good | 1.04 | (0.87, 1.24) | 0.564 |
| Relationship with father (Ref: Very bad/Somewhat bad) | Very good/Somewhat good | 1.00 | (0.88, 1.14) | 0.999 |
| Parent marital status (Ref: Parents married) | No, divorced | 0.98 | (0.85, 1.14) | 1 |
|  | Never | 1.01 | (0.88, 1.17) |  |
|  | No, one or both of them had died | 0.97 | (0.74, 1.26) |  |
| Subjective financial status of family growing up (Ref: Got by) | Lived comfortably | 1.00 | (0.90, 1.12) | 0.999 |
|  | Found it difficult | 0.96 | (0.84, 1.11) |  |
|  | Found it very difficult | 0.93 | (0.76, 1.15) |  |
| Abuse (Ref: No) | Yes | 1.21 | (1.08, 1.35) | <.001 |
| Outsider growing up (Ref: No) | Yes | 1.12 | (0.99, 1.27) | 0.002 |
| Self-rated health growing up (Ref: Good) | Excellent | 1.14 | (1.00, 1.30) | 0.031 |
|  | Very good | 1.02 | (0.89, 1.17) |  |
|  | Fair | 0.98 | (0.82, 1.17) |  |
|  | Poor | 0.98 | (0.68, 1.41) |  |
| Immigration status (Ref: Born in this country) | Born in another country | 1.45 | (0.94, 2.23) | 0.007 |
| Age 12 religious service attendance (Ref: Never) | At least 1/week | 1.70 | (1.39, 2.07) | <.001 |
|  | 1-3/month | 1.54 | (1.25, 1.91) |  |
|  | <1/month | 1.32 | (1.06, 1.64) |  |
| Age group (Ref: 1998-2005; current age: 18-24) | 1993-1998; age: 25-29 | 0.84 | (0.72, 0.99) | 0.008 |
|  | 1983-1993; age: 30-39 | 0.82 | (0.71, 0.93) |  |
|  | 1973-1983; age 40-49 | 0.91 | (0.79, 1.06) |  |
|  | 1963-1973; age 50-59 | 0.92 | (0.78, 1.08) |  |
|  | 1953-1963; age 60-69 | 0.95 | (0.77, 1.17) |  |
|  | 1943-1953; age 70-79 | 0.70 | (0.48, 1.05) |  |
|  | 1943 or earlier; age 80+ | 1.08 | (0.57, 2.07) |  |
| Gender (Ref: Male) | Female | 1.01 | (0.92, 1.11) | 1 |
|  | Other | 0.90 | (0.53, 1.52) |  |
| Religious affiliation at age 12 (Ref: No religion/Atheist/Agnostic) | Christianity | 0.91 | (0.75, 1.11) | 0.003 |
|  | Some other religion | 1.11 | (0.86, 1.44) |  |
| Race and ethnicity plurality (Ref: Majority) | Minority | 1.03 | (0.94, 1.14) | 0.802 |

***Table S3c. Sensitivity to unmeasured confounding of childhood predictors in Brazil***

| Variable | Category | E-value for Estimate | E-value for 95% CI |
| --- | --- | --- | --- |
| Relationship with mother (Ref: Very bad/Somewhat bad) | Very good/Somewhat good | 1.24 | 1.00 |
| Relationship with father (Ref: Very bad/Somewhat bad) | Very good/Somewhat good | 1.04 | 1.00 |
| Parent marital status (Ref: Parents married) | No, divorced | 1.16 | 1.00 |
|  | Never | 1.13 | 1.00 |
|  | No, one or both of them had died | 1.22 | 1.00 |
| Subjective financial status of family growing up (Ref: Got by) | Lived comfortably | 1.05 | 1.00 |
|  | Found it difficult | 1.23 | 1.00 |
|  | Found it very difficult | 1.34 | 1.00 |
| Abuse (Ref: No) | Yes | 1.70 | 1.37 |
| Outsider growing up (Ref: No) | Yes | 1.49 | 1.00 |
| Self-rated health growing up (Ref: Good) | Excellent | 1.54 | 1.06 |
|  | Very good | 1.15 | 1.00 |
|  | Fair | 1.16 | 1.00 |
|  | Poor | 1.17 | 1.00 |
| Immigration status (Ref: Born in this country) | Born in another country | 2.25 | 1.00 |
| Age 12 religious service attendance (Ref: Never) | At least 1/week | 2.78 | 2.13 |
|  | 1-3/month | 2.46 | 1.81 |
|  | <1/month | 1.96 | 1.31 |
| Age group (Ref: 1998-2005; current age: 18-24) | 1993-1998; age: 25-29 | 1.66 | 1.09 |
|  | 1983-1993; age: 30-39 | 1.75 | 1.35 |
|  | 1973-1983; age 40-49 | 1.42 | 1.00 |
|  | 1963-1973; age 50-59 | 1.40 | 1.00 |
|  | 1953-1963; age 60-69 | 1.28 | 1.00 |
|  | 1943-1953; age 70-79 | 2.18 | 1.00 |
|  | 1943 or earlier; age 80+ | 1.39 | 1.00 |
| Gender (Ref: Male) | Female | 1.10 | 1.00 |
|  | Other | 1.46 | 1.00 |
| Religious affiliation at age 12 (Ref: No religion/Atheist/Agnostic) | Christianity | 1.43 | 1.00 |
|  | Some other religion | 1.46 | 1.00 |
| Race and ethnicity plurality (Ref: Majority) | Minority | 1.21 | 1.00 |

***Table S4a. Nationally representative descriptive statistics for Egypt***

| **Characteristic** | **N = 4,729**^1^ |
| --- | --- |
| **Relationship with mother** |  |
| Very good | 4,110 (87%) |
| Somewhat good | 505 (11%) |
| Somewhat bad | 21 (0.4%) |
| Very bad | 10 (0.2%) |
| Does not apply | 83 (1.8%) |
| Missing | 0 (0%) |
| **Relationship with father** |  |
| Very good | 3,713 (79%) |
| Somewhat good | 683 (14%) |
| Somewhat bad | 56 (1.2%) |
| Very bad | 30 (0.6%) |
| Does not apply | 233 (4.9%) |
| Missing | 14 (0.3%) |
| **Parent marital status** |  |
| Yes, married | 4,049 (86%) |
| No, divorced | 131 (2.8%) |
| Never married | 9 (0.2%) |
| No, one or both of them had died | 485 (10%) |
| Missing | 55 (1.2%) |
| **Subjective financial status of family growing up** |  |
| Lived comfortably | 1,251 (26%) |
| Got by | 2,352 (50%) |
| Found it difficult | 857 (18%) |
| Found it very difficult | 268 (5.7%) |
| Missing | 1 (<0.1%) |
| **Abuse** |  |
| Yes | 405 (8.6%) |
| No | 4,293 (91%) |
| Missing | 30 (0.6%) |
| **Outsider growing up** |  |
| Yes | 260 (5.5%) |
| No | 4,456 (94%) |
| Missing | 13 (0.3%) |
| **Self-rated health growing up** |  |
| Excellent | 2,687 (57%) |
| Very good | 1,174 (25%) |
| Good | 497 (11%) |
| Fair | 265 (5.6%) |
| Poor | 106 (2.2%) |
| Missing | 1 (<0.1%) |
| **Immigration status** |  |
| Born in this country | 4,713 (100%) |
| Born in another country | 16 (0.3%) |
| Missing | 1 (<0.1%) |
| **Age 12 religious service attendance** |  |
| At least 1/week | 2,307 (49%) |
| 1-3/month | 570 (12%) |
| <1/month | 629 (13%) |
| Never | 1,165 (25%) |
| Missing | 57 (1.2%) |
| **Age group** |  |
| 1998-2005; current age: 18-24 | 960 (20%) |
| 1993-1998; age: 25-29 | 607 (13%) |
| 1983-1993; age: 30-39 | 1,204 (25%) |
| 1973-1983; age 40-49 | 897 (19%) |
| 1963-1973; age 50-59 | 613 (13%) |
| 1953-1963; age 60-69 | 387 (8.2%) |
| 1943-1953; age 70-79 | 54 (1.1%) |
| 1943 or earlier; age 80+ | 7 (0.2%) |
| Missing | 0 (0%) |
| **Gender** |  |
| Male | 2,394 (51%) |
| Female | 2,334 (49%) |
| Other | 0 (0%) |
| Missing | 0 (<0.1%) |
| **Religious affiliation at age 12** |  |
| Christianity | 123 (2.6%) |
| Islam | 4,602 (97%) |
| Jainism | 1 (<0.1%) |
| Taoism | 0 (<0.1%) |
| Missing | 3 (<0.1%) |
| **Race and ethnicity** |  |
| Arab | 4,585 (97%) |
| Bedouin Arab | 4 (<0.1%) |
| Greek | 1 (<0.1%) |
| Nubian | 27 (0.6%) |
| Turkish | 9 (0.2%) |
| Missing | 102 (2.2%) |
| ^1^n (%) | |

***Table S4b. Regression of volunteering on childhood predictors for Egypt***

| Variable | Category | Risk ratio | 95% CI | Global p-value |
| --- | --- | --- | --- | --- |
| Relationship with mother (Ref: Very bad/Somewhat bad) | Very good/Somewhat good | 0.79 | (0.29, 2.18) | 0.593 |
| Relationship with father (Ref: Very bad/Somewhat bad) | Very good/Somewhat good | 2.05 | (0.18, 23.80) | 0.327 |
| Parent marital status (Ref: Parents married) | No, divorced | 0.60 | (0.18, 1.96) | 0.489 |
|  | Never | 3.16 | (0.48, 21.04) |  |
|  | No, one or both of them had died | 1.31 | (0.70, 2.44) |  |
| Subjective financial status of family growing up (Ref: Got by) | Lived comfortably | 1.48 | (1.04, 2.08) | 0.028 |
|  | Found it difficult | 1.00 | (0.60, 1.66) |  |
|  | Found it very difficult | 0.77 | (0.30, 1.95) |  |
| Abuse (Ref: No) | Yes | 0.68 | (0.37, 1.24) | 0.107 |
| Outsider growing up (Ref: No) | Yes | 1.43 | (0.85, 2.45) | 0.075 |
| Self-rated health growing up (Ref: Good) | Excellent | 0.99 | (0.59, 1.67) | 0.895 |
|  | Very good | 0.84 | (0.45, 1.58) |  |
|  | Fair | 1.46 | (0.71, 3.02) |  |
|  | Poor | 1.09 | (0.37, 3.22) |  |
| Immigration status (Ref: Born in this country) | Born in another country | 0.00 | (0.00, 0.00) | <.001 |
| Age 12 religious service attendance (Ref: Never) | At least 1/week | 1.99 | (1.36, 2.94) | <.001 |
|  | 1-3/month | 1.49 | (0.87, 2.58) |  |
|  | <1/month | 1.32 | (0.63, 2.78) |  |
| Age group (Ref: 1998-2005; current age: 18-24) | 1993-1998; age: 25-29 | 0.60 | (0.33, 1.11) | <.001 |
|  | 1983-1993; age: 30-39 | 0.62 | (0.38, 0.99) |  |
|  | 1973-1983; age 40-49 | 0.63 | (0.36, 1.10) |  |
|  | 1963-1973; age 50-59 | 0.73 | (0.40, 1.34) |  |
|  | 1953-1963; age 60-69 | 1.20 | (0.70, 2.06) |  |
|  | 1943-1953; age 70-79 | 0.75 | (0.19, 2.90) |  |
|  | 1943 or earlier; age 80+ | 0.00 | (0.00, 0.00) |  |
| Gender (Ref: Male) | Female | 0.70 | (0.52, 0.94) | <.001 |
| Religious affiliation at age 12 (Ref: Islam) | Some other religion | 1.67 | (0.92, 3.01) | 0.01 |
| Race and ethnicity plurality (Ref: Majority) | Minority | 2.14 | (0.87, 5.24) | 0.011 |

***Table S4c. Sensitivity to unmeasured confounding of childhood predictors in Egypt***

| Variable | Category | E-value for Estimate | E-value for 95% CI |
| --- | --- | --- | --- |
| Relationship with mother (Ref: Very bad/Somewhat bad) | Very good/Somewhat good | 1.82 | 1.00 |
| Relationship with father (Ref: Very bad/Somewhat bad) | Very good/Somewhat good | 3.53 | 1.00 |
| Parent marital status (Ref: Parents married) | No, divorced | 2.71 | 1.00 |
|  | Never | 5.78 | 1.00 |
|  | No, one or both of them had died | 1.94 | 1.00 |
| Subjective financial status of family growing up (Ref: Got by) | Lived comfortably | 2.31 | 1.25 |
|  | Found it difficult | 1.07 | 1.00 |
|  | Found it very difficult | 1.92 | 1.00 |
| Abuse (Ref: No) | Yes | 2.33 | 1.00 |
| Outsider growing up (Ref: No) | Yes | 2.24 | 1.00 |
| Self-rated health growing up (Ref: Good) | Excellent | 1.10 | 1.00 |
|  | Very good | 1.67 | 1.00 |
|  | Fair | 2.29 | 1.00 |
|  | Poor | 1.42 | 1.00 |
| Immigration status (Ref: Born in this country) | Born in another country | 766,149.49 | 301,000.14 |
| Age 12 religious service attendance (Ref: Never) | At least 1/week | 3.41 | 2.06 |
|  | 1-3/month | 2.35 | 1.00 |
|  | <1/month | 1.99 | 1.00 |
| Age group (Ref: 1998-2005; current age: 18-24) | 1993-1998; age: 25-29 | 2.72 | 1.00 |
|  | 1983-1993; age: 30-39 | 2.63 | 1.10 |
|  | 1973-1983; age 40-49 | 2.56 | 1.00 |
|  | 1963-1973; age 50-59 | 2.06 | 1.00 |
|  | 1953-1963; age 60-69 | 1.68 | 1.00 |
|  | 1943-1953; age 70-79 | 2.00 | 1.00 |
|  | 1943 or earlier; age 80+ | 890,617.62 | 245,607.45 |
| Gender (Ref: Male) | Female | 2.23 | 1.34 |
| Religious affiliation at age 12 (Ref: Islam) | Some other religion | 2.71 | 1.00 |
| Race and ethnicity plurality (Ref: Majority) | Minority | 3.70 | 1.00 |

***Table S5a. Nationally representative descriptive statistics for Germany***

| **Characteristic** | **N = 9,506**^1^ |
| --- | --- |
| **Relationship with mother** |  |
| Very good | 5,497 (58%) |
| Somewhat good | 3,031 (32%) |
| Somewhat bad | 496 (5.2%) |
| Very bad | 187 (2.0%) |
| Does not apply | 241 (2.5%) |
| Missing | 54 (0.6%) |
| **Relationship with father** |  |
| Very good | 4,652 (49%) |
| Somewhat good | 3,012 (32%) |
| Somewhat bad | 846 (8.9%) |
| Very bad | 385 (4.0%) |
| Does not apply | 538 (5.7%) |
| Missing | 73 (0.8%) |
| **Parent marital status** |  |
| Yes, married | 7,620 (80%) |
| No, divorced | 927 (9.8%) |
| Never married | 578 (6.1%) |
| No, one or both of them had died | 245 (2.6%) |
| Missing | 136 (1.4%) |
| **Subjective financial status of family growing up** |  |
| Lived comfortably | 3,177 (33%) |
| Got by | 4,508 (47%) |
| Found it difficult | 1,481 (16%) |
| Found it very difficult | 314 (3.3%) |
| Missing | 26 (0.3%) |
| **Abuse** |  |
| Yes | 1,086 (11%) |
| No | 8,321 (88%) |
| Missing | 99 (1.0%) |
| **Outsider growing up** |  |
| Yes | 1,105 (12%) |
| No | 8,262 (87%) |
| Missing | 139 (1.5%) |
| **Self-rated health growing up** |  |
| Excellent | 2,633 (28%) |
| Very good | 3,518 (37%) |
| Good | 2,582 (27%) |
| Fair | 612 (6.4%) |
| Poor | 134 (1.4%) |
| Missing | 26 (0.3%) |
| **Immigration status** |  |
| Born in this country | 8,722 (92%) |
| Born in another country | 744 (7.8%) |
| Missing | 40 (0.4%) |
| **Age 12 religious service attendance** |  |
| At least 1/week | 1,943 (20%) |
| 1-3/month | 1,899 (20%) |
| <1/month | 2,887 (30%) |
| Never | 2,749 (29%) |
| Missing | 27 (0.3%) |
| **Age group** |  |
| 1998-2005; current age: 18-24 | 829 (8.7%) |
| 1993-1998; age: 25-29 | 774 (8.1%) |
| 1983-1993; age: 30-39 | 1,438 (15%) |
| 1973-1983; age 40-49 | 1,494 (16%) |
| 1963-1973; age 50-59 | 1,729 (18%) |
| 1953-1963; age 60-69 | 1,915 (20%) |
| 1943-1953; age 70-79 | 1,137 (12%) |
| 1943 or earlier; age 80+ | 190 (2.0%) |
| Missing | 0 (0%) |
| **Gender** |  |
| Male | 4,641 (49%) |
| Female | 4,843 (51%) |
| Other | 11 (0.1%) |
| Missing | 11 (0.1%) |
| **Religious affiliation at age 12** |  |
| Baha’i | 2 (<0.1%) |
| Buddhism | 25 (0.3%) |
| Christianity | 5,751 (61%) |
| Confucianism | 4 (<0.1%) |
| Hinduism | 15 (0.2%) |
| Islam | 350 (3.7%) |
| Jainism | 1 (<0.1%) |
| Judaism | 18 (0.2%) |
| No religion/Atheist/Agnostic | 3,163 (33%) |
| Primal, Animist, or Folk religion | 19 (0.2%) |
| Sikhism | 5 (<0.1%) |
| Some other religion | 67 (0.7%) |
| Missing | 85 (0.9%) |
| **Race and ethnicity** |  |
| Missing | 9,506 (100%) |
| ^1^n (%) | |

***Table S5b. Regression of volunteering on childhood predictors for Germany***

| Variable | Category | Risk ratio | 95% CI | Global p-value |
| --- | --- | --- | --- | --- |
| Relationship with mother (Ref: Very bad/Somewhat bad) | Very good/Somewhat good | 1.19 | (0.97, 1.44) | <.001 |
| Relationship with father (Ref: Very bad/Somewhat bad) | Very good/Somewhat good | 1.14 | (0.98, 1.34) | <.001 |
| Parent marital status (Ref: Parents married) | No, divorced | 1.02 | (0.86, 1.21) | 0.99 |
|  | Never | 1.01 | (0.82, 1.26) |  |
|  | No, one or both of them had died | 1.15 | (0.87, 1.51) |  |
| Subjective financial status of family growing up (Ref: Got by) | Lived comfortably | 1.00 | (0.88, 1.13) | 0.999 |
|  | Found it difficult | 0.95 | (0.82, 1.11) |  |
|  | Found it very difficult | 0.90 | (0.66, 1.22) |  |
| Abuse (Ref: No) | Yes | 1.22 | (1.06, 1.41) | <.001 |
| Outsider growing up (Ref: No) | Yes | 1.17 | (1.02, 1.35) | <.001 |
| Self-rated health growing up (Ref: Good) | Excellent | 0.96 | (0.83, 1.12) | 0.01 |
|  | Very good | 1.04 | (0.92, 1.19) |  |
|  | Fair | 1.23 | (1.01, 1.51) |  |
|  | Poor | 1.34 | (0.94, 1.89) |  |
| Immigration status (Ref: Born in this country) | Born in another country | 0.93 | (0.76, 1.16) | 0.798 |
| Age 12 religious service attendance (Ref: Never) | At least 1/week | 1.21 | (1.04, 1.39) | 0.009 |
|  | 1-3/month | 1.12 | (0.96, 1.29) |  |
|  | <1/month | 1.11 | (0.96, 1.27) |  |
| Age group (Ref: 1998-2005; current age: 18-24) | 1993-1998; age: 25-29 | 1.20 | (0.95, 1.50) | 0.016 |
|  | 1983-1993; age: 30-39 | 1.11 | (0.90, 1.36) |  |
|  | 1973-1983; age 40-49 | 1.11 | (0.89, 1.36) |  |
|  | 1963-1973; age 50-59 | 0.92 | (0.75, 1.14) |  |
|  | 1953-1963; age 60-69 | 0.97 | (0.79, 1.21) |  |
|  | 1943-1953; age 70-79 | 0.94 | (0.75, 1.20) |  |
|  | 1943 or earlier; age 80+ | 0.93 | (0.65, 1.34) |  |
| Gender (Ref: Male) | Female | 0.69 | (0.62, 0.77) | <.001 |
|  | Other | 1.15 | (0.35, 3.78) |  |
| Religious affiliation at age 12 (Ref: No religion/Atheist/Agnostic) | Christianity | 2.46 | (2.11, 2.88) | <.001 |
|  | Some other religion | 1.93 | (1.45, 2.58) |  |

***Table S5c. Sensitivity to unmeasured confounding of childhood predictors in Germany***

| Variable | Category | E-value for Estimate | E-value for 95% CI |
| --- | --- | --- | --- |
| Relationship with mother (Ref: Very bad/Somewhat bad) | Very good/Somewhat good | 1.65 | 1.00 |
| Relationship with father (Ref: Very bad/Somewhat bad) | Very good/Somewhat good | 1.55 | 1.00 |
| Parent marital status (Ref: Parents married) | No, divorced | 1.17 | 1.00 |
|  | Never | 1.14 | 1.00 |
|  | No, one or both of them had died | 1.56 | 1.00 |
| Subjective financial status of family growing up (Ref: Got by) | Lived comfortably | 1.04 | 1.00 |
|  | Found it difficult | 1.27 | 1.00 |
|  | Found it very difficult | 1.47 | 1.00 |
| Abuse (Ref: No) | Yes | 1.74 | 1.32 |
| Outsider growing up (Ref: No) | Yes | 1.63 | 1.18 |
| Self-rated health growing up (Ref: Good) | Excellent | 1.25 | 1.00 |
|  | Very good | 1.26 | 1.00 |
|  | Fair | 1.77 | 1.11 |
|  | Poor | 2.00 | 1.00 |
| Immigration status (Ref: Born in this country) | Born in another country | 1.34 | 1.00 |
| Age 12 religious service attendance (Ref: Never) | At least 1/week | 1.70 | 1.25 |
|  | 1-3/month | 1.47 | 1.00 |
|  | <1/month | 1.45 | 1.00 |
| Age group (Ref: 1998-2005; current age: 18-24) | 1993-1998; age: 25-29 | 1.67 | 1.00 |
|  | 1983-1993; age: 30-39 | 1.45 | 1.00 |
|  | 1973-1983; age 40-49 | 1.44 | 1.00 |
|  | 1963-1973; age 50-59 | 1.38 | 1.00 |
|  | 1953-1963; age 60-69 | 1.20 | 1.00 |
|  | 1943-1953; age 70-79 | 1.30 | 1.00 |
|  | 1943 or earlier; age 80+ | 1.35 | 1.00 |
| Gender (Ref: Male) | Female | 2.25 | 1.94 |
|  | Other | 1.58 | 1.00 |
| Religious affiliation at age 12 (Ref: No religion/Atheist/Agnostic) | Christianity | 4.36 | 3.63 |
|  | Some other religion | 3.27 | 2.25 |

***Table S6a. Nationally representative descriptive statistics for Hong Kong***

| **Characteristic** | **N = 3,012**^1^ |
| --- | --- |
| **Relationship with mother** |  |
| Very good | 1,077 (36%) |
| Somewhat good | 1,164 (39%) |
| Somewhat bad | 293 (9.7%) |
| Very bad | 49 (1.6%) |
| Does not apply | 426 (14%) |
| Missing | 3 (<0.1%) |
| **Relationship with father** |  |
| Very good | 868 (29%) |
| Somewhat good | 1,089 (36%) |
| Somewhat bad | 393 (13%) |
| Very bad | 102 (3.4%) |
| Does not apply | 557 (19%) |
| Missing | 3 (0.1%) |
| **Parent marital status** |  |
| Yes, married | 2,752 (91%) |
| No, divorced | 114 (3.8%) |
| Never married | 40 (1.3%) |
| No, one or both of them had died | 50 (1.7%) |
| Missing | 56 (1.8%) |
| **Subjective financial status of family growing up** |  |
| Lived comfortably | 906 (30%) |
| Got by | 1,527 (51%) |
| Found it difficult | 473 (16%) |
| Found it very difficult | 84 (2.8%) |
| Missing | 22 (0.7%) |
| **Abuse** |  |
| Yes | 318 (11%) |
| No | 2,688 (89%) |
| Missing | 5 (0.2%) |
| **Outsider growing up** |  |
| Yes | 664 (22%) |
| No | 2,224 (74%) |
| Missing | 124 (4.1%) |
| **Self-rated health growing up** |  |
| Excellent | 545 (18%) |
| Very good | 1,073 (36%) |
| Good | 863 (29%) |
| Fair | 426 (14%) |
| Poor | 91 (3.0%) |
| Missing | 13 (0.4%) |
| **Immigration status** |  |
| Born in this country | 2,637 (88%) |
| Born in another country | 321 (11%) |
| Missing | 53 (1.8%) |
| **Age 12 religious service attendance** |  |
| At least 1/week | 432 (14%) |
| 1-3/month | 528 (18%) |
| <1/month | 753 (25%) |
| Never | 1,295 (43%) |
| Missing | 4 (0.1%) |
| **Age group** |  |
| 1998-2005; current age: 18-24 | 217 (7.2%) |
| 1993-1998; age: 25-29 | 198 (6.6%) |
| 1983-1993; age: 30-39 | 507 (17%) |
| 1973-1983; age 40-49 | 580 (19%) |
| 1963-1973; age 50-59 | 711 (24%) |
| 1953-1963; age 60-69 | 620 (21%) |
| 1943-1953; age 70-79 | 164 (5.5%) |
| 1943 or earlier; age 80+ | 15 (0.5%) |
| Missing | 0 (0%) |
| **Gender** |  |
| Male | 1,390 (46%) |
| Female | 1,620 (54%) |
| Other | 2 (<0.1%) |
| Missing | 0 (0%) |
| **Religious affiliation at age 12** |  |
| Buddhism | 323 (11%) |
| Chinese folk/traditional religion | 108 (3.6%) |
| Christianity | 715 (24%) |
| Confucianism | 10 (0.3%) |
| Hinduism | 27 (0.9%) |
| Islam | 86 (2.9%) |
| Jainism | 1 (<0.1%) |
| Judaism | 16 (0.5%) |
| No religion/Atheist/Agnostic | 1,601 (53%) |
| Primal, Animist, or Folk religion | 15 (0.5%) |
| Shinto | 18 (0.6%) |
| Sikhism | 4 (0.1%) |
| Some other religion | 5 (0.2%) |
| Taoism | 81 (2.7%) |
| Missing | 1 (<0.1%) |
| **Race and ethnicity** |  |
| Chinese (Cantonese) | 1,930 (64%) |
| Chinese (Chaoshan) | 201 (6.7%) |
| Chinese (Fujianese) | 117 (3.9%) |
| Chinese (Hakka) | 121 (4.0%) |
| Chinese (Other ethnicity) | 264 (8.8%) |
| Chinese (Shanghainese) | 89 (2.9%) |
| East Asian (Korean, Japanese) | 10 (0.3%) |
| Other | 4 (0.1%) |
| South Asian (Indian, Nepalese, Pakistani) | 17 (0.6%) |
| Southeast Asian (Filipino, Indonesian, Thailand) | 46 (1.5%) |
| Taiwanese | 14 (0.4%) |
| White | 15 (0.5%) |
| Missing | 184 (6.1%) |
| ^1^n (%) | |

***Table S6b. Regression of volunteering on childhood predictors for Hong Kong***

| Variable | Category | Risk ratio | 95% CI | Global p-value |
| --- | --- | --- | --- | --- |
| Relationship with mother (Ref: Very bad/Somewhat bad) | Very good/Somewhat good | 0.79 | (0.63, 1.00) | <.001 |
| Relationship with father (Ref: Very bad/Somewhat bad) | Very good/Somewhat good | 0.98 | (0.79, 1.20) | 0.939 |
| Parent marital status (Ref: Parents married) | No, divorced | 1.02 | (0.64, 1.63) | 0.947 |
|  | Never | 1.13 | (0.57, 2.22) |  |
|  | No, one or both of them had died | 1.62 | (0.69, 3.83) |  |
| Subjective financial status of family growing up (Ref: Got by) | Lived comfortably | 1.28 | (1.12, 1.49) | <.001 |
|  | Found it difficult | 0.76 | (0.54, 1.07) |  |
|  | Found it very difficult | 0.45 | (0.16, 1.26) |  |
| Abuse (Ref: No) | Yes | 1.26 | (1.07, 1.49) | <.001 |
| Outsider growing up (Ref: No) | Yes | 1.30 | (1.13, 1.50) | <.001 |
| Self-rated health growing up (Ref: Good) | Excellent | 1.55 | (1.23, 1.96) | <.001 |
|  | Very good | 1.40 | (1.14, 1.75) |  |
|  | Fair | 0.80 | (0.54, 1.19) |  |
|  | Poor | 0.58 | (0.28, 1.21) |  |
| Immigration status (Ref: Born in this country) | Born in another country | 0.89 | (0.66, 1.20) | 0.602 |
| Age 12 religious service attendance (Ref: Never) | At least 1/week | 1.45 | (1.12, 1.89) | <.001 |
|  | 1-3/month | 1.52 | (1.21, 1.93) |  |
|  | <1/month | 1.01 | (0.78, 1.30) |  |
| Age group (Ref: 1998-2005; current age: 18-24) | 1993-1998; age: 25-29 | 0.79 | (0.57, 1.10) | <.001 |
|  | 1983-1993; age: 30-39 | 0.78 | (0.61, 1.00) |  |
|  | 1973-1983; age 40-49 | 0.92 | (0.74, 1.15) |  |
|  | 1963-1973; age 50-59 | 1.31 | (1.06, 1.61) |  |
|  | 1953-1963; age 60-69 | 1.21 | (0.92, 1.58) |  |
|  | 1943-1953; age 70-79 | 1.07 | (0.63, 1.81) |  |
|  | 1943 or earlier; age 80+ | 0.00 | (0.00, 0.00) |  |
| Gender (Ref: Male) | Female | 1.01 | (0.89, 1.15) | <.001 |
|  | Other | 0.00 | (0.00, 0.00) |  |
| Religious affiliation at age 12 (Ref: No religion/Atheist/Agnostic) | Buddhism | 1.65 | (1.28, 2.11) | <.001 |
|  | Chinese folk/traditional religion | 1.84 | (1.41, 2.40) |  |
|  | Christianity | 1.46 | (1.17, 1.84) |  |
|  | Some other religion | 1.93 | (1.51, 2.49) |  |
| Race and ethnicity plurality (Ref: Majority) | Minority | 1.09 | (0.96, 1.26) | 0.08 |

***Table S6c. Sensitivity to unmeasured confounding of childhood predictors in Hong Kong***

| Variable | Category | E-value for Estimate | E-value for 95% CI |
| --- | --- | --- | --- |
| Relationship with mother (Ref: Very bad/Somewhat bad) | Very good/Somewhat good | 1.84 | 1.06 |
| Relationship with father (Ref: Very bad/Somewhat bad) | Very good/Somewhat good | 1.18 | 1.00 |
| Parent marital status (Ref: Parents married) | No, divorced | 1.16 | 1.00 |
|  | Never | 1.51 | 1.00 |
|  | No, one or both of them had died | 2.62 | 1.00 |
| Subjective financial status of family growing up (Ref: Got by) | Lived comfortably | 1.90 | 1.48 |
|  | Found it difficult | 1.95 | 1.00 |
|  | Found it very difficult | 3.83 | 1.00 |
| Abuse (Ref: No) | Yes | 1.84 | 1.36 |
| Outsider growing up (Ref: No) | Yes | 1.93 | 1.51 |
| Self-rated health growing up (Ref: Good) | Excellent | 2.49 | 1.77 |
|  | Very good | 2.17 | 1.54 |
|  | Fair | 1.80 | 1.00 |
|  | Poor | 2.86 | 1.00 |
| Immigration status (Ref: Born in this country) | Born in another country | 1.50 | 1.00 |
| Age 12 religious service attendance (Ref: Never) | At least 1/week | 2.27 | 1.49 |
|  | 1-3/month | 2.43 | 1.72 |
|  | <1/month | 1.08 | 1.00 |
| Age group (Ref: 1998-2005; current age: 18-24) | 1993-1998; age: 25-29 | 1.83 | 1.00 |
|  | 1983-1993; age: 30-39 | 1.89 | 1.07 |
|  | 1973-1983; age 40-49 | 1.38 | 1.00 |
|  | 1963-1973; age 50-59 | 1.94 | 1.32 |
|  | 1953-1963; age 60-69 | 1.70 | 1.00 |
|  | 1943-1953; age 70-79 | 1.35 | 1.00 |
|  | 1943 or earlier; age 80+ | 3,029,317.08 | 912,732.91 |
| Gender (Ref: Male) | Female | 1.13 | 1.00 |
|  | Other | 1,720,468.57 | 231,492.58 |
| Religious affiliation at age 12 (Ref: No religion/Atheist/Agnostic) | Christianity | 2.30 | 1.62 |
|  | Some other religion | 3.28 | 2.38 |
| Race and ethnicity plurality (Ref: Majority) | Minority | 1.42 | 1.00 |
|  | Buddhism | 2.67 | 1.87 |
|  | Chinese folk/traditional religion | 3.08 | 2.17 |

***Table S7a. Nationally representative descriptive statistics for India***

| **Characteristic** | **N = 12,765**^1^ |
| --- | --- |
| **Relationship with mother** |  |
| Very good | 11,465 (90%) |
| Somewhat good | 788 (6.2%) |
| Somewhat bad | 88 (0.7%) |
| Very bad | 73 (0.6%) |
| Does not apply | 269 (2.1%) |
| Missing | 82 (0.6%) |
| **Relationship with father** |  |
| Very good | 10,923 (86%) |
| Somewhat good | 995 (7.8%) |
| Somewhat bad | 126 (1.0%) |
| Very bad | 100 (0.8%) |
| Does not apply | 481 (3.8%) |
| Missing | 141 (1.1%) |
| **Parent marital status** |  |
| Yes, married | 5,578 (44%) |
| No, divorced | 236 (1.8%) |
| Never married | 1,055 (8.3%) |
| No, one or both of them had died | 940 (7.4%) |
| Missing | 4,956 (39%) |
| **Subjective financial status of family growing up** |  |
| Lived comfortably | 4,946 (39%) |
| Got by | 3,010 (24%) |
| Found it difficult | 2,703 (21%) |
| Found it very difficult | 2,035 (16%) |
| Missing | 70 (0.5%) |
| **Abuse** |  |
| Yes | 1,468 (11%) |
| No | 10,526 (82%) |
| Missing | 771 (6.0%) |
| **Outsider growing up** |  |
| Yes | 1,926 (15%) |
| No | 10,780 (84%) |
| Missing | 59 (0.5%) |
| **Self-rated health growing up** |  |
| Excellent | 2,182 (17%) |
| Very good | 3,882 (30%) |
| Good | 4,028 (32%) |
| Fair | 2,202 (17%) |
| Poor | 424 (3.3%) |
| Missing | 47 (0.4%) |
| **Immigration status** |  |
| Born in this country | 12,629 (99%) |
| Born in another country | 110 (0.9%) |
| Missing | 26 (0.2%) |
| **Age 12 religious service attendance** |  |
| At least 1/week | 5,288 (41%) |
| 1-3/month | 2,959 (23%) |
| <1/month | 2,719 (21%) |
| Never | 1,478 (12%) |
| Missing | 321 (2.5%) |
| **Age group** |  |
| 1998-2005; current age: 18-24 | 2,543 (20%) |
| 1993-1998; age: 25-29 | 1,640 (13%) |
| 1983-1993; age: 30-39 | 3,109 (24%) |
| 1973-1983; age 40-49 | 2,275 (18%) |
| 1963-1973; age 50-59 | 1,574 (12%) |
| 1953-1963; age 60-69 | 1,188 (9.3%) |
| 1943-1953; age 70-79 | 370 (2.9%) |
| 1943 or earlier; age 80+ | 67 (0.5%) |
| Missing | 0 (0%) |
| **Gender** |  |
| Male | 6,473 (51%) |
| Female | 6,292 (49%) |
| Other | 0 (0%) |
| Missing | 0 (0%) |
| **Religious affiliation at age 12** |  |
| Buddhism | 180 (1.4%) |
| Christianity | 254 (2.0%) |
| Hinduism | 10,417 (82%) |
| Islam | 1,550 (12%) |
| Jainism | 9 (<0.1%) |
| No religion/Atheist/Agnostic | 7 (<0.1%) |
| Primal, Animist, or Folk religion | 27 (0.2%) |
| Shinto | 4 (<0.1%) |
| Sikhism | 126 (1.0%) |
| Some other religion | 59 (0.5%) |
| Missing | 131 (1.0%) |
| **Race and ethnicity** |  |
| General | 3,538 (28%) |
| Other backward caste | 4,177 (33%) |
| Schedule caste | 3,599 (28%) |
| Schedule tribe | 1,185 (9.3%) |
| Missing | 267 (2.1%) |
| ^1^n (%) | |

***Table S7b. Regression of volunteering on childhood predictors for India***

| Variable | Category | Risk ratio | 95% CI | Global p-value |
| --- | --- | --- | --- | --- |
| Relationship with mother (Ref: Very bad/Somewhat bad) | Very good/Somewhat good | 1.17 | (0.86, 1.59) | 0.009 |
| Relationship with father (Ref: Very bad/Somewhat bad) | Very good/Somewhat good | 1.28 | (1.01, 1.62) | <.001 |
| Parent marital status (Ref: Parents married) | No, divorced | 0.92 | (0.74, 1.16) | 0.254 |
|  | Never | 0.93 | (0.82, 1.05) |  |
|  | No, one or both of them had died | 0.92 | (0.81, 1.05) |  |
| Subjective financial status of family growing up (Ref: Got by) | Lived comfortably | 1.15 | (1.04, 1.28) | <.001 |
|  | Found it difficult | 0.99 | (0.88, 1.11) |  |
|  | Found it very difficult | 1.01 | (0.86, 1.18) |  |
| Abuse (Ref: No) | Yes | 1.19 | (1.07, 1.31) | <.001 |
| Outsider growing up (Ref: No) | Yes | 1.34 | (1.20, 1.50) | <.001 |
| Self-rated health growing up (Ref: Good) | Excellent | 1.11 | (0.96, 1.27) | <.001 |
|  | Very good | 1.22 | (1.09, 1.38) |  |
|  | Fair | 1.22 | (1.09, 1.37) |  |
|  | Poor | 1.40 | (1.14, 1.72) |  |
| Immigration status (Ref: Born in this country) | Born in another country | 0.56 | (0.33, 0.95) | <.001 |
| Age 12 religious service attendance (Ref: Never) | At least 1/week | 1.43 | (1.23, 1.68) | <.001 |
|  | 1-3/month | 1.46 | (1.26, 1.72) |  |
|  | <1/month | 1.31 | (1.11, 1.54) |  |
| Age group (Ref: 1998-2005; current age: 18-24) | 1993-1998; age: 25-29 | 1.03 | (0.92, 1.15) | 0.129 |
|  | 1983-1993; age: 30-39 | 1.06 | (0.97, 1.18) |  |
|  | 1973-1983; age 40-49 | 1.05 | (0.95, 1.17) |  |
|  | 1963-1973; age 50-59 | 0.94 | (0.83, 1.07) |  |
|  | 1953-1963; age 60-69 | 0.92 | (0.79, 1.07) |  |
|  | 1943-1953; age 70-79 | 0.89 | (0.70, 1.13) |  |
|  | 1943 or earlier; age 80+ | 0.87 | (0.50, 1.50) |  |
| Gender (Ref: Male) | Female | 0.84 | (0.78, 0.90) | <.001 |
| Religious affiliation at age 12 (Ref: Hinduism) | Islam | 1.07 | (0.89, 1.28) | 0.938 |
|  | Some other religion | 0.94 | (0.76, 1.17) |  |
| Race and ethnicity plurality (Ref: Majority) | Minority | 0.93 | (0.85, 1.03) | 0.089 |

***Table S7c. Sensitivity to unmeasured confounding of childhood predictors in India***

| Variable | Category | E-value for Estimate | E-value for 95% CI |
| --- | --- | --- | --- |
| Relationship with mother (Ref: Very bad/Somewhat bad) | Very good/Somewhat good | 1.61 | 1.00 |
| Relationship with father (Ref: Very bad/Somewhat bad) | Very good/Somewhat good | 1.88 | 1.10 |
| Parent marital status (Ref: Parents married) | No, divorced | 1.37 | 1.00 |
|  | Never | 1.37 | 1.00 |
|  | No, one or both of them had died | 1.38 | 1.00 |
| Subjective financial status of family growing up (Ref: Got by) | Lived comfortably | 1.58 | 1.24 |
|  | Found it difficult | 1.09 | 1.00 |
|  | Found it very difficult | 1.08 | 1.00 |
| Abuse (Ref: No) | Yes | 1.65 | 1.34 |
| Outsider growing up (Ref: No) | Yes | 2.02 | 1.70 |
| Self-rated health growing up (Ref: Good) | Excellent | 1.44 | 1.00 |
|  | Very good | 1.74 | 1.39 |
|  | Fair | 1.74 | 1.39 |
|  | Poor | 2.15 | 1.54 |
| Immigration status (Ref: Born in this country) | Born in another country | 2.97 | 1.30 |
| Age 12 religious service attendance (Ref: Never) | At least 1/week | 2.23 | 1.77 |
|  | 1-3/month | 2.30 | 1.82 |
|  | <1/month | 1.95 | 1.47 |
| Age group (Ref: 1998-2005; current age: 18-24) | 1993-1998; age: 25-29 | 1.20 | 1.00 |
|  | 1983-1993; age: 30-39 | 1.33 | 1.00 |
|  | 1973-1983; age 40-49 | 1.30 | 1.00 |
|  | 1963-1973; age 50-59 | 1.31 | 1.00 |
|  | 1953-1963; age 60-69 | 1.40 | 1.00 |
|  | 1943-1953; age 70-79 | 1.49 | 1.00 |
|  | 1943 or earlier; age 80+ | 1.57 | 1.00 |
| Gender (Ref: Male) | Female | 1.68 | 1.47 |
| Religious affiliation at age 12 (Ref: Hinduism) | Some other religion | 1.32 | 1.00 |
| Race and ethnicity plurality (Ref: Majority) | Minority | 1.34 | 1.00 |
|  | Islam | 1.34 | 1.00 |

***Table S8a. Nationally representative descriptive statistics for Indonesia***

| **Characteristic** | **N = 6,992**^1^ |
| --- | --- |
| **Relationship with mother** |  |
| Very good | 6,238 (89%) |
| Somewhat good | 583 (8.3%) |
| Somewhat bad | 50 (0.7%) |
| Very bad | 26 (0.4%) |
| Does not apply | 68 (1.0%) |
| Missing | 27 (0.4%) |
| **Relationship with father** |  |
| Very good | 6,067 (87%) |
| Somewhat good | 628 (9.0%) |
| Somewhat bad | 68 (1.0%) |
| Very bad | 52 (0.7%) |
| Does not apply | 115 (1.6%) |
| Missing | 61 (0.9%) |
| **Parent marital status** |  |
| Yes, married | 5,557 (79%) |
| No, divorced | 448 (6.4%) |
| Never married | 47 (0.7%) |
| No, one or both of them had died | 735 (11%) |
| Missing | 205 (2.9%) |
| **Subjective financial status of family growing up** |  |
| Lived comfortably | 3,408 (49%) |
| Got by | 2,955 (42%) |
| Found it difficult | 439 (6.3%) |
| Found it very difficult | 181 (2.6%) |
| Missing | 9 (0.1%) |
| **Abuse** |  |
| Yes | 486 (6.9%) |
| No | 6,427 (92%) |
| Missing | 79 (1.1%) |
| **Outsider growing up** |  |
| Yes | 343 (4.9%) |
| No | 6,639 (95%) |
| Missing | 10 (0.1%) |
| **Self-rated health growing up** |  |
| Excellent | 1,246 (18%) |
| Very good | 1,968 (28%) |
| Good | 2,490 (36%) |
| Fair | 1,233 (18%) |
| Poor | 55 (0.8%) |
| Missing | 1 (<0.1%) |
| **Immigration status** |  |
| Born in this country | 6,958 (100%) |
| Born in another country | 34 (0.5%) |
| Missing | 0 (0%) |
| **Age 12 religious service attendance** |  |
| At least 1/week | 5,363 (77%) |
| 1-3/month | 973 (14%) |
| <1/month | 329 (4.7%) |
| Never | 275 (3.9%) |
| Missing | 51 (0.7%) |
| **Age group** |  |
| 1998-2005; current age: 18-24 | 1,216 (17%) |
| 1993-1998; age: 25-29 | 849 (12%) |
| 1983-1993; age: 30-39 | 1,591 (23%) |
| 1973-1983; age 40-49 | 1,576 (23%) |
| 1963-1973; age 50-59 | 1,169 (17%) |
| 1953-1963; age 60-69 | 490 (7.0%) |
| 1943-1953; age 70-79 | 83 (1.2%) |
| 1943 or earlier; age 80+ | 17 (0.2%) |
| Missing | 0 (0%) |
| **Gender** |  |
| Male | 3,461 (50%) |
| Female | 3,513 (50%) |
| Other | 7 (<0.1%) |
| Missing | 11 (0.2%) |
| **Religious affiliation at age 12** |  |
| Buddhism | 5 (<0.1%) |
| Christianity | 528 (7.6%) |
| Confucianism | 1 (<0.1%) |
| Hinduism | 75 (1.1%) |
| Islam | 6,373 (91%) |
| Jainism | 1 (<0.1%) |
| No religion/Atheist/Agnostic | 2 (<0.1%) |
| Primal, Animist, or Folk religion | 1 (<0.1%) |
| Taoism | 0 (<0.1%) |
| Missing | 8 (0.1%) |
| **Race and ethnicity** |  |
| Bali | 69 (1.0%) |
| Banjar/Melayu Banjar | 320 (4.6%) |
| Batak | 165 (2.4%) |
| Betawi | 251 (3.6%) |
| Bugis | 243 (3.5%) |
| Jawa | 2,846 (41%) |
| Madura | 262 (3.7%) |
| Makasar | 91 (1.3%) |
| Minangkabau | 273 (3.9%) |
| Other | 1,262 (18%) |
| Sunda/Parahyangan | 1,172 (17%) |
| Missing | 38 (0.5%) |
| ^1^n (%) | |

***Table S8b. Regression of volunteering on childhood predictors for Indonesia***

| Variable | Category | Risk ratio | 95% CI | Global p-value |
| --- | --- | --- | --- | --- |
| Relationship with mother (Ref: Very bad/Somewhat bad) | Very good/Somewhat good | 1.14 | (0.85, 1.54) | 0.039 |
| Relationship with father (Ref: Very bad/Somewhat bad) | Very good/Somewhat good | 1.28 | (0.99, 1.66) | <.001 |
| Parent marital status (Ref: Parents married) | No, divorced | 0.98 | (0.86, 1.12) | 0.906 |
|  | Never | 1.20 | (0.81, 1.75) |  |
|  | No, one or both of them had died | 1.05 | (0.94, 1.18) |  |
| Subjective financial status of family growing up (Ref: Got by) | Lived comfortably | 1.06 | (0.99, 1.14) | 0.46 |
|  | Found it difficult | 1.07 | (0.91, 1.26) |  |
|  | Found it very difficult | 1.11 | (0.88, 1.40) |  |
| Abuse (Ref: No) | Yes | 1.17 | (1.04, 1.31) | <.001 |
| Outsider growing up (Ref: No) | Yes | 1.12 | (0.96, 1.29) | 0.037 |
| Self-rated health growing up (Ref: Good) | Excellent | 1.23 | (1.13, 1.36) | <.001 |
|  | Very good | 1.07 | (0.98, 1.17) |  |
|  | Fair | 1.00 | (0.90, 1.11) |  |
|  | Poor | 0.95 | (0.60, 1.53) |  |
| Immigration status (Ref: Born in this country) | Born in another country | 1.17 | (0.90, 1.55) | 0.067 |
| Age 12 religious service attendance (Ref: Never) | At least 1/week | 1.30 | (1.06, 1.59) | <.001 |
|  | 1-3/month | 1.25 | (1.00, 1.54) |  |
|  | <1/month | 0.93 | (0.70, 1.25) |  |
| Age group (Ref: 1998-2005; current age: 18-24) | 1993-1998; age: 25-29 | 0.96 | (0.86, 1.06) | 0.009 |
|  | 1983-1993; age: 30-39 | 0.95 | (0.87, 1.05) |  |
|  | 1973-1983; age 40-49 | 1.05 | (0.94, 1.16) |  |
|  | 1963-1973; age 50-59 | 1.11 | (0.99, 1.23) |  |
|  | 1953-1963; age 60-69 | 1.09 | (0.93, 1.30) |  |
|  | 1943-1953; age 70-79 | 0.82 | (0.52, 1.27) |  |
|  | 1943 or earlier; age 80+ | 1.02 | (0.40, 2.62) |  |
| Gender (Ref: Male) | Female | 0.86 | (0.80, 0.92) | <.001 |
|  | Other | 1.35 | (0.88, 2.07) |  |
| Religious affiliation at age 12 (Ref: Islam) | Christianity | 1.08 | (0.98, 1.19) | 0.31 |
|  | Some other religion | 1.01 | (0.79, 1.29) |  |
| Race and ethnicity plurality (Ref: Majority) | Minority | 0.92 | (0.85, 0.99) | <.001 |

***Table S8c. Sensitivity to unmeasured confounding of childhood predictors in Indonesia***

| Variable | Category | E-value for Estimate | E-value for 95% CI |
| --- | --- | --- | --- |
| Relationship with mother (Ref: Very bad/Somewhat bad) | Very good/Somewhat good | 1.54 | 1.00 |
| Relationship with father (Ref: Very bad/Somewhat bad) | Very good/Somewhat good | 1.88 | 1.00 |
| Parent marital status (Ref: Parents married) | No, divorced | 1.16 | 1.00 |
|  | Never | 1.67 | 1.00 |
|  | No, one or both of them had died | 1.29 | 1.00 |
| Subjective financial status of family growing up (Ref: Got by) | Lived comfortably | 1.32 | 1.00 |
|  | Found it difficult | 1.34 | 1.00 |
|  | Found it very difficult | 1.46 | 1.00 |
| Abuse (Ref: No) | Yes | 1.61 | 1.24 |
| Outsider growing up (Ref: No) | Yes | 1.47 | 1.00 |
| Self-rated health growing up (Ref: Good) | Excellent | 1.78 | 1.52 |
|  | Very good | 1.35 | 1.00 |
|  | Fair | 1.04 | 1.00 |
|  | Poor | 1.27 | 1.00 |
| Immigration status (Ref: Born in this country) | Born in another country | 1.64 | 1.00 |
| Age 12 religious service attendance (Ref: Never) | At least 1/week | 1.91 | 1.31 |
|  | 1-3/month | 1.79 | 1.06 |
|  | <1/month | 1.35 | 1.00 |
| Age group (Ref: 1998-2005; current age: 18-24) | 1993-1998; age: 25-29 | 1.26 | 1.00 |
|  | 1983-1993; age: 30-39 | 1.27 | 1.00 |
|  | 1973-1983; age 40-49 | 1.27 | 1.00 |
|  | 1963-1973; age 50-59 | 1.44 | 1.00 |
|  | 1953-1963; age 60-69 | 1.43 | 1.00 |
|  | 1943-1953; age 70-79 | 1.75 | 1.00 |
|  | 1943 or earlier; age 80+ | 1.19 | 1.00 |
| Gender (Ref: Male) | Female | 1.60 | 1.39 |
|  | Other | 2.04 | 1.00 |
| Religious affiliation at age 12 (Ref: Islam) | Christianity | 1.38 | 1.00 |
|  | Some other religion | 1.11 | 1.00 |
| Race and ethnicity plurality (Ref: Majority) | Minority | 1.39 | 1.08 |

***Table S9a. Nationally representative descriptive statistics for Israel***

| **Characteristic** | **N = 3,669**^1^ |
| --- | --- |
| **Relationship with mother** |  |
| Very good | 2,686 (73%) |
| Somewhat good | 793 (22%) |
| Somewhat bad | 110 (3.0%) |
| Very bad | 18 (0.5%) |
| Does not apply | 45 (1.2%) |
| Missing | 17 (0.5%) |
| **Relationship with father** |  |
| Very good | 2,290 (62%) |
| Somewhat good | 912 (25%) |
| Somewhat bad | 234 (6.4%) |
| Very bad | 37 (1.0%) |
| Does not apply | 171 (4.7%) |
| Missing | 25 (0.7%) |
| **Parent marital status** |  |
| Yes, married | 3,172 (86%) |
| No, divorced | 284 (7.8%) |
| Never married | 36 (1.0%) |
| No, one or both of them had died | 130 (3.5%) |
| Missing | 47 (1.3%) |
| **Subjective financial status of family growing up** |  |
| Lived comfortably | 923 (25%) |
| Got by | 1,822 (50%) |
| Found it difficult | 667 (18%) |
| Found it very difficult | 239 (6.5%) |
| Missing | 17 (0.5%) |
| **Abuse** |  |
| Yes | 0 (0%) |
| No | 0 (0%) |
| Missing | 3,669 (100%) |
| **Outsider growing up** |  |
| Yes | 371 (10%) |
| No | 3,228 (88%) |
| Missing | 70 (1.9%) |
| **Self-rated health growing up** |  |
| Excellent | 1,785 (49%) |
| Very good | 1,284 (35%) |
| Good | 480 (13%) |
| Fair | 105 (2.9%) |
| Poor | 6 (0.2%) |
| Missing | 8 (0.2%) |
| **Immigration status** |  |
| Born in this country | 2,796 (76%) |
| Born in another country | 868 (24%) |
| Missing | 5 (0.1%) |
| **Age 12 religious service attendance** |  |
| At least 1/week | 867 (24%) |
| 1-3/month | 435 (12%) |
| <1/month | 810 (22%) |
| Never | 1,539 (42%) |
| Missing | 17 (0.5%) |
| **Age group** |  |
| 1998-2005; current age: 18-24 | 553 (15%) |
| 1993-1998; age: 25-29 | 407 (11%) |
| 1983-1993; age: 30-39 | 666 (18%) |
| 1973-1983; age 40-49 | 616 (17%) |
| 1963-1973; age 50-59 | 542 (15%) |
| 1953-1963; age 60-69 | 469 (13%) |
| 1943-1953; age 70-79 | 336 (9.2%) |
| 1943 or earlier; age 80+ | 79 (2.2%) |
| Missing | 0 (0%) |
| **Gender** |  |
| Male | 1,791 (49%) |
| Female | 1,872 (51%) |
| Other | 0 (<0.1%) |
| Missing | 6 (0.2%) |
| **Religious affiliation at age 12** |  |
| Baha’i | 1 (<0.1%) |
| Christianity | 60 (1.6%) |
| Islam | 647 (18%) |
| Judaism | 2,873 (78%) |
| No religion/Atheist/Agnostic | 69 (1.9%) |
| Primal, Animist, or Folk religion | 3 (<0.1%) |
| Sikhism | 1 (<0.1%) |
| Some other religion | 5 (0.1%) |
| Missing | 10 (0.3%) |
| **Race and ethnicity** |  |
| Arab | 674 (18%) |
| Jewish | 2,926 (80%) |
| Other | 39 (1.1%) |
| Missing | 30 (0.8%) |
| ^1^n (%) | |

***Table S9b. Regression of volunteering on childhood predictors for Israel***

| Variable | Category | Risk ratio | 95% CI | Global p-value |
| --- | --- | --- | --- | --- |
| Relationship with mother (Ref: Very bad/Somewhat bad) | Very good/Somewhat good | 0.88 | (0.60, 1.30) | 0.219 |
| Relationship with father (Ref: Very bad/Somewhat bad) | Very good/Somewhat good | 1.23 | (0.92, 1.67) | <.001 |
| Parent marital status (Ref: Parents married) | No, divorced | 1.14 | (0.89, 1.44) | 0.123 |
|  | Never | 1.40 | (0.89, 2.22) |  |
|  | No, one or both of them had died | 0.78 | (0.45, 1.35) |  |
| Subjective financial status of family growing up (Ref: Got by) | Lived comfortably | 1.27 | (1.06, 1.51) | <.001 |
|  | Found it difficult | 0.87 | (0.70, 1.08) |  |
|  | Found it very difficult | 1.22 | (0.84, 1.79) |  |
| Outsider growing up (Ref: No) | Yes | 1.08 | (0.86, 1.36) | 0.712 |
| Self-rated health growing up (Ref: Good) | Excellent | 0.72 | (0.57, 0.91) | <.001 |
|  | Very good | 0.75 | (0.59, 0.95) |  |
|  | Fair | 0.58 | (0.35, 0.96) |  |
|  | Poor | 2.64 | (0.80, 8.70) |  |
| Immigration status (Ref: Born in this country) | Born in another country | 0.89 | (0.70, 1.12) | 0.281 |
| Age 12 religious service attendance (Ref: Never) | At least 1/week | 2.08 | (1.69, 2.54) | <.001 |
|  | 1-3/month | 1.72 | (1.33, 2.20) |  |
|  | <1/month | 1.38 | (1.12, 1.68) |  |
| Age group (Ref: 1998-2005; current age: 18-24) | 1993-1998; age: 25-29 | 0.77 | (0.60, 0.99) | <.001 |
|  | 1983-1993; age: 30-39 | 0.72 | (0.56, 0.93) |  |
|  | 1973-1983; age 40-49 | 0.84 | (0.65, 1.06) |  |
|  | 1963-1973; age 50-59 | 0.76 | (0.57, 1.02) |  |
|  | 1953-1963; age 60-69 | 0.58 | (0.41, 0.82) |  |
|  | 1943-1953; age 70-79 | 0.51 | (0.34, 0.76) |  |
|  | 1943 or earlier; age 80+ | 0.41 | (0.19, 0.89) |  |
| Gender (Ref: Male) | Female | 1.13 | (0.97, 1.32) | <.001 |
|  | Other | 7.85 | (4.08, 14.99) |  |
| Religious affiliation at age 12 (Ref: Judaism) | Islam | 0.82 | (0.34, 1.97) | 0.71 |
|  | Some other religion | 0.63 | (0.28, 1.39) |  |
| Race and ethnicity plurality (Ref: Majority) | Minority | 0.86 | (0.37, 2.00) | 0.979 |

***Table S9c. Sensitivity to unmeasured confounding of childhood predictors in Israel***

| Variable | Category | E-value for Estimate | E-value for 95% CI |
| --- | --- | --- | --- |
| Relationship with mother (Ref: Very bad/Somewhat bad) | Very good/Somewhat good | 1.53 | 1.00 |
| Relationship with father (Ref: Very bad/Somewhat bad) | Very good/Somewhat good | 1.78 | 1.00 |
| Parent marital status (Ref: Parents married) | No, divorced | 1.53 | 1.00 |
|  | Never | 2.15 | 1.00 |
|  | No, one or both of them had died | 1.88 | 1.00 |
| Subjective financial status of family growing up (Ref: Got by) | Lived comfortably | 1.85 | 1.32 |
|  | Found it difficult | 1.57 | 1.00 |
|  | Found it very difficult | 1.75 | 1.00 |
| Outsider growing up (Ref: No) | Yes | 1.37 | 1.00 |
| Self-rated health growing up (Ref: Good) | Excellent | 2.14 | 1.44 |
|  | Very good | 2.00 | 1.28 |
|  | Fair | 2.84 | 1.26 |
|  | Poor | 4.71 | 1.00 |
| Immigration status (Ref: Born in this country) | Born in another country | 1.52 | 1.00 |
| Age 12 religious service attendance (Ref: Never) | At least 1/week | 3.56 | 2.76 |
|  | 1-3/month | 2.82 | 2.00 |
|  | <1/month | 2.09 | 1.50 |
| Age group (Ref: 1998-2005; current age: 18-24) | 1993-1998; age: 25-29 | 1.93 | 1.12 |
|  | 1983-1993; age: 30-39 | 2.12 | 1.37 |
|  | 1973-1983; age 40-49 | 1.69 | 1.00 |
|  | 1963-1973; age 50-59 | 1.96 | 1.00 |
|  | 1953-1963; age 60-69 | 2.83 | 1.75 |
|  | 1943-1953; age 70-79 | 3.33 | 1.94 |
|  | 1943 or earlier; age 80+ | 4.24 | 1.49 |
| Gender (Ref: Male) | Female | 1.51 | 1.00 |
|  | Other | 15.13 | 7.63 |
| Religious affiliation at age 12 (Ref: Judaism) | Some other religion | 2.59 | 1.00 |
| Race and ethnicity plurality (Ref: Majority) | Minority | 1.58 | 1.00 |
|  | Islam | 1.73 | 1.00 |

***Table S10a. Nationally representative descriptive statistics for Japan***

| **Characteristic** | **N = 20,543**^1^ |
| --- | --- |
| **Relationship with mother** |  |
| Very good | 5,630 (27%) |
| Somewhat good | 9,461 (46%) |
| Somewhat bad | 2,750 (13%) |
| Very bad | 799 (3.9%) |
| Does not apply | 1,838 (8.9%) |
| Missing | 66 (0.3%) |
| **Relationship with father** |  |
| Very good | 4,156 (20%) |
| Somewhat good | 9,081 (44%) |
| Somewhat bad | 3,446 (17%) |
| Very bad | 1,223 (6.0%) |
| Does not apply | 2,580 (13%) |
| Missing | 57 (0.3%) |
| **Parent marital status** |  |
| Yes, married | 17,713 (86%) |
| No, divorced | 1,127 (5.5%) |
| Never married | 591 (2.9%) |
| No, one or both of them had died | 754 (3.7%) |
| Missing | 359 (1.7%) |
| **Subjective financial status of family growing up** |  |
| Lived comfortably | 8,320 (41%) |
| Got by | 8,799 (43%) |
| Found it difficult | 2,398 (12%) |
| Found it very difficult | 973 (4.7%) |
| Missing | 52 (0.3%) |
| **Abuse** |  |
| Yes | 1,482 (7.2%) |
| No | 18,964 (92%) |
| Missing | 96 (0.5%) |
| **Outsider growing up** |  |
| Yes | 1,963 (9.6%) |
| No | 17,136 (83%) |
| Missing | 1,444 (7.0%) |
| **Self-rated health growing up** |  |
| Excellent | 2,711 (13%) |
| Very good | 7,106 (35%) |
| Good | 6,689 (33%) |
| Fair | 3,199 (16%) |
| Poor | 758 (3.7%) |
| Missing | 80 (0.4%) |
| **Immigration status** |  |
| Born in this country | 19,548 (95%) |
| Born in another country | 158 (0.8%) |
| Missing | 837 (4.1%) |
| **Age 12 religious service attendance** |  |
| At least 1/week | 398 (1.9%) |
| 1-3/month | 883 (4.3%) |
| <1/month | 5,023 (24%) |
| Never | 14,117 (69%) |
| Missing | 123 (0.6%) |
| **Age group** |  |
| 1998-2005; current age: 18-24 | 1,589 (7.7%) |
| 1993-1998; age: 25-29 | 806 (3.9%) |
| 1983-1993; age: 30-39 | 2,851 (14%) |
| 1973-1983; age 40-49 | 3,363 (16%) |
| 1963-1973; age 50-59 | 3,770 (18%) |
| 1953-1963; age 60-69 | 4,118 (20%) |
| 1943-1953; age 70-79 | 3,554 (17%) |
| 1943 or earlier; age 80+ | 493 (2.4%) |
| Missing | 0 (0%) |
| **Gender** |  |
| Male | 9,847 (48%) |
| Female | 10,602 (52%) |
| Other | 28 (0.1%) |
| Missing | 66 (0.3%) |
| **Religious affiliation at age 12** |  |
| Baha’i | 7 (<0.1%) |
| Buddhism | 6,536 (32%) |
| Christianity | 343 (1.7%) |
| Confucianism | 25 (0.1%) |
| Hinduism | 4 (<0.1%) |
| Islam | 7 (<0.1%) |
| Jainism | 1 (<0.1%) |
| No religion/Atheist/Agnostic | 12,950 (63%) |
| Primal, Animist, or Folk religion | 13 (<0.1%) |
| Shinto | 382 (1.9%) |
| Some other religion | 46 (0.2%) |
| Taoism | 14 (<0.1%) |
| Missing | 215 (1.0%) |
| **Race and ethnicity** |  |
| Missing | 20,543 (100%) |
| ^1^n (%) | |

***Table S10b. Regression of volunteering on childhood predictors for Japan***

| Variable | Category | Risk ratio | 95% CI | Global p-value |
| --- | --- | --- | --- | --- |
| Relationship with mother (Ref: Very bad/Somewhat bad) | Very good/Somewhat good | 1.04 | (0.89, 1.23) | 0.395 |
| Relationship with father (Ref: Very bad/Somewhat bad) | Very good/Somewhat good | 1.20 | (1.04, 1.38) | <.001 |
| Parent marital status (Ref: Parents married) | No, divorced | 1.01 | (0.77, 1.31) | 0.004 |
|  | Never | 1.25 | (0.96, 1.62) |  |
|  | No, one or both of them had died | 1.32 | (1.01, 1.74) |  |
| Subjective financial status of family growing up (Ref: Got by) | Lived comfortably | 1.06 | (0.94, 1.20) | 0.987 |
|  | Found it difficult | 1.01 | (0.85, 1.19) |  |
|  | Found it very difficult | 0.99 | (0.76, 1.28) |  |
| Abuse (Ref: No) | Yes | 1.57 | (1.31, 1.87) | <.001 |
| Outsider growing up (Ref: No) | Yes | 1.51 | (1.27, 1.78) | <.001 |
| Self-rated health growing up (Ref: Good) | Excellent | 1.13 | (0.95, 1.33) | <.001 |
|  | Very good | 1.13 | (1.00, 1.28) |  |
|  | Fair | 0.82 | (0.69, 0.98) |  |
|  | Poor | 0.81 | (0.60, 1.10) |  |
| Immigration status (Ref: Born in this country) | Born in another country | 1.35 | (0.82, 2.22) | 0.094 |
| Age 12 religious service attendance (Ref: Never) | At least 1/week | 2.97 | (2.40, 3.69) | <.001 |
|  | 1-3/month | 2.56 | (2.15, 3.03) |  |
|  | <1/month | 1.51 | (1.33, 1.69) |  |
| Age group (Ref: 1998-2005; current age: 18-24) | 1993-1998; age: 25-29 | 0.86 | (0.63, 1.17) | <.001 |
|  | 1983-1993; age: 30-39 | 0.73 | (0.58, 0.94) |  |
|  | 1973-1983; age 40-49 | 0.66 | (0.52, 0.85) |  |
|  | 1963-1973; age 50-59 | 0.70 | (0.55, 0.88) |  |
|  | 1953-1963; age 60-69 | 0.99 | (0.80, 1.24) |  |
|  | 1943-1953; age 70-79 | 1.52 | (1.23, 1.89) |  |
|  | 1943 or earlier; age 80+ | 1.36 | (1.00, 1.85) |  |
| Gender (Ref: Male) | Female | 0.78 | (0.70, 0.86) | <.001 |
|  | Other | 0.38 | (0.09, 1.60) |  |
| Religious affiliation at age 12 (Ref: No religion/Atheist/Agnostic) | Buddhism | 1.52 | (1.36, 1.71) | <.001 |
|  | Some other religion | 1.90 | (1.57, 2.31) |  |

***Table S10c. Sensitivity to unmeasured confounding of childhood predictors in Japan***

| Variable | Category | E-value for Estimate | E-value for 95% CI |
| --- | --- | --- | --- |
| Relationship with mother (Ref: Very bad/Somewhat bad) | Very good/Somewhat good | 1.26 | 1.00 |
| Relationship with father (Ref: Very bad/Somewhat bad) | Very good/Somewhat good | 1.68 | 1.23 |
| Parent marital status (Ref: Parents married) | No, divorced | 1.08 | 1.00 |
|  | Never | 1.80 | 1.00 |
|  | No, one or both of them had died | 1.98 | 1.08 |
| Subjective financial status of family growing up (Ref: Got by) | Lived comfortably | 1.33 | 1.00 |
|  | Found it difficult | 1.11 | 1.00 |
|  | Found it very difficult | 1.12 | 1.00 |
| Abuse (Ref: No) | Yes | 2.51 | 1.95 |
| Outsider growing up (Ref: No) | Yes | 2.38 | 1.86 |
| Self-rated health growing up (Ref: Good) | Excellent | 1.50 | 1.00 |
|  | Very good | 1.52 | 1.00 |
|  | Fair | 1.73 | 1.17 |
|  | Poor | 1.76 | 1.00 |
| Immigration status (Ref: Born in this country) | Born in another country | 2.04 | 1.00 |
| Age 12 religious service attendance (Ref: Never) | At least 1/week | 5.40 | 4.23 |
|  | 1-3/month | 4.54 | 3.72 |
|  | <1/month | 2.37 | 1.99 |
| Age group (Ref: 1998-2005; current age: 18-24) | 1993-1998; age: 25-29 | 1.60 | 1.00 |
|  | 1983-1993; age: 30-39 | 2.05 | 1.32 |
|  | 1973-1983; age 40-49 | 2.37 | 1.64 |
|  | 1963-1973; age 50-59 | 2.23 | 1.52 |
|  | 1953-1963; age 60-69 | 1.10 | 1.00 |
|  | 1943-1953; age 70-79 | 2.42 | 1.76 |
|  | 1943 or earlier; age 80+ | 2.07 | 1.06 |
| Gender (Ref: Male) | Female | 1.89 | 1.60 |
|  | Other | 4.73 | 1.00 |
| Religious affiliation at age 12 (Ref: No religion/Atheist/Agnostic) | Some other religion | 3.22 | 2.51 |
|  | Buddhism | 2.43 | 2.07 |

***Table S11a. Nationally representative descriptive statistics for Kenya***

| **Characteristic** | **N = 11,389**^1^ |
| --- | --- |
| **Relationship with mother** |  |
| Very good | 9,418 (83%) |
| Somewhat good | 1,435 (13%) |
| Somewhat bad | 130 (1.1%) |
| Very bad | 100 (0.9%) |
| Does not apply | 240 (2.1%) |
| Missing | 66 (0.6%) |
| **Relationship with father** |  |
| Very good | 7,958 (70%) |
| Somewhat good | 1,896 (17%) |
| Somewhat bad | 216 (1.9%) |
| Very bad | 220 (1.9%) |
| Does not apply | 967 (8.5%) |
| Missing | 132 (1.2%) |
| **Parent marital status** |  |
| Yes, married | 9,238 (81%) |
| No, divorced | 697 (6.1%) |
| Never married | 681 (6.0%) |
| No, one or both of them had died | 471 (4.1%) |
| Missing | 301 (2.6%) |
| **Subjective financial status of family growing up** |  |
| Lived comfortably | 3,026 (27%) |
| Got by | 3,279 (29%) |
| Found it difficult | 4,071 (36%) |
| Found it very difficult | 994 (8.7%) |
| Missing | 19 (0.2%) |
| **Abuse** |  |
| Yes | 1,300 (11%) |
| No | 10,039 (88%) |
| Missing | 49 (0.4%) |
| **Outsider growing up** |  |
| Yes | 1,223 (11%) |
| No | 10,114 (89%) |
| Missing | 52 (0.5%) |
| **Self-rated health growing up** |  |
| Excellent | 4,449 (39%) |
| Very good | 2,598 (23%) |
| Good | 2,582 (23%) |
| Fair | 1,384 (12%) |
| Poor | 349 (3.1%) |
| Missing | 26 (0.2%) |
| **Immigration status** |  |
| Born in this country | 11,270 (99%) |
| Born in another country | 117 (1.0%) |
| Missing | 2 (<0.1%) |
| **Age 12 religious service attendance** |  |
| At least 1/week | 9,189 (81%) |
| 1-3/month | 1,687 (15%) |
| <1/month | 236 (2.1%) |
| Never | 198 (1.7%) |
| Missing | 79 (0.7%) |
| **Age group** |  |
| 1998-2005; current age: 18-24 | 2,868 (25%) |
| 1993-1998; age: 25-29 | 2,035 (18%) |
| 1983-1993; age: 30-39 | 2,564 (23%) |
| 1973-1983; age 40-49 | 1,708 (15%) |
| 1963-1973; age 50-59 | 1,072 (9.4%) |
| 1953-1963; age 60-69 | 710 (6.2%) |
| 1943-1953; age 70-79 | 360 (3.2%) |
| 1943 or earlier; age 80+ | 67 (0.6%) |
| Missing | 5 (<0.1%) |
| **Gender** |  |
| Male | 5,567 (49%) |
| Female | 5,813 (51%) |
| Other | 2 (<0.1%) |
| Missing | 7 (<0.1%) |
| **Religious affiliation at age 12** |  |
| Baha’i | 3 (<0.1%) |
| Buddhism | 5 (<0.1%) |
| Christianity | 10,369 (91%) |
| Islam | 916 (8.0%) |
| Jainism | 1 (<0.1%) |
| Judaism | 6 (<0.1%) |
| No religion/Atheist/Agnostic | 67 (0.6%) |
| Primal, Animist, or Folk religion | 13 (0.1%) |
| Sikhism | 0 (<0.1%) |
| Some other religion | 0 (<0.1%) |
| Missing | 9 (<0.1%) |
| **Race and ethnicity** |  |
| Embu | 197 (1.7%) |
| Kalenjin | 1,377 (12%) |
| Kamba | 1,299 (11%) |
| Kenyan Somali/Somali | 396 (3.5%) |
| Kikuyu | 2,119 (19%) |
| Kisii | 789 (6.9%) |
| Luhya | 1,943 (17%) |
| Luo | 1,120 (9.8%) |
| Maasai | 237 (2.1%) |
| Meru | 630 (5.5%) |
| Miji Kenda tribes | 708 (6.2%) |
| Other | 548 (4.8%) |
| Missing | 27 (0.2%) |
| ^1^n (%) | |

***Table S11b. Regression of volunteering on childhood predictors for Kenya***

| Variable | Category | Risk ratio | 95% CI | Global p-value |
| --- | --- | --- | --- | --- |
| Relationship with mother (Ref: Very bad/Somewhat bad) | Very good/Somewhat good | 1.05 | (0.88, 1.26) | 0.336 |
| Relationship with father (Ref: Very bad/Somewhat bad) | Very good/Somewhat good | 1.03 | (0.91, 1.15) | 0.612 |
| Parent marital status (Ref: Parents married) | No, divorced | 1.03 | (0.91, 1.16) | 0.47 |
|  | Never | 1.04 | (0.92, 1.17) |  |
|  | No, one or both of them had died | 0.90 | (0.78, 1.04) |  |
| Subjective financial status of family growing up (Ref: Got by) | Lived comfortably | 0.99 | (0.91, 1.07) | <.001 |
|  | Found it difficult | 0.94 | (0.88, 1.01) |  |
|  | Found it very difficult | 1.12 | (1.00, 1.25) |  |
| Abuse (Ref: No) | Yes | 1.08 | (0.98, 1.19) | 0.012 |
| Outsider growing up (Ref: No) | Yes | 1.11 | (1.01, 1.22) | <.001 |
| Self-rated health growing up (Ref: Good) | Excellent | 1.04 | (0.97, 1.12) | <.001 |
|  | Very good | 1.03 | (0.95, 1.12) |  |
|  | Fair | 0.82 | (0.72, 0.92) |  |
|  | Poor | 0.92 | (0.76, 1.11) |  |
| Immigration status (Ref: Born in this country) | Born in another country | 0.76 | (0.53, 1.10) | 0.037 |
| Age 12 religious service attendance (Ref: Never) | At least 1/week | 1.26 | (0.94, 1.68) | 0.001 |
|  | 1-3/month | 1.14 | (0.84, 1.54) |  |
|  | <1/month | 1.21 | (0.86, 1.69) |  |
| Age group (Ref: 1998-2005; current age: 18-24) | 1993-1998; age: 25-29 | 1.09 | (1.02, 1.18) | <.001 |
|  | 1983-1993; age: 30-39 | 1.07 | (0.99, 1.15) |  |
|  | 1973-1983; age 40-49 | 1.13 | (1.02, 1.25) |  |
|  | 1963-1973; age 50-59 | 1.27 | (1.14, 1.42) |  |
|  | 1953-1963; age 60-69 | 1.09 | (0.94, 1.28) |  |
|  | 1943-1953; age 70-79 | 1.08 | (0.87, 1.35) |  |
|  | 1943 or earlier; age 80+ | 0.98 | (0.55, 1.75) |  |
| Gender (Ref: Male) | Female | 0.92 | (0.86, 0.98) | <.001 |
|  | Other | 0.63 | (0.09, 4.47) |  |
| Religious affiliation at age 12 (Ref: Christianity) | Islam | 0.90 | (0.75, 1.08) | 0.772 |
|  | Some other religion | 0.97 | (0.63, 1.49) |  |
| Race and ethnicity plurality (Ref: Majority) | Minority | 1.38 | (1.25, 1.52) | <.001 |

***Table S11c. Sensitivity to unmeasured confounding of childhood predictors in Kenya***

| Variable | Category | E-value for Estimate | E-value for 95% CI |
| --- | --- | --- | --- |
| Relationship with mother (Ref: Very bad/Somewhat bad) | Very good/Somewhat good | 1.28 | 1.00 |
| Relationship with father (Ref: Very bad/Somewhat bad) | Very good/Somewhat good | 1.19 | 1.00 |
| Parent marital status (Ref: Parents married) | No, divorced | 1.19 | 1.00 |
|  | Never | 1.24 | 1.00 |
|  | No, one or both of them had died | 1.46 | 1.00 |
| Subjective financial status of family growing up (Ref: Got by) | Lived comfortably | 1.12 | 1.00 |
|  | Found it difficult | 1.31 | 1.00 |
|  | Found it very difficult | 1.49 | 1.06 |
| Abuse (Ref: No) | Yes | 1.38 | 1.00 |
| Outsider growing up (Ref: No) | Yes | 1.45 | 1.10 |
| Self-rated health growing up (Ref: Good) | Excellent | 1.24 | 1.00 |
|  | Very good | 1.20 | 1.00 |
|  | Fair | 1.75 | 1.40 |
|  | Poor | 1.40 | 1.00 |
| Immigration status (Ref: Born in this country) | Born in another country | 1.95 | 1.00 |
| Age 12 religious service attendance (Ref: Never) | At least 1/week | 1.82 | 1.00 |
|  | 1-3/month | 1.53 | 1.00 |
|  | <1/month | 1.71 | 1.00 |
| Age group (Ref: 1998-2005; current age: 18-24) | 1993-1998; age: 25-29 | 1.42 | 1.16 |
|  | 1983-1993; age: 30-39 | 1.34 | 1.00 |
|  | 1973-1983; age 40-49 | 1.52 | 1.18 |
|  | 1963-1973; age 50-59 | 1.86 | 1.53 |
|  | 1953-1963; age 60-69 | 1.42 | 1.00 |
|  | 1943-1953; age 70-79 | 1.39 | 1.00 |
|  | 1943 or earlier; age 80+ | 1.14 | 1.00 |
| Gender (Ref: Male) | Female | 1.39 | 1.16 |
|  | Other | 2.55 | 1.00 |
| Religious affiliation at age 12 (Ref: Christianity) | Some other religion | 1.19 | 1.00 |
| Race and ethnicity plurality (Ref: Majority) | Minority | 2.10 | 1.80 |
|  | Islam | 1.46 | 1.00 |

***Table S12a. Nationally representative descriptive statistics for Mexico***

| **Characteristic** | **N = 5,776**^1^ |
| --- | --- |
| **Relationship with mother** |  |
| Very good | 3,912 (68%) |
| Somewhat good | 1,340 (23%) |
| Somewhat bad | 177 (3.1%) |
| Very bad | 90 (1.6%) |
| Does not apply | 177 (3.1%) |
| Missing | 80 (1.4%) |
| **Relationship with father** |  |
| Very good | 3,089 (53%) |
| Somewhat good | 1,556 (27%) |
| Somewhat bad | 335 (5.8%) |
| Very bad | 267 (4.6%) |
| Does not apply | 470 (8.1%) |
| Missing | 60 (1.0%) |
| **Parent marital status** |  |
| Yes, married | 3,999 (69%) |
| No, divorced | 341 (5.9%) |
| Never married | 827 (14%) |
| No, one or both of them had died | 176 (3.0%) |
| Missing | 432 (7.5%) |
| **Subjective financial status of family growing up** |  |
| Lived comfortably | 1,775 (31%) |
| Got by | 1,872 (32%) |
| Found it difficult | 1,712 (30%) |
| Found it very difficult | 369 (6.4%) |
| Missing | 48 (0.8%) |
| **Abuse** |  |
| Yes | 905 (16%) |
| No | 4,604 (80%) |
| Missing | 267 (4.6%) |
| **Outsider growing up** |  |
| Yes | 772 (13%) |
| No | 4,897 (85%) |
| Missing | 107 (1.9%) |
| **Self-rated health growing up** |  |
| Excellent | 1,860 (32%) |
| Very good | 1,350 (23%) |
| Good | 1,677 (29%) |
| Fair | 743 (13%) |
| Poor | 133 (2.3%) |
| Missing | 14 (0.2%) |
| **Immigration status** |  |
| Born in this country | 5,517 (96%) |
| Born in another country | 108 (1.9%) |
| Missing | 151 (2.6%) |
| **Age 12 religious service attendance** |  |
| At least 1/week | 2,514 (44%) |
| 1-3/month | 1,162 (20%) |
| <1/month | 1,087 (19%) |
| Never | 944 (16%) |
| Missing | 69 (1.2%) |
| **Age group** |  |
| 1998-2005; current age: 18-24 | 986 (17%) |
| 1993-1998; age: 25-29 | 623 (11%) |
| 1983-1993; age: 30-39 | 1,312 (23%) |
| 1973-1983; age 40-49 | 1,027 (18%) |
| 1963-1973; age 50-59 | 873 (15%) |
| 1953-1963; age 60-69 | 611 (11%) |
| 1943-1953; age 70-79 | 277 (4.8%) |
| 1943 or earlier; age 80+ | 68 (1.2%) |
| Missing | 0 (0%) |
| **Gender** |  |
| Male | 2,755 (48%) |
| Female | 2,997 (52%) |
| Other | 3 (<0.1%) |
| Missing | 21 (0.4%) |
| **Religious affiliation at age 12** |  |
| Baha’i | 1 (<0.1%) |
| Buddhism | 1 (<0.1%) |
| Christianity | 5,337 (92%) |
| Hinduism | 1 (<0.1%) |
| Islam | 6 (<0.1%) |
| Judaism | 8 (0.1%) |
| No religion/Atheist/Agnostic | 328 (5.7%) |
| Primal, Animist, or Folk religion | 2 (<0.1%) |
| Shinto | 2 (<0.1%) |
| Sikhism | 4 (<0.1%) |
| Some other religion | 7 (0.1%) |
| Taoism | 5 (<0.1%) |
| Missing | 74 (1.3%) |
| **Race and ethnicity** |  |
| Black | 108 (1.9%) |
| Indigenous | 594 (10%) |
| Mestizo | 2,762 (48%) |
| Mulatto | 63 (1.1%) |
| Other | 339 (5.9%) |
| White | 1,116 (19%) |
| Missing | 794 (14%) |
| ^1^n (%) | |

***Table S12b. Regression of volunteering on childhood predictors for Mexico***

| Variable | Category | Risk ratio | 95% CI | Global p-value |
| --- | --- | --- | --- | --- |
| Relationship with mother (Ref: Very bad/Somewhat bad) | Very good/Somewhat good | 1.07 | (0.78, 1.48) | 0.574 |
| Relationship with father (Ref: Very bad/Somewhat bad) | Very good/Somewhat good | 1.28 | (1.02, 1.61) | <.001 |
| Parent marital status (Ref: Parents married) | No, divorced | 1.11 | (0.83, 1.46) | 0.997 |
|  | Never | 1.05 | (0.86, 1.29) |  |
|  | No, one or both of them had died | 1.00 | (0.67, 1.50) |  |
| Subjective financial status of family growing up (Ref: Got by) | Lived comfortably | 0.95 | (0.80, 1.12) | 0.021 |
|  | Found it difficult | 0.90 | (0.76, 1.08) |  |
|  | Found it very difficult | 1.23 | (0.95, 1.60) |  |
| Abuse (Ref: No) | Yes | 1.00 | (0.83, 1.21) | 1 |
| Outsider growing up (Ref: No) | Yes | 1.27 | (1.06, 1.52) | <.001 |
| Self-rated health growing up (Ref: Good) | Excellent | 1.26 | (1.05, 1.52) | <.001 |
|  | Very good | 1.19 | (0.99, 1.43) |  |
|  | Fair | 1.35 | (1.08, 1.68) |  |
|  | Poor | 1.52 | (1.03, 2.25) |  |
| Immigration status (Ref: Born in this country) | Born in another country | 1.19 | (0.76, 1.85) | 0.614 |
| Age 12 religious service attendance (Ref: Never) | At least 1/week | 1.55 | (1.23, 1.96) | <.001 |
|  | 1-3/month | 1.43 | (1.12, 1.84) |  |
|  | <1/month | 1.12 | (0.86, 1.45) |  |
| Age group (Ref: 1998-2005; current age: 18-24) | 1993-1998; age: 25-29 | 0.99 | (0.77, 1.28) | 0.414 |
|  | 1983-1993; age: 30-39 | 0.98 | (0.79, 1.21) |  |
|  | 1973-1983; age 40-49 | 1.13 | (0.90, 1.42) |  |
|  | 1963-1973; age 50-59 | 0.93 | (0.73, 1.20) |  |
|  | 1953-1963; age 60-69 | 1.04 | (0.80, 1.37) |  |
|  | 1943-1953; age 70-79 | 0.84 | (0.58, 1.24) |  |
|  | 1943 or earlier; age 80+ | 0.51 | (0.22, 1.17) |  |
| Gender (Ref: Male) | Female | 0.79 | (0.69, 0.90) | <.001 |
|  | Other | 0.65 | (0.10, 4.25) |  |
| Religious affiliation at age 12 (Ref: No religion/Atheist/Agnostic) | Christianity | 0.90 | (0.67, 1.18) | 0.45 |
|  | Some other religion | 1.28 | (0.67, 2.48) |  |
| Race and ethnicity plurality (Ref: Majority) | Minority | 1.12 | (0.98, 1.28) | 0.011 |

***Table S12c. Sensitivity to unmeasured confounding of childhood predictors in Mexico***

| Variable | Category | E-value for Estimate | E-value for 95% CI |
| --- | --- | --- | --- |
| Relationship with mother (Ref: Very bad/Somewhat bad) | Very good/Somewhat good | 1.35 | 1.00 |
| Relationship with father (Ref: Very bad/Somewhat bad) | Very good/Somewhat good | 1.88 | 1.17 |
| Parent marital status (Ref: Parents married) | No, divorced | 1.44 | 1.00 |
|  | Never | 1.30 | 1.00 |
|  | No, one or both of them had died | 1.02 | 1.00 |
| Subjective financial status of family growing up (Ref: Got by) | Lived comfortably | 1.29 | 1.00 |
|  | Found it difficult | 1.45 | 1.00 |
|  | Found it very difficult | 1.76 | 1.00 |
| Abuse (Ref: No) | Yes | 1.08 | 1.00 |
| Outsider growing up (Ref: No) | Yes | 1.86 | 1.33 |
| Self-rated health growing up (Ref: Good) | Excellent | 1.84 | 1.29 |
|  | Very good | 1.66 | 1.00 |
|  | Fair | 2.03 | 1.38 |
|  | Poor | 2.41 | 1.19 |
| Immigration status (Ref: Born in this country) | Born in another country | 1.66 | 1.00 |
| Age 12 religious service attendance (Ref: Never) | At least 1/week | 2.48 | 1.77 |
|  | 1-3/month | 2.22 | 1.49 |
|  | <1/month | 1.48 | 1.00 |
| Age group (Ref: 1998-2005; current age: 18-24) | 1993-1998; age: 25-29 | 1.09 | 1.00 |
|  | 1983-1993; age: 30-39 | 1.19 | 1.00 |
|  | 1973-1983; age 40-49 | 1.52 | 1.00 |
|  | 1963-1973; age 50-59 | 1.35 | 1.00 |
|  | 1953-1963; age 60-69 | 1.26 | 1.00 |
|  | 1943-1953; age 70-79 | 1.65 | 1.00 |
|  | 1943 or earlier; age 80+ | 3.32 | 1.00 |
| Gender (Ref: Male) | Female | 1.85 | 1.46 |
|  | Other | 2.46 | 1.00 |
| Religious affiliation at age 12 (Ref: No religion/Atheist/Agnostic) | Christianity | 1.49 | 1.00 |
|  | Some other religion | 1.89 | 1.00 |
| Race and ethnicity plurality (Ref: Majority) | Minority | 1.49 | 1.00 |

***Table S13a. Nationally representative descriptive statistics for Nigeria***

| **Characteristic** | **N = 6,827**^1^ |
| --- | --- |
| **Relationship with mother** |  |
| Very good | 5,986 (88%) |
| Somewhat good | 648 (9.5%) |
| Somewhat bad | 62 (0.9%) |
| Very bad | 18 (0.3%) |
| Does not apply | 104 (1.5%) |
| Missing | 9 (0.1%) |
| **Relationship with father** |  |
| Very good | 5,578 (82%) |
| Somewhat good | 924 (14%) |
| Somewhat bad | 76 (1.1%) |
| Very bad | 43 (0.6%) |
| Does not apply | 177 (2.6%) |
| Missing | 29 (0.4%) |
| **Parent marital status** |  |
| Yes, married | 5,568 (82%) |
| No, divorced | 307 (4.5%) |
| Never married | 335 (4.9%) |
| No, one or both of them had died | 462 (6.8%) |
| Missing | 154 (2.3%) |
| **Subjective financial status of family growing up** |  |
| Lived comfortably | 2,192 (32%) |
| Got by | 2,381 (35%) |
| Found it difficult | 1,661 (24%) |
| Found it very difficult | 563 (8.3%) |
| Missing | 29 (0.4%) |
| **Abuse** |  |
| Yes | 880 (13%) |
| No | 5,851 (86%) |
| Missing | 96 (1.4%) |
| **Outsider growing up** |  |
| Yes | 669 (9.8%) |
| No | 6,059 (89%) |
| Missing | 99 (1.5%) |
| **Self-rated health growing up** |  |
| Excellent | 2,644 (39%) |
| Very good | 2,613 (38%) |
| Good | 1,152 (17%) |
| Fair | 306 (4.5%) |
| Poor | 98 (1.4%) |
| Missing | 14 (0.2%) |
| **Immigration status** |  |
| Born in this country | 6,779 (99%) |
| Born in another country | 47 (0.7%) |
| Missing | 1 (<0.1%) |
| **Age 12 religious service attendance** |  |
| At least 1/week | 5,907 (87%) |
| 1-3/month | 600 (8.8%) |
| <1/month | 136 (2.0%) |
| Never | 138 (2.0%) |
| Missing | 45 (0.7%) |
| **Age group** |  |
| 1998-2005; current age: 18-24 | 1,533 (22%) |
| 1993-1998; age: 25-29 | 1,193 (17%) |
| 1983-1993; age: 30-39 | 1,943 (28%) |
| 1973-1983; age 40-49 | 1,059 (16%) |
| 1963-1973; age 50-59 | 619 (9.1%) |
| 1953-1963; age 60-69 | 296 (4.3%) |
| 1943-1953; age 70-79 | 133 (2.0%) |
| 1943 or earlier; age 80+ | 50 (0.7%) |
| Missing | 0 (0%) |
| **Gender** |  |
| Male | 3,371 (49%) |
| Female | 3,456 (51%) |
| Other | 0 (<0.1%) |
| Missing | 0 (0%) |
| **Religious affiliation at age 12** |  |
| Buddhism | 0 (<0.1%) |
| Christianity | 3,463 (51%) |
| Confucianism | 0 (<0.1%) |
| Islam | 3,314 (49%) |
| No religion/Atheist/Agnostic | 19 (0.3%) |
| Primal, Animist, or Folk religion | 17 (0.3%) |
| Missing | 14 (0.2%) |
| **Race and ethnicity** |  |
| Edo | 116 (1.7%) |
| Efik | 48 (0.7%) |
| Fulani | 266 (3.9%) |
| Hausa | 2,342 (34%) |
| Ibibio | 180 (2.6%) |
| Idoma | 61 (0.9%) |
| Igala | 77 (1.1%) |
| Igbo (Ibo) | 1,111 (16%) |
| Ijaw | 110 (1.6%) |
| Kanuri | 31 (0.5%) |
| Other | 1,014 (15%) |
| Tiv | 198 (2.9%) |
| Urhobo | 38 (0.6%) |
| Yoruba | 1,230 (18%) |
| Missing | 4 (<0.1%) |
| ^1^n (%) | |

***Table S13b. Regression of volunteering on childhood predictors for Nigeria***

| Variable | Category | Risk ratio | 95% CI | Global p-value |
| --- | --- | --- | --- | --- |
| Relationship with mother (Ref: Very bad/Somewhat bad) | Very good/Somewhat good | 0.95 | (0.74, 1.22) | 0.701 |
| Relationship with father (Ref: Very bad/Somewhat bad) | Very good/Somewhat good | 1.11 | (0.90, 1.36) | 0.015 |
| Parent marital status (Ref: Parents married) | No, divorced | 0.95 | (0.81, 1.12) | 0.778 |
|  | Never | 0.95 | (0.82, 1.10) |  |
|  | No, one or both of them had died | 1.06 | (0.93, 1.20) |  |
| Subjective financial status of family growing up (Ref: Got by) | Lived comfortably | 1.21 | (1.10, 1.32) | <.001 |
|  | Found it difficult | 1.02 | (0.92, 1.14) |  |
|  | Found it very difficult | 0.80 | (0.66, 0.97) |  |
| Abuse (Ref: No) | Yes | 0.97 | (0.88, 1.07) | 0.807 |
| Outsider growing up (Ref: No) | Yes | 1.02 | (0.90, 1.16) | 0.963 |
| Self-rated health growing up (Ref: Good) | Excellent | 0.91 | (0.83, 1.01) | 0.43 |
|  | Very good | 0.94 | (0.86, 1.04) |  |
|  | Fair | 1.07 | (0.88, 1.30) |  |
|  | Poor | 0.96 | (0.69, 1.34) |  |
| Immigration status (Ref: Born in this country) | Born in another country | 0.67 | (0.33, 1.36) | 0.216 |
| Age 12 religious service attendance (Ref: Never) | At least 1/week | 1.92 | (1.07, 3.40) | <.001 |
|  | 1-3/month | 1.73 | (0.94, 3.19) |  |
|  | <1/month | 1.58 | (0.82, 3.03) |  |
| Age group (Ref: 1998-2005; current age: 18-24) | 1993-1998; age: 25-29 | 1.15 | (1.03, 1.28) | <.001 |
|  | 1983-1993; age: 30-39 | 1.20 | (1.08, 1.32) |  |
|  | 1973-1983; age 40-49 | 1.23 | (1.10, 1.40) |  |
|  | 1963-1973; age 50-59 | 1.27 | (1.08, 1.48) |  |
|  | 1953-1963; age 60-69 | 1.12 | (0.90, 1.40) |  |
|  | 1943-1953; age 70-79 | 1.00 | (0.71, 1.42) |  |
|  | 1943 or earlier; age 80+ | 0.76 | (0.31, 1.88) |  |
| Gender (Ref: Male) | Female | 0.76 | (0.70, 0.82) | <.001 |
|  | Other | 1.68 | (1.47, 1.92) |  |
| Religious affiliation at age 12 (Ref: Christianity) | Islam | 0.89 | (0.79, 1.00) | 0.003 |
|  | Some other religion | 1.26 | (0.83, 1.92) |  |
| Race and ethnicity plurality (Ref: Majority) | Minority | 1.00 | (0.87, 1.14) | 1 |

***Table S13c. Sensitivity to unmeasured confounding of childhood predictors in Nigeria***

| Variable | Category | E-value for Estimate | E-value for 95% CI |
| --- | --- | --- | --- |
| Relationship with mother (Ref: Very bad/Somewhat bad) | Very good/Somewhat good | 1.28 | 1.00 |
| Relationship with father (Ref: Very bad/Somewhat bad) | Very good/Somewhat good | 1.45 | 1.00 |
| Parent marital status (Ref: Parents married) | No, divorced | 1.29 | 1.00 |
|  | Never | 1.29 | 1.00 |
|  | No, one or both of them had died | 1.30 | 1.00 |
| Subjective financial status of family growing up (Ref: Got by) | Lived comfortably | 1.70 | 1.42 |
|  | Found it difficult | 1.18 | 1.00 |
|  | Found it very difficult | 1.81 | 1.21 |
| Abuse (Ref: No) | Yes | 1.21 | 1.00 |
| Outsider growing up (Ref: No) | Yes | 1.18 | 1.00 |
| Self-rated health growing up (Ref: Good) | Excellent | 1.41 | 1.00 |
|  | Very good | 1.31 | 1.00 |
|  | Fair | 1.34 | 1.00 |
|  | Poor | 1.24 | 1.00 |
| Immigration status (Ref: Born in this country) | Born in another country | 2.35 | 1.00 |
| Age 12 religious service attendance (Ref: Never) | At least 1/week | 3.22 | 1.34 |
|  | 1-3/month | 2.86 | 1.00 |
|  | <1/month | 2.54 | 1.00 |
| Age group (Ref: 1998-2005; current age: 18-24) | 1993-1998; age: 25-29 | 1.57 | 1.22 |
|  | 1983-1993; age: 30-39 | 1.67 | 1.36 |
|  | 1973-1983; age 40-49 | 1.78 | 1.42 |
|  | 1963-1973; age 50-59 | 1.84 | 1.39 |
|  | 1953-1963; age 60-69 | 1.48 | 1.00 |
|  | 1943-1953; age 70-79 | 1.06 | 1.00 |
|  | 1943 or earlier; age 80+ | 1.94 | 1.00 |
| Gender (Ref: Male) | Female | 1.97 | 1.73 |
|  | Other | 2.75 | 2.30 |
| Religious affiliation at age 12 (Ref: Christianity) | Some other religion | 1.84 | 1.00 |
| Race and ethnicity plurality (Ref: Majority) | Minority | 1.05 | 1.00 |
|  | Islam | 1.50 | 1.00 |

***Table S14a. Nationally representative descriptive statistics for Philippines***

| **Characteristic** | **N = 5,292**^1^ |
| --- | --- |
| **Relationship with mother** |  |
| Very good | 3,333 (63%) |
| Somewhat good | 1,703 (32%) |
| Somewhat bad | 124 (2.3%) |
| Very bad | 39 (0.7%) |
| Does not apply | 59 (1.1%) |
| Missing | 35 (0.7%) |
| **Relationship with father** |  |
| Very good | 3,443 (65%) |
| Somewhat good | 1,429 (27%) |
| Somewhat bad | 159 (3.0%) |
| Very bad | 58 (1.1%) |
| Does not apply | 108 (2.0%) |
| Missing | 95 (1.8%) |
| **Parent marital status** |  |
| Yes, married | 4,575 (86%) |
| No, divorced | 64 (1.2%) |
| Never married | 517 (9.8%) |
| No, one or both of them had died | 51 (1.0%) |
| Missing | 85 (1.6%) |
| **Subjective financial status of family growing up** |  |
| Lived comfortably | 937 (18%) |
| Got by | 3,006 (57%) |
| Found it difficult | 1,055 (20%) |
| Found it very difficult | 291 (5.5%) |
| Missing | 3 (<0.1%) |
| **Abuse** |  |
| Yes | 420 (7.9%) |
| No | 4,837 (91%) |
| Missing | 35 (0.7%) |
| **Outsider growing up** |  |
| Yes | 395 (7.5%) |
| No | 4,884 (92%) |
| Missing | 13 (0.2%) |
| **Self-rated health growing up** |  |
| Excellent | 1,041 (20%) |
| Very good | 559 (11%) |
| Good | 2,174 (41%) |
| Fair | 1,246 (24%) |
| Poor | 272 (5.1%) |
| Missing | 0 (<0.1%) |
| **Immigration status** |  |
| Born in this country | 5,284 (100%) |
| Born in another country | 8 (0.1%) |
| Missing | 0 (0%) |
| **Age 12 religious service attendance** |  |
| At least 1/week | 2,453 (46%) |
| 1-3/month | 1,699 (32%) |
| <1/month | 892 (17%) |
| Never | 201 (3.8%) |
| Missing | 47 (0.9%) |
| **Age group** |  |
| 1998-2005; current age: 18-24 | 1,073 (20%) |
| 1993-1998; age: 25-29 | 695 (13%) |
| 1983-1993; age: 30-39 | 1,160 (22%) |
| 1973-1983; age 40-49 | 972 (18%) |
| 1963-1973; age 50-59 | 732 (14%) |
| 1953-1963; age 60-69 | 495 (9.4%) |
| 1943-1953; age 70-79 | 143 (2.7%) |
| 1943 or earlier; age 80+ | 23 (0.4%) |
| Missing | 0 (0%) |
| **Gender** |  |
| Male | 2,625 (50%) |
| Female | 2,643 (50%) |
| Other | 13 (0.2%) |
| Missing | 11 (0.2%) |
| **Religious affiliation at age 12** |  |
| Baha’i | 1 (<0.1%) |
| Buddhism | 1 (<0.1%) |
| Christianity | 4,968 (94%) |
| Islam | 276 (5.2%) |
| No religion/Atheist/Agnostic | 9 (0.2%) |
| Primal, Animist, or Folk religion | 14 (0.3%) |
| Sikhism | 4 (<0.1%) |
| Some other religion | 9 (0.2%) |
| Missing | 11 (0.2%) |
| **Race and ethnicity** |  |
| Aeta | 1 (<0.1%) |
| Badjao | 2 (<0.1%) |
| Bicolano/Bikolano | 300 (5.7%) |
| Cebuano | 656 (12%) |
| Chinese-Filipino | 3 (<0.1%) |
| Igorot | 42 (0.8%) |
| Ilocano/Ilokano | 429 (8.1%) |
| Ilonggo/Hiligaynon | 428 (8.1%) |
| Kapampangan | 107 (2.0%) |
| Maguindanaoan | 84 (1.6%) |
| Mangyan | 2 (<0.1%) |
| Maranao | 39 (0.7%) |
| Masbateno | 54 (1.0%) |
| Other | 244 (4.6%) |
| Pangasinense | 107 (2.0%) |
| Tagalog | 1,691 (32%) |
| Tausug | 94 (1.8%) |
| Visayan/Bisaya | 739 (14%) |
| Waray | 216 (4.1%) |
| Zamboangueno | 51 (1.0%) |
| Missing | 3 (<0.1%) |
| ^1^n (%) | |

***Table S14b. Regression of volunteering on childhood predictors for Philippines***

| Variable | Category | Risk ratio | 95% CI | Global p-value |
| --- | --- | --- | --- | --- |
| Relationship with mother (Ref: Very bad/Somewhat bad) | Very good/Somewhat good | 1.27 | (0.92, 1.75) | <.001 |
| Relationship with father (Ref: Very bad/Somewhat bad) | Very good/Somewhat good | 0.90 | (0.70, 1.18) | 0.078 |
| Parent marital status (Ref: Parents married) | No, divorced | 1.25 | (0.79, 1.95) | 0.797 |
|  | Never | 1.11 | (0.91, 1.34) |  |
|  | No, one or both of them had died | 0.93 | (0.53, 1.63) |  |
| Subjective financial status of family growing up (Ref: Got by) | Lived comfortably | 1.26 | (1.08, 1.46) | <.001 |
|  | Found it difficult | 0.98 | (0.85, 1.15) |  |
|  | Found it very difficult | 0.96 | (0.75, 1.23) |  |
| Abuse (Ref: No) | Yes | 1.05 | (0.86, 1.29) | 0.873 |
| Outsider growing up (Ref: No) | Yes | 1.06 | (0.85, 1.32) | 0.884 |
| Self-rated health growing up (Ref: Good) | Excellent | 1.14 | (0.96, 1.35) | 0.121 |
|  | Very good | 1.17 | (0.97, 1.40) |  |
|  | Fair | 0.97 | (0.83, 1.15) |  |
|  | Poor | 0.96 | (0.71, 1.30) |  |
| Immigration status (Ref: Born in this country) | Born in another country | 1.11 | (0.18, 6.90) | 1 |
| Age 12 religious service attendance (Ref: Never) | At least 1/week | 1.62 | (1.16, 2.28) | <.001 |
|  | 1-3/month | 1.32 | (0.94, 1.87) |  |
|  | <1/month | 1.12 | (0.77, 1.62) |  |
| Age group (Ref: 1998-2005; current age: 18-24) | 1993-1998; age: 25-29 | 1.02 | (0.79, 1.30) | 0.006 |
|  | 1983-1993; age: 30-39 | 1.12 | (0.93, 1.34) |  |
|  | 1973-1983; age 40-49 | 1.23 | (1.01, 1.52) |  |
|  | 1963-1973; age 50-59 | 1.22 | (0.99, 1.49) |  |
|  | 1953-1963; age 60-69 | 1.27 | (0.99, 1.64) |  |
|  | 1943-1953; age 70-79 | 1.70 | (1.22, 2.38) |  |
|  | 1943 or earlier; age 80+ | 1.49 | (0.72, 3.09) |  |
| Gender (Ref: Male) | Female | 0.82 | (0.73, 0.92) | <.001 |
|  | Other | 1.97 | (1.23, 3.18) |  |
| Religious affiliation at age 12 (Ref: Christianity) | Islam | 0.79 | (0.62, 1.00) | 0.035 |
|  | Some other religion | 1.13 | (0.62, 2.06) |  |
| Race and ethnicity plurality (Ref: Majority) | Minority | 1.23 | (1.07, 1.43) | <.001 |

***Table S14c. Sensitivity to unmeasured confounding of childhood predictors in Philippines***

| Variable | Category | E-value for Estimate | E-value for 95% CI |
| --- | --- | --- | --- |
| Relationship with mother (Ref: Very bad/Somewhat bad) | Very good/Somewhat good | 1.85 | 1.00 |
| Relationship with father (Ref: Very bad/Somewhat bad) | Very good/Somewhat good | 1.44 | 1.00 |
| Parent marital status (Ref: Parents married) | No, divorced | 1.79 | 1.00 |
|  | Never | 1.43 | 1.00 |
|  | No, one or both of them had died | 1.36 | 1.00 |
| Subjective financial status of family growing up (Ref: Got by) | Lived comfortably | 1.82 | 1.36 |
|  | Found it difficult | 1.14 | 1.00 |
|  | Found it very difficult | 1.24 | 1.00 |
| Abuse (Ref: No) | Yes | 1.30 | 1.00 |
| Outsider growing up (Ref: No) | Yes | 1.31 | 1.00 |
| Self-rated health growing up (Ref: Good) | Excellent | 1.54 | 1.00 |
|  | Very good | 1.61 | 1.00 |
|  | Fair | 1.20 | 1.00 |
|  | Poor | 1.25 | 1.00 |
| Immigration status (Ref: Born in this country) | Born in another country | 1.44 | 1.00 |
| Age 12 religious service attendance (Ref: Never) | At least 1/week | 2.63 | 1.58 |
|  | 1-3/month | 1.98 | 1.00 |
|  | <1/month | 1.47 | 1.00 |
| Age group (Ref: 1998-2005; current age: 18-24) | 1993-1998; age: 25-29 | 1.15 | 1.00 |
|  | 1983-1993; age: 30-39 | 1.48 | 1.00 |
|  | 1973-1983; age 40-49 | 1.78 | 1.09 |
|  | 1963-1973; age 50-59 | 1.73 | 1.00 |
|  | 1953-1963; age 60-69 | 1.86 | 1.00 |
|  | 1943-1953; age 70-79 | 2.80 | 1.73 |
|  | 1943 or earlier; age 80+ | 2.34 | 1.00 |
| Gender (Ref: Male) | Female | 1.73 | 1.38 |
|  | Other | 3.37 | 1.76 |
| Religious affiliation at age 12 (Ref: Christianity) | Some other religion | 1.52 | 1.00 |
| Race and ethnicity plurality (Ref: Majority) | Minority | 1.77 | 1.33 |
|  | Islam | 1.85 | 1.00 |

***Table S15a. Nationally representative descriptive statistics for Poland***

| **Characteristic** | **N = 10,389**^1^ |
| --- | --- |
| **Relationship with mother** |  |
| Very good | 4,879 (47%) |
| Somewhat good | 4,973 (48%) |
| Somewhat bad | 285 (2.7%) |
| Very bad | 58 (0.6%) |
| Does not apply | 80 (0.8%) |
| Missing | 112 (1.1%) |
| **Relationship with father** |  |
| Very good | 4,231 (41%) |
| Somewhat good | 4,984 (48%) |
| Somewhat bad | 516 (5.0%) |
| Very bad | 78 (0.7%) |
| Does not apply | 407 (3.9%) |
| Missing | 173 (1.7%) |
| **Parent marital status** |  |
| Yes, married | 8,972 (86%) |
| No, divorced | 587 (5.7%) |
| Never married | 193 (1.9%) |
| No, one or both of them had died | 313 (3.0%) |
| Missing | 324 (3.1%) |
| **Subjective financial status of family growing up** |  |
| Lived comfortably | 1,384 (13%) |
| Got by | 6,257 (60%) |
| Found it difficult | 2,133 (21%) |
| Found it very difficult | 509 (4.9%) |
| Missing | 106 (1.0%) |
| **Abuse** |  |
| Yes | 325 (3.1%) |
| No | 10,009 (96%) |
| Missing | 55 (0.5%) |
| **Outsider growing up** |  |
| Yes | 490 (4.7%) |
| No | 9,615 (93%) |
| Missing | 284 (2.7%) |
| **Self-rated health growing up** |  |
| Excellent | 2,676 (26%) |
| Very good | 5,371 (52%) |
| Good | 1,779 (17%) |
| Fair | 406 (3.9%) |
| Poor | 123 (1.2%) |
| Missing | 34 (0.3%) |
| **Immigration status** |  |
| Born in this country | 10,258 (99%) |
| Born in another country | 108 (1.0%) |
| Missing | 23 (0.2%) |
| **Age 12 religious service attendance** |  |
| At least 1/week | 4,751 (46%) |
| 1-3/month | 2,689 (26%) |
| <1/month | 2,161 (21%) |
| Never | 354 (3.4%) |
| Missing | 434 (4.2%) |
| **Age group** |  |
| 1998-2005; current age: 18-24 | 955 (9.2%) |
| 1993-1998; age: 25-29 | 761 (7.3%) |
| 1983-1993; age: 30-39 | 2,159 (21%) |
| 1973-1983; age 40-49 | 1,956 (19%) |
| 1963-1973; age 50-59 | 1,670 (16%) |
| 1953-1963; age 60-69 | 1,909 (18%) |
| 1943-1953; age 70-79 | 833 (8.0%) |
| 1943 or earlier; age 80+ | 145 (1.4%) |
| Missing | 1 (<0.1%) |
| **Gender** |  |
| Male | 4,974 (48%) |
| Female | 5,387 (52%) |
| Other | 3 (<0.1%) |
| Missing | 26 (0.2%) |
| **Religious affiliation at age 12** |  |
| Buddhism | 2 (<0.1%) |
| Christianity | 9,861 (95%) |
| Islam | 3 (<0.1%) |
| No religion/Atheist/Agnostic | 482 (4.6%) |
| Primal, Animist, or Folk religion | 5 (<0.1%) |
| Sikhism | 1 (<0.1%) |
| Missing | 35 (0.3%) |
| **Race and ethnicity** |  |
| Belarussian | 2 (<0.1%) |
| German | 4 (<0.1%) |
| Kashubians | 3 (<0.1%) |
| Other | 4 (<0.1%) |
| Polish | 10,309 (99%) |
| Silesia | 14 (0.1%) |
| Ukrainian | 38 (0.4%) |
| Missing | 14 (0.1%) |
| ^1^n (%) | |

***Table S15b. Regression of volunteering on childhood predictors for Poland***

| Variable | Category | Risk ratio | 95% CI | Global p-value |
| --- | --- | --- | --- | --- |
| Relationship with mother (Ref: Very bad/Somewhat bad) | Very good/Somewhat good | 0.59 | (0.33, 1.07) | <.001 |
| Relationship with father (Ref: Very bad/Somewhat bad) | Very good/Somewhat good | 1.39 | (0.86, 2.26) | <.001 |
| Parent marital status (Ref: Parents married) | No, divorced | 1.20 | (0.79, 1.81) | 0.036 |
|  | Never | 1.42 | (0.91, 2.23) |  |
|  | No, one or both of them had died | 1.58 | (0.90, 2.78) |  |
| Subjective financial status of family growing up (Ref: Got by) | Lived comfortably | 1.49 | (1.17, 1.89) | <.001 |
|  | Found it difficult | 1.07 | (0.85, 1.35) |  |
|  | Found it very difficult | 0.90 | (0.55, 1.47) |  |
| Abuse (Ref: No) | Yes | 0.90 | (0.55, 1.48) | 0.964 |
| Outsider growing up (Ref: No) | Yes | 1.49 | (0.97, 2.31) | 0.002 |
| Self-rated health growing up (Ref: Good) | Excellent | 0.73 | (0.54, 1.00) | <.001 |
|  | Very good | 0.69 | (0.52, 0.92) |  |
|  | Fair | 1.84 | (1.29, 2.66) |  |
|  | Poor | 1.31 | (0.50, 3.46) |  |
| Immigration status (Ref: Born in this country) | Born in another country | 2.29 | (1.26, 4.16) | <.001 |
| Age 12 religious service attendance (Ref: Never) | At least 1/week | 1.39 | (0.82, 2.35) | <.001 |
|  | 1-3/month | 1.68 | (0.98, 2.90) |  |
|  | <1/month | 1.01 | (0.60, 1.72) |  |
| Age group (Ref: 1998-2005; current age: 18-24) | 1993-1998; age: 25-29 | 0.85 | (0.60, 1.21) | <.001 |
|  | 1983-1993; age: 30-39 | 0.88 | (0.64, 1.21) |  |
|  | 1973-1983; age 40-49 | 1.01 | (0.72, 1.42) |  |
|  | 1963-1973; age 50-59 | 0.79 | (0.56, 1.11) |  |
|  | 1953-1963; age 60-69 | 1.32 | (0.92, 1.90) |  |
|  | 1943-1953; age 70-79 | 0.95 | (0.58, 1.55) |  |
|  | 1943 or earlier; age 80+ | 0.95 | (0.28, 3.17) |  |
| Gender (Ref: Male) | Female | 1.34 | (1.12, 1.61) | <.001 |
|  | Other | 0.00 | (0.00, 0.00) |  |
| Religious affiliation at age 12 (Ref: No religion/Atheist/Agnostic) | Christianity | 0.85 | (0.52, 1.38) | 0.976 |
|  | Some other religion | 0.77 | (0.22, 2.71) |  |
| Race and ethnicity plurality (Ref: Majority) | Minority | 0.81 | (0.40, 1.63) | 0.824 |

***Table S15c. Sensitivity to unmeasured confounding of childhood predictors in Poland***

| Variable | Category | E-value for Estimate | E-value for 95% CI |
| --- | --- | --- | --- |
| Relationship with mother (Ref: Very bad/Somewhat bad) | Very good/Somewhat good | 2.77 | 1.00 |
| Relationship with father (Ref: Very bad/Somewhat bad) | Very good/Somewhat good | 2.13 | 1.00 |
| Parent marital status (Ref: Parents married) | No, divorced | 1.68 | 1.00 |
|  | Never | 2.20 | 1.00 |
|  | No, one or both of them had died | 2.55 | 1.00 |
| Subjective financial status of family growing up (Ref: Got by) | Lived comfortably | 2.34 | 1.62 |
|  | Found it difficult | 1.35 | 1.00 |
|  | Found it very difficult | 1.46 | 1.00 |
| Abuse (Ref: No) | Yes | 1.44 | 1.00 |
| Outsider growing up (Ref: No) | Yes | 2.36 | 1.00 |
| Self-rated health growing up (Ref: Good) | Excellent | 2.07 | 1.01 |
|  | Very good | 2.25 | 1.39 |
|  | Fair | 3.10 | 1.89 |
|  | Poor | 1.95 | 1.00 |
| Immigration status (Ref: Born in this country) | Born in another country | 4.01 | 1.83 |
| Age 12 religious service attendance (Ref: Never) | At least 1/week | 2.13 | 1.00 |
|  | 1-3/month | 2.75 | 1.00 |
|  | <1/month | 1.14 | 1.00 |
| Age group (Ref: 1998-2005; current age: 18-24) | 1993-1998; age: 25-29 | 1.63 | 1.00 |
|  | 1983-1993; age: 30-39 | 1.54 | 1.00 |
|  | 1973-1983; age 40-49 | 1.12 | 1.00 |
|  | 1963-1973; age 50-59 | 1.86 | 1.00 |
|  | 1953-1963; age 60-69 | 1.97 | 1.00 |
|  | 1943-1953; age 70-79 | 1.28 | 1.00 |
|  | 1943 or earlier; age 80+ | 1.30 | 1.00 |
| Gender (Ref: Male) | Female | 2.01 | 1.47 |
|  | Other | 85,523.43 | 24,591.88 |
| Religious affiliation at age 12 (Ref: No religion/Atheist/Agnostic) | Christianity | 1.63 | 1.00 |
|  | Some other religion | 1.93 | 1.00 |
| Race and ethnicity plurality (Ref: Majority) | Minority | 1.77 | 1.00 |

***Table S16a. Nationally representative descriptive statistics for South Africa***

| **Characteristic** | **N = 2,651**^1^ |
| --- | --- |
| **Relationship with mother** |  |
| Very good | 2,186 (82%) |
| Somewhat good | 263 (9.9%) |
| Somewhat bad | 51 (1.9%) |
| Very bad | 39 (1.5%) |
| Does not apply | 90 (3.4%) |
| Missing | 21 (0.8%) |
| **Relationship with father** |  |
| Very good | 1,656 (62%) |
| Somewhat good | 333 (13%) |
| Somewhat bad | 86 (3.3%) |
| Very bad | 159 (6.0%) |
| Does not apply | 331 (12%) |
| Missing | 85 (3.2%) |
| **Parent marital status** |  |
| Yes, married | 1,321 (50%) |
| No, divorced | 131 (5.0%) |
| Never married | 904 (34%) |
| No, one or both of them had died | 140 (5.3%) |
| Missing | 155 (5.8%) |
| **Subjective financial status of family growing up** |  |
| Lived comfortably | 1,050 (40%) |
| Got by | 875 (33%) |
| Found it difficult | 432 (16%) |
| Found it very difficult | 289 (11%) |
| Missing | 5 (0.2%) |
| **Abuse** |  |
| Yes | 450 (17%) |
| No | 2,149 (81%) |
| Missing | 52 (2.0%) |
| **Outsider growing up** |  |
| Yes | 434 (16%) |
| No | 2,211 (83%) |
| Missing | 6 (0.2%) |
| **Self-rated health growing up** |  |
| Excellent | 1,225 (46%) |
| Very good | 590 (22%) |
| Good | 370 (14%) |
| Fair | 266 (10%) |
| Poor | 183 (6.9%) |
| Missing | 17 (0.6%) |
| **Immigration status** |  |
| Born in this country | 2,511 (95%) |
| Born in another country | 139 (5.2%) |
| Missing | 1 (<0.1%) |
| **Age 12 religious service attendance** |  |
| At least 1/week | 1,681 (63%) |
| 1-3/month | 552 (21%) |
| <1/month | 175 (6.6%) |
| Never | 217 (8.2%) |
| Missing | 26 (1.0%) |
| **Age group** |  |
| 1998-2005; current age: 18-24 | 461 (17%) |
| 1993-1998; age: 25-29 | 364 (14%) |
| 1983-1993; age: 30-39 | 655 (25%) |
| 1973-1983; age 40-49 | 522 (20%) |
| 1963-1973; age 50-59 | 309 (12%) |
| 1953-1963; age 60-69 | 195 (7.4%) |
| 1943-1953; age 70-79 | 120 (4.5%) |
| 1943 or earlier; age 80+ | 17 (0.6%) |
| Missing | 9 (0.3%) |
| **Gender** |  |
| Male | 1,288 (49%) |
| Female | 1,356 (51%) |
| Other | 2 (<0.1%) |
| Missing | 4 (0.2%) |
| **Religious affiliation at age 12** |  |
| Buddhism | 11 (0.4%) |
| Christianity | 2,323 (88%) |
| Hinduism | 2 (<0.1%) |
| Islam | 52 (2.0%) |
| No religion/Atheist/Agnostic | 107 (4.1%) |
| Primal, Animist, or Folk religion | 117 (4.4%) |
| Shinto | 2 (<0.1%) |
| Some other religion | 7 (0.3%) |
| Taoism | 1 (<0.1%) |
| Missing | 27 (1.0%) |
| **Race and ethnicity** |  |
| Asian/Indian | 6 (0.2%) |
| Black | 2,381 (90%) |
| Colored | 252 (9.5%) |
| Other | 1 (<0.1%) |
| White | 8 (0.3%) |
| Missing | 3 (0.1%) |
| ^1^n (%) | |

***Table S16b. Regression of volunteering on childhood predictors for South Africa***

| Variable | Category | Risk ratio | 95% CI | Global p-value |
| --- | --- | --- | --- | --- |
| Relationship with mother (Ref: Very bad/Somewhat bad) | Very good/Somewhat good | 0.86 | (0.59, 1.26) | 0.082 |
| Relationship with father (Ref: Very bad/Somewhat bad) | Very good/Somewhat good | 0.91 | (0.70, 1.19) | 0.171 |
| Parent marital status (Ref: Parents married) | No, divorced | 0.84 | (0.55, 1.28) | 0.985 |
|  | Never | 0.94 | (0.77, 1.15) |  |
|  | No, one or both of them had died | 0.90 | (0.60, 1.36) |  |
| Subjective financial status of family growing up (Ref: Got by) | Lived comfortably | 0.98 | (0.81, 1.18) | 0.998 |
|  | Found it difficult | 0.98 | (0.75, 1.29) |  |
|  | Found it very difficult | 0.87 | (0.64, 1.18) |  |
| Abuse (Ref: No) | Yes | 1.35 | (1.11, 1.66) | <.001 |
| Outsider growing up (Ref: No) | Yes | 1.16 | (0.93, 1.46) | 0.078 |
| Self-rated health growing up (Ref: Good) | Excellent | 1.03 | (0.82, 1.30) | 1 |
|  | Very good | 1.02 | (0.75, 1.40) |  |
|  | Fair | 0.98 | (0.71, 1.36) |  |
|  | Poor | 0.96 | (0.65, 1.43) |  |
| Immigration status (Ref: Born in this country) | Born in another country | 0.45 | (0.23, 0.88) | <.001 |
| Age 12 religious service attendance (Ref: Never) | At least 1/week | 1.68 | (0.94, 3.05) | 0.003 |
|  | 1-3/month | 1.45 | (0.82, 2.59) |  |
|  | <1/month | 2.10 | (1.11, 4.00) |  |
| Age group (Ref: 1998-2005; current age: 18-24) | 1993-1998; age: 25-29 | 1.03 | (0.81, 1.32) | 0.907 |
|  | 1983-1993; age: 30-39 | 1.02 | (0.79, 1.32) |  |
|  | 1973-1983; age 40-49 | 1.04 | (0.78, 1.39) |  |
|  | 1963-1973; age 50-59 | 1.11 | (0.81, 1.51) |  |
|  | 1953-1963; age 60-69 | 1.01 | (0.63, 1.61) |  |
|  | 1943-1953; age 70-79 | 1.07 | (0.59, 1.96) |  |
|  | 1943 or earlier; age 80+ | 0.11 | (0.01, 0.91) |  |
| Gender (Ref: Male) | Female | 0.84 | (0.70, 1.01) | 0.048 |
|  | Other | 1.43 | (0.32, 6.54) |  |
| Religious affiliation at age 12 (Ref: No religion/Atheist/Agnostic) | Christianity | 1.65 | (0.68, 3.95) | 0.259 |
|  | Primal, Animist, or Folk religion | 0.99 | (0.39, 2.50) |  |
|  | Some other religion | 1.32 | (0.47, 3.72) |  |
| Race and ethnicity plurality (Ref: Majority) | Minority | 0.91 | (0.61, 1.37) | 0.944 |

***Table S16c. Sensitivity to unmeasured confounding of childhood predictors in South Africa***

| Variable | Category | E-value for Estimate | E-value for 95% CI |
| --- | --- | --- | --- |
| Relationship with mother (Ref: Very bad/Somewhat bad) | Very good/Somewhat good | 1.60 | 1.00 |
| Relationship with father (Ref: Very bad/Somewhat bad) | Very good/Somewhat good | 1.42 | 1.00 |
| Parent marital status (Ref: Parents married) | No, divorced | 1.68 | 1.00 |
|  | Never | 1.33 | 1.00 |
|  | No, one or both of them had died | 1.45 | 1.00 |
| Subjective financial status of family growing up (Ref: Got by) | Lived comfortably | 1.18 | 1.00 |
|  | Found it difficult | 1.16 | 1.00 |
|  | Found it very difficult | 1.56 | 1.00 |
| Abuse (Ref: No) | Yes | 2.05 | 1.45 |
| Outsider growing up (Ref: No) | Yes | 1.60 | 1.00 |
| Self-rated health growing up (Ref: Good) | Excellent | 1.20 | 1.00 |
|  | Very good | 1.18 | 1.00 |
|  | Fair | 1.15 | 1.00 |
|  | Poor | 1.24 | 1.00 |
| Immigration status (Ref: Born in this country) | Born in another country | 3.83 | 1.52 |
| Age 12 religious service attendance (Ref: Never) | At least 1/week | 2.77 | 1.00 |
|  | 1-3/month | 2.27 | 1.00 |
|  | <1/month | 3.63 | 1.45 |
| Age group (Ref: 1998-2005; current age: 18-24) | 1993-1998; age: 25-29 | 1.21 | 1.00 |
|  | 1983-1993; age: 30-39 | 1.16 | 1.00 |
|  | 1973-1983; age 40-49 | 1.26 | 1.00 |
|  | 1963-1973; age 50-59 | 1.45 | 1.00 |
|  | 1953-1963; age 60-69 | 1.12 | 1.00 |
|  | 1943-1953; age 70-79 | 1.36 | 1.00 |
|  | 1943 or earlier; age 80+ | 17.82 | 1.44 |
| Gender (Ref: Male) | Female | 1.66 | 1.00 |
|  | Other | 2.24 | 1.00 |
| Religious affiliation at age 12 (Ref: No religion/Atheist/Agnostic) | Christianity | 2.67 | 1.00 |
|  | Some other religion | 1.98 | 1.00 |
| Race and ethnicity plurality (Ref: Majority) | Minority | 1.41 | 1.00 |
|  | Primal, Animist, or Folk religion | 1.12 | 1.00 |

***Table S17a. Nationally representative descriptive statistics for Spain***

| **Characteristic** | **N = 6,290**^1^ |
| --- | --- |
| **Relationship with mother** |  |
| Very good | 4,557 (72%) |
| Somewhat good | 1,258 (20%) |
| Somewhat bad | 248 (3.9%) |
| Very bad | 92 (1.5%) |
| Does not apply | 107 (1.7%) |
| Missing | 28 (0.4%) |
| **Relationship with father** |  |
| Very good | 4,131 (66%) |
| Somewhat good | 1,397 (22%) |
| Somewhat bad | 309 (4.9%) |
| Very bad | 178 (2.8%) |
| Does not apply | 243 (3.9%) |
| Missing | 33 (0.5%) |
| **Parent marital status** |  |
| Yes, married | 5,285 (84%) |
| No, divorced | 378 (6.0%) |
| Never married | 312 (5.0%) |
| No, one or both of them had died | 126 (2.0%) |
| Missing | 188 (3.0%) |
| **Subjective financial status of family growing up** |  |
| Lived comfortably | 2,041 (32%) |
| Got by | 2,956 (47%) |
| Found it difficult | 1,154 (18%) |
| Found it very difficult | 110 (1.7%) |
| Missing | 29 (0.5%) |
| **Abuse** |  |
| Yes | 659 (10%) |
| No | 5,510 (88%) |
| Missing | 122 (1.9%) |
| **Outsider growing up** |  |
| Yes | 579 (9.2%) |
| No | 5,637 (90%) |
| Missing | 75 (1.2%) |
| **Self-rated health growing up** |  |
| Excellent | 2,450 (39%) |
| Very good | 2,286 (36%) |
| Good | 1,235 (20%) |
| Fair | 164 (2.6%) |
| Poor | 135 (2.1%) |
| Missing | 20 (0.3%) |
| **Immigration status** |  |
| Born in this country | 5,479 (87%) |
| Born in another country | 788 (13%) |
| Missing | 23 (0.4%) |
| **Age 12 religious service attendance** |  |
| At least 1/week | 2,391 (38%) |
| 1-3/month | 1,132 (18%) |
| <1/month | 1,287 (20%) |
| Never | 1,445 (23%) |
| Missing | 36 (0.6%) |
| **Age group** |  |
| 1998-2005; current age: 18-24 | 594 (9.4%) |
| 1993-1998; age: 25-29 | 450 (7.2%) |
| 1983-1993; age: 30-39 | 1,111 (18%) |
| 1973-1983; age 40-49 | 1,396 (22%) |
| 1963-1973; age 50-59 | 1,252 (20%) |
| 1953-1963; age 60-69 | 977 (16%) |
| 1943-1953; age 70-79 | 467 (7.4%) |
| 1943 or earlier; age 80+ | 43 (0.7%) |
| Missing | 0 (0%) |
| **Gender** |  |
| Male | 3,142 (50%) |
| Female | 3,119 (50%) |
| Other | 6 (0.1%) |
| Missing | 22 (0.4%) |
| **Religious affiliation at age 12** |  |
| Buddhism | 8 (0.1%) |
| Christianity | 5,119 (81%) |
| Confucianism | 1 (<0.1%) |
| Hinduism | 5 (<0.1%) |
| Islam | 132 (2.1%) |
| Judaism | 5 (<0.1%) |
| No religion/Atheist/Agnostic | 972 (15%) |
| Primal, Animist, or Folk religion | 4 (<0.1%) |
| Sikhism | 2 (<0.1%) |
| Some other religion | 13 (0.2%) |
| Missing | 29 (0.5%) |
| **Race and ethnicity** |  |
| Missing | 6,290 (100%) |
| ^1^n (%) | |

***Table S17b. Regression of volunteering on childhood predictors for Spain***

| Variable | Category | Risk ratio | 95% CI | Global p-value |
| --- | --- | --- | --- | --- |
| Relationship with mother (Ref: Very bad/Somewhat bad) | Very good/Somewhat good | 1.17 | (0.88, 1.57) | 0.003 |
| Relationship with father (Ref: Very bad/Somewhat bad) | Very good/Somewhat good | 0.94 | (0.74, 1.20) | 0.48 |
| Parent marital status (Ref: Parents married) | No, divorced | 0.82 | (0.63, 1.07) | 0.357 |
|  | Never | 1.09 | (0.83, 1.44) |  |
|  | No, one or both of them had died | 0.84 | (0.52, 1.39) |  |
| Subjective financial status of family growing up (Ref: Got by) | Lived comfortably | 0.94 | (0.79, 1.12) | 0.009 |
|  | Found it difficult | 0.87 | (0.71, 1.08) |  |
|  | Found it very difficult | 1.43 | (0.95, 2.14) |  |
| Abuse (Ref: No) | Yes | 1.00 | (0.81, 1.24) | 1 |
| Outsider growing up (Ref: No) | Yes | 1.35 | (1.13, 1.62) | <.001 |
| Self-rated health growing up (Ref: Good) | Excellent | 0.91 | (0.75, 1.12) | 1 |
|  | Very good | 0.94 | (0.77, 1.16) |  |
|  | Fair | 1.02 | (0.61, 1.69) |  |
|  | Poor | 0.86 | (0.52, 1.42) |  |
| Immigration status (Ref: Born in this country) | Born in another country | 1.03 | (0.85, 1.24) | 0.993 |
| Age 12 religious service attendance (Ref: Never) | At least 1/week | 1.20 | (0.98, 1.47) | <.001 |
|  | 1-3/month | 1.05 | (0.84, 1.31) |  |
|  | <1/month | 0.88 | (0.69, 1.11) |  |
| Age group (Ref: 1998-2005; current age: 18-24) | 1993-1998; age: 25-29 | 1.20 | (0.91, 1.58) | <.001 |
|  | 1983-1993; age: 30-39 | 1.08 | (0.85, 1.37) |  |
|  | 1973-1983; age 40-49 | 0.88 | (0.69, 1.11) |  |
|  | 1963-1973; age 50-59 | 0.89 | (0.69, 1.15) |  |
|  | 1953-1963; age 60-69 | 0.68 | (0.50, 0.95) |  |
|  | 1943-1953; age 70-79 | 0.81 | (0.53, 1.24) |  |
|  | 1943 or earlier; age 80+ | 0.40 | (0.11, 1.53) |  |
| Gender (Ref: Male) | Female | 0.58 | (0.50, 0.67) | <.001 |
|  | Other | 0.99 | (0.35, 2.84) |  |
| Religious affiliation at age 12 (Ref: No religion/Atheist/Agnostic) | Christianity | 2.01 | (1.54, 2.65) | <.001 |
|  | Some other religion | 2.72 | (1.82, 4.08) |  |

***Table S17c. Sensitivity to unmeasured confounding of childhood predictors in Spain***

| Variable | Category | E-value for Estimate | E-value for 95% CI |
| --- | --- | --- | --- |
| Relationship with mother (Ref: Very bad/Somewhat bad) | Very good/Somewhat good | 1.63 | 1.00 |
| Relationship with father (Ref: Very bad/Somewhat bad) | Very good/Somewhat good | 1.32 | 1.00 |
| Parent marital status (Ref: Parents married) | No, divorced | 1.73 | 1.00 |
|  | Never | 1.41 | 1.00 |
|  | No, one or both of them had died | 1.64 | 1.00 |
| Subjective financial status of family growing up (Ref: Got by) | Lived comfortably | 1.33 | 1.00 |
|  | Found it difficult | 1.55 | 1.00 |
|  | Found it very difficult | 2.21 | 1.00 |
| Abuse (Ref: No) | Yes | 1.06 | 1.00 |
| Outsider growing up (Ref: No) | Yes | 2.05 | 1.51 |
| Self-rated health growing up (Ref: Good) | Excellent | 1.41 | 1.00 |
|  | Very good | 1.32 | 1.00 |
|  | Fair | 1.15 | 1.00 |
|  | Poor | 1.60 | 1.00 |
| Immigration status (Ref: Born in this country) | Born in another country | 1.19 | 1.00 |
| Age 12 religious service attendance (Ref: Never) | At least 1/week | 1.69 | 1.00 |
|  | 1-3/month | 1.28 | 1.00 |
|  | <1/month | 1.54 | 1.00 |
| Age group (Ref: 1998-2005; current age: 18-24) | 1993-1998; age: 25-29 | 1.68 | 1.00 |
|  | 1983-1993; age: 30-39 | 1.38 | 1.00 |
|  | 1973-1983; age 40-49 | 1.55 | 1.00 |
|  | 1963-1973; age 50-59 | 1.49 | 1.00 |
|  | 1953-1963; age 60-69 | 2.27 | 1.30 |
|  | 1943-1953; age 70-79 | 1.76 | 1.00 |
|  | 1943 or earlier; age 80+ | 4.38 | 1.00 |
| Gender (Ref: Male) | Female | 2.84 | 2.33 |
|  | Other | 1.11 | 1.00 |
| Religious affiliation at age 12 (Ref: No religion/Atheist/Agnostic) | Christianity | 3.46 | 2.45 |
|  | Some other religion | 4.89 | 3.03 |

***Table S18a. Nationally representative descriptive statistics for Sweden***

| **Characteristic** | **N = 15,068**^1^ |
| --- | --- |
| **Relationship with mother** |  |
| Very good | 8,743 (58%) |
| Somewhat good | 4,513 (30%) |
| Somewhat bad | 1,194 (7.9%) |
| Very bad | 371 (2.5%) |
| Does not apply | 216 (1.4%) |
| Missing | 30 (0.2%) |
| **Relationship with father** |  |
| Very good | 7,134 (47%) |
| Somewhat good | 4,885 (32%) |
| Somewhat bad | 1,588 (11%) |
| Very bad | 725 (4.8%) |
| Does not apply | 720 (4.8%) |
| Missing | 16 (0.1%) |
| **Parent marital status** |  |
| Yes, married | 10,887 (72%) |
| No, divorced | 1,927 (13%) |
| Never married | 1,747 (12%) |
| No, one or both of them had died | 362 (2.4%) |
| Missing | 145 (1.0%) |
| **Subjective financial status of family growing up** |  |
| Lived comfortably | 5,951 (39%) |
| Got by | 7,717 (51%) |
| Found it difficult | 1,238 (8.2%) |
| Found it very difficult | 140 (0.9%) |
| Missing | 22 (0.1%) |
| **Abuse** |  |
| Yes | 2,288 (15%) |
| No | 12,735 (85%) |
| Missing | 45 (0.3%) |
| **Outsider growing up** |  |
| Yes | 1,867 (12%) |
| No | 13,034 (86%) |
| Missing | 168 (1.1%) |
| **Self-rated health growing up** |  |
| Excellent | 5,733 (38%) |
| Very good | 5,124 (34%) |
| Good | 2,669 (18%) |
| Fair | 1,108 (7.4%) |
| Poor | 397 (2.6%) |
| Missing | 38 (0.2%) |
| **Immigration status** |  |
| Born in this country | 13,922 (92%) |
| Born in another country | 1,052 (7.0%) |
| Missing | 94 (0.6%) |
| **Age 12 religious service attendance** |  |
| At least 1/week | 955 (6.3%) |
| 1-3/month | 1,362 (9.0%) |
| <1/month | 6,224 (41%) |
| Never | 6,472 (43%) |
| Missing | 54 (0.4%) |
| **Age group** |  |
| 1998-2005; current age: 18-24 | 1,515 (10%) |
| 1993-1998; age: 25-29 | 1,399 (9.3%) |
| 1983-1993; age: 30-39 | 2,398 (16%) |
| 1973-1983; age 40-49 | 2,221 (15%) |
| 1963-1973; age 50-59 | 2,493 (17%) |
| 1953-1963; age 60-69 | 2,168 (14%) |
| 1943-1953; age 70-79 | 2,253 (15%) |
| 1943 or earlier; age 80+ | 621 (4.1%) |
| Missing | 0 (0%) |
| **Gender** |  |
| Male | 7,536 (50%) |
| Female | 7,493 (50%) |
| Other | 27 (0.2%) |
| Missing | 12 (<0.1%) |
| **Religious affiliation at age 12** |  |
| Baha’i | 3 (<0.1%) |
| Buddhism | 41 (0.3%) |
| Christianity | 10,617 (70%) |
| Confucianism | 4 (<0.1%) |
| Hinduism | 16 (0.1%) |
| Islam | 462 (3.1%) |
| Judaism | 51 (0.3%) |
| No religion/Atheist/Agnostic | 3,738 (25%) |
| Primal, Animist, or Folk religion | 31 (0.2%) |
| Shinto | 1 (<0.1%) |
| Sikhism | 9 (<0.1%) |
| Some other religion | 69 (0.5%) |
| Missing | 26 (0.2%) |
| **Race and ethnicity** |  |
| Missing | 15,068 (100%) |
| ^1^n (%) | |

***Table S18b. Regression of volunteering on childhood predictors for Sweden***

| Variable | Category | Risk ratio | 95% CI | Global p-value |
| --- | --- | --- | --- | --- |
| Relationship with mother (Ref: Very bad/Somewhat bad) | Very good/Somewhat good | 0.90 | (0.72, 1.11) | 0.008 |
| Relationship with father (Ref: Very bad/Somewhat bad) | Very good/Somewhat good | 1.06 | (0.88, 1.29) | 0.174 |
| Parent marital status (Ref: Parents married) | No, divorced | 0.86 | (0.70, 1.05) | 0.003 |
|  | Never | 0.75 | (0.59, 0.95) |  |
|  | No, one or both of them had died | 0.76 | (0.45, 1.26) |  |
| Subjective financial status of family growing up (Ref: Got by) | Lived comfortably | 1.09 | (0.96, 1.25) | 0.024 |
|  | Found it difficult | 1.14 | (0.91, 1.42) |  |
|  | Found it very difficult | 0.56 | (0.28, 1.12) |  |
| Abuse (Ref: No) | Yes | 1.48 | (1.26, 1.74) | <.001 |
| Outsider growing up (Ref: No) | Yes | 1.25 | (1.02, 1.52) | <.001 |
| Self-rated health growing up (Ref: Good) | Excellent | 1.23 | (1.02, 1.49) | 0.008 |
|  | Very good | 1.12 | (0.93, 1.35) |  |
|  | Fair | 0.90 | (0.68, 1.20) |  |
|  | Poor | 1.15 | (0.80, 1.67) |  |
| Immigration status (Ref: Born in this country) | Born in another country | 1.04 | (0.83, 1.30) | 0.971 |
| Age 12 religious service attendance (Ref: Never) | At least 1/week | 3.53 | (2.90, 4.32) | <.001 |
|  | 1-3/month | 2.51 | (2.05, 3.07) |  |
|  | <1/month | 1.68 | (1.44, 1.96) |  |
| Age group (Ref: 1998-2005; current age: 18-24) | 1993-1998; age: 25-29 | 0.90 | (0.69, 1.17) | <.001 |
|  | 1983-1993; age: 30-39 | 0.80 | (0.63, 1.02) |  |
|  | 1973-1983; age 40-49 | 0.96 | (0.76, 1.22) |  |
|  | 1963-1973; age 50-59 | 0.97 | (0.77, 1.23) |  |
|  | 1953-1963; age 60-69 | 0.70 | (0.55, 0.90) |  |
|  | 1943-1953; age 70-79 | 0.96 | (0.76, 1.23) |  |
|  | 1943 or earlier; age 80+ | 0.98 | (0.71, 1.36) |  |
| Gender (Ref: Male) | Female | 1.01 | (0.89, 1.15) | 0.003 |
|  | Other | 2.36 | (1.13, 4.91) |  |
| Religious affiliation at age 12 (Ref: No religion/Atheist/Agnostic) | Christianity | 1.06 | (0.89, 1.26) | 0.947 |
|  | Some other religion | 0.95 | (0.68, 1.34) |  |

***Table S18c. Sensitivity to unmeasured confounding of childhood predictors in Sweden***

| Variable | Category | E-value for Estimate | E-value for 95% CI |
| --- | --- | --- | --- |
| Relationship with mother (Ref: Very bad/Somewhat bad) | Very good/Somewhat good | 1.49 | 1.00 |
| Relationship with father (Ref: Very bad/Somewhat bad) | Very good/Somewhat good | 1.33 | 1.00 |
| Parent marital status (Ref: Parents married) | No, divorced | 1.61 | 1.00 |
|  | Never | 2.00 | 1.27 |
|  | No, one or both of them had died | 1.98 | 1.00 |
| Subjective financial status of family growing up (Ref: Got by) | Lived comfortably | 1.41 | 1.00 |
|  | Found it difficult | 1.53 | 1.00 |
|  | Found it very difficult | 2.96 | 1.00 |
| Abuse (Ref: No) | Yes | 2.32 | 1.82 |
| Outsider growing up (Ref: No) | Yes | 1.80 | 1.16 |
| Self-rated health growing up (Ref: Good) | Excellent | 1.77 | 1.17 |
|  | Very good | 1.49 | 1.00 |
|  | Fair | 1.45 | 1.00 |
|  | Poor | 1.58 | 1.00 |
| Immigration status (Ref: Born in this country) | Born in another country | 1.25 | 1.00 |
| Age 12 religious service attendance (Ref: Never) | At least 1/week | 6.54 | 5.26 |
|  | 1-3/month | 4.45 | 3.51 |
|  | <1/month | 2.75 | 2.24 |
| Age group (Ref: 1998-2005; current age: 18-24) | 1993-1998; age: 25-29 | 1.46 | 1.00 |
|  | 1983-1993; age: 30-39 | 1.80 | 1.00 |
|  | 1973-1983; age 40-49 | 1.24 | 1.00 |
|  | 1963-1973; age 50-59 | 1.19 | 1.00 |
|  | 1953-1963; age 60-69 | 2.20 | 1.45 |
|  | 1943-1953; age 70-79 | 1.23 | 1.00 |
|  | 1943 or earlier; age 80+ | 1.15 | 1.00 |
| Gender (Ref: Male) | Female | 1.12 | 1.00 |
|  | Other | 4.15 | 1.52 |
| Religious affiliation at age 12 (Ref: No religion/Atheist/Agnostic) | Christianity | 1.31 | 1.00 |
|  | Some other religion | 1.27 | 1.00 |

***Table S19a. Nationally representative descriptive statistics for Tanzania***

| **Characteristic** | **N = 9,075**^1^ |
| --- | --- |
| **Relationship with mother** |  |
| Very good | 7,739 (85%) |
| Somewhat good | 796 (8.8%) |
| Somewhat bad | 84 (0.9%) |
| Very bad | 84 (0.9%) |
| Does not apply | 303 (3.3%) |
| Missing | 70 (0.8%) |
| **Relationship with father** |  |
| Very good | 6,831 (75%) |
| Somewhat good | 1,101 (12%) |
| Somewhat bad | 203 (2.2%) |
| Very bad | 247 (2.7%) |
| Does not apply | 550 (6.1%) |
| Missing | 142 (1.6%) |
| **Parent marital status** |  |
| Yes, married | 6,929 (76%) |
| No, divorced | 678 (7.5%) |
| Never married | 751 (8.3%) |
| No, one or both of them had died | 313 (3.4%) |
| Missing | 404 (4.4%) |
| **Subjective financial status of family growing up** |  |
| Lived comfortably | 2,611 (29%) |
| Got by | 2,909 (32%) |
| Found it difficult | 2,679 (30%) |
| Found it very difficult | 814 (9.0%) |
| Missing | 61 (0.7%) |
| **Abuse** |  |
| Yes | 716 (7.9%) |
| No | 8,328 (92%) |
| Missing | 32 (0.3%) |
| **Outsider growing up** |  |
| Yes | 734 (8.1%) |
| No | 8,320 (92%) |
| Missing | 22 (0.2%) |
| **Self-rated health growing up** |  |
| Excellent | 2,406 (27%) |
| Very good | 2,036 (22%) |
| Good | 2,946 (32%) |
| Fair | 1,177 (13%) |
| Poor | 456 (5.0%) |
| Missing | 54 (0.6%) |
| **Immigration status** |  |
| Born in this country | 9,048 (100%) |
| Born in another country | 25 (0.3%) |
| Missing | 1 (<0.1%) |
| **Age 12 religious service attendance** |  |
| At least 1/week | 5,580 (61%) |
| 1-3/month | 2,383 (26%) |
| <1/month | 333 (3.7%) |
| Never | 595 (6.6%) |
| Missing | 184 (2.0%) |
| **Age group** |  |
| 1998-2005; current age: 18-24 | 2,284 (25%) |
| 1993-1998; age: 25-29 | 1,349 (15%) |
| 1983-1993; age: 30-39 | 2,060 (23%) |
| 1973-1983; age 40-49 | 1,503 (17%) |
| 1963-1973; age 50-59 | 912 (10%) |
| 1953-1963; age 60-69 | 575 (6.3%) |
| 1943-1953; age 70-79 | 297 (3.3%) |
| 1943 or earlier; age 80+ | 93 (1.0%) |
| Missing | 2 (<0.1%) |
| **Gender** |  |
| Male | 4,299 (47%) |
| Female | 4,776 (53%) |
| Other | 0 (0%) |
| Missing | 0 (0%) |
| **Religious affiliation at age 12** |  |
| Baha’i | 1 (<0.1%) |
| Christianity | 5,651 (62%) |
| Islam | 3,060 (34%) |
| No religion/Atheist/Agnostic | 345 (3.8%) |
| Primal, Animist, or Folk religion | 11 (0.1%) |
| Missing | 7 (<0.1%) |
| **Race and ethnicity** |  |
| African | 9,060 (100%) |
| Arab | 11 (0.1%) |
| Indian | 3 (<0.1%) |
| Missing | 2 (<0.1%) |
| ^1^n (%) | |

***Table S19b. Regression of volunteering on childhood predictors for Tanzania***

| Variable | Category | Risk ratio | 95% CI | Global p-value |
| --- | --- | --- | --- | --- |
| Relationship with mother (Ref: Very bad/Somewhat bad) | Very good/Somewhat good | 0.95 | (0.66, 1.36) | 0.809 |
| Relationship with father (Ref: Very bad/Somewhat bad) | Very good/Somewhat good | 1.28 | (0.96, 1.72) | <.001 |
| Parent marital status (Ref: Parents married) | No, divorced | 0.99 | (0.75, 1.32) | 0.015 |
|  | Never | 0.80 | (0.58, 1.11) |  |
|  | No, one or both of them had died | 1.32 | (0.97, 1.82) |  |
| Subjective financial status of family growing up (Ref: Got by) | Lived comfortably | 0.98 | (0.82, 1.16) | 0.899 |
|  | Found it difficult | 0.89 | (0.73, 1.07) |  |
|  | Found it very difficult | 0.98 | (0.75, 1.30) |  |
| Abuse (Ref: No) | Yes | 1.22 | (0.96, 1.55) | 0.01 |
| Outsider growing up (Ref: No) | Yes | 1.19 | (0.90, 1.55) | 0.129 |
| Self-rated health growing up (Ref: Good) | Excellent | 0.92 | (0.76, 1.12) | <.001 |
|  | Very good | 0.90 | (0.74, 1.09) |  |
|  | Fair | 0.89 | (0.70, 1.13) |  |
|  | Poor | 1.52 | (1.11, 2.11) |  |
| Immigration status (Ref: Born in this country) | Born in another country | 1.23 | (0.44, 3.45) | 0.955 |
| Age 12 religious service attendance (Ref: Never) | At least 1/week | 1.22 | (0.84, 1.79) | 0.126 |
|  | 1-3/month | 1.07 | (0.72, 1.60) |  |
|  | <1/month | 0.89 | (0.56, 1.42) |  |
| Age group (Ref: 1998-2005; current age: 18-24) | 1993-1998; age: 25-29 | 1.21 | (0.95, 1.54) | <.001 |
|  | 1983-1993; age: 30-39 | 1.30 | (1.05, 1.61) |  |
|  | 1973-1983; age 40-49 | 1.23 | (0.99, 1.54) |  |
|  | 1963-1973; age 50-59 | 1.35 | (1.05, 1.75) |  |
|  | 1953-1963; age 60-69 | 1.75 | (1.26, 2.44) |  |
|  | 1943-1953; age 70-79 | 0.82 | (0.43, 1.55) |  |
|  | 1943 or earlier; age 80+ | 0.97 | (0.42, 2.21) |  |
| Gender (Ref: Male) | Female | 0.63 | (0.55, 0.73) | <.001 |
| Religious affiliation at age 12 (Ref: No religion/Atheist/Agnostic) | Christianity | 1.00 | (0.64, 1.57) | <.001 |
|  | Islam | 0.78 | (0.49, 1.22) |  |
|  | Some other religion | 2.18 | (0.70, 6.86) |  |
| Race and ethnicity plurality (Ref: Majority) | Minority | 0.00 | (0.00, 0.00) | <.001 |

***Table S19c. Sensitivity to unmeasured confounding of childhood predictors in Tanzania***

| Variable | Category | E-value for Estimate | E-value for 95% CI |
| --- | --- | --- | --- |
| Relationship with mother (Ref: Very bad/Somewhat bad) | Very good/Somewhat good | 1.28 | 1.00 |
| Relationship with father (Ref: Very bad/Somewhat bad) | Very good/Somewhat good | 1.88 | 1.00 |
| Parent marital status (Ref: Parents married) | No, divorced | 1.09 | 1.00 |
|  | Never | 1.81 | 1.00 |
|  | No, one or both of them had died | 1.99 | 1.00 |
| Subjective financial status of family growing up (Ref: Got by) | Lived comfortably | 1.18 | 1.00 |
|  | Found it difficult | 1.51 | 1.00 |
|  | Found it very difficult | 1.14 | 1.00 |
| Abuse (Ref: No) | Yes | 1.75 | 1.00 |
| Outsider growing up (Ref: No) | Yes | 1.65 | 1.00 |
| Self-rated health growing up (Ref: Good) | Excellent | 1.38 | 1.00 |
|  | Very good | 1.47 | 1.00 |
|  | Fair | 1.50 | 1.00 |
|  | Poor | 2.42 | 1.45 |
| Immigration status (Ref: Born in this country) | Born in another country | 1.78 | 1.00 |
| Age 12 religious service attendance (Ref: Never) | At least 1/week | 1.74 | 1.00 |
|  | 1-3/month | 1.35 | 1.00 |
|  | <1/month | 1.50 | 1.00 |
| Age group (Ref: 1998-2005; current age: 18-24) | 1993-1998; age: 25-29 | 1.71 | 1.00 |
|  | 1983-1993; age: 30-39 | 1.93 | 1.28 |
|  | 1973-1983; age 40-49 | 1.77 | 1.00 |
|  | 1963-1973; age 50-59 | 2.04 | 1.26 |
|  | 1953-1963; age 60-69 | 2.91 | 1.83 |
|  | 1943-1953; age 70-79 | 1.75 | 1.00 |
|  | 1943 or earlier; age 80+ | 1.22 | 1.00 |
| Gender (Ref: Male) | Female | 2.55 | 2.08 |
| Religious affiliation at age 12 (Ref: No religion/Atheist/Agnostic) | Christianity | 1.07 | 1.00 |
|  | Some other religion | 3.81 | 1.00 |
| Race and ethnicity plurality (Ref: Majority) | Minority | 274,631.83 | 135,118.16 |
|  | Islam | 1.90 | 1.00 |

***Table S20a. Nationally representative descriptive statistics for Turkey***

| **Characteristic** | **N = 1,473**^1^ |
| --- | --- |
| **Relationship with mother** |  |
| Very good | 970 (66%) |
| Somewhat good | 401 (27%) |
| Somewhat bad | 48 (3.2%) |
| Very bad | 26 (1.8%) |
| Does not apply | 21 (1.4%) |
| Missing | 7 (0.5%) |
| **Relationship with father** |  |
| Very good | 795 (54%) |
| Somewhat good | 425 (29%) |
| Somewhat bad | 73 (5.0%) |
| Very bad | 95 (6.5%) |
| Does not apply | 60 (4.1%) |
| Missing | 25 (1.7%) |
| **Parent marital status** |  |
| Yes, married | 1,325 (90%) |
| No, divorced | 57 (3.9%) |
| Never married | 7 (0.5%) |
| No, one or both of them had died | 61 (4.1%) |
| Missing | 23 (1.5%) |
| **Subjective financial status of family growing up** |  |
| Lived comfortably | 498 (34%) |
| Got by | 647 (44%) |
| Found it difficult | 218 (15%) |
| Found it very difficult | 108 (7.3%) |
| Missing | 2 (0.1%) |
| **Abuse** |  |
| Yes | 158 (11%) |
| No | 1,290 (88%) |
| Missing | 25 (1.7%) |
| **Outsider growing up** |  |
| Yes | 157 (11%) |
| No | 1,306 (89%) |
| Missing | 9 (0.6%) |
| **Self-rated health growing up** |  |
| Excellent | 377 (26%) |
| Very good | 410 (28%) |
| Good | 419 (28%) |
| Fair | 220 (15%) |
| Poor | 47 (3.2%) |
| Missing | 0 (<0.1%) |
| **Immigration status** |  |
| Born in this country | 1,415 (96%) |
| Born in another country | 58 (4.0%) |
| Missing | 0 (0%) |
| **Age 12 religious service attendance** |  |
| At least 1/week | 609 (41%) |
| 1-3/month | 238 (16%) |
| <1/month | 225 (15%) |
| Never | 383 (26%) |
| Missing | 18 (1.2%) |
| **Age group** |  |
| 1998-2005; current age: 18-24 | 222 (15%) |
| 1993-1998; age: 25-29 | 152 (10%) |
| 1983-1993; age: 30-39 | 315 (21%) |
| 1973-1983; age 40-49 | 312 (21%) |
| 1963-1973; age 50-59 | 225 (15%) |
| 1953-1963; age 60-69 | 164 (11%) |
| 1943-1953; age 70-79 | 65 (4.4%) |
| 1943 or earlier; age 80+ | 18 (1.2%) |
| Missing | 0 (0%) |
| **Gender** |  |
| Male | 754 (51%) |
| Female | 719 (49%) |
| Other | 0 (0%) |
| Missing | 0 (0%) |
| **Religious affiliation at age 12** |  |
| Christianity | 1 (<0.1%) |
| Islam | 1,439 (98%) |
| Judaism | 1 (<0.1%) |
| No religion/Atheist/Agnostic | 13 (0.9%) |
| Missing | 19 (1.3%) |
| **Race and ethnicity** |  |
| Albanian | 8 (0.5%) |
| Arab | 51 (3.5%) |
| Armenian | 1 (<0.1%) |
| Azeri | 9 (0.6%) |
| Bosnian | 5 (0.3%) |
| Circassian | 19 (1.3%) |
| Georgian | 4 (0.3%) |
| Greek | 1 (<0.1%) |
| Kurdish/Zaza | 252 (17%) |
| Laz | 25 (1.7%) |
| Other | 58 (3.9%) |
| Turkish | 1,030 (70%) |
| Uyghur | 1 (<0.1%) |
| Missing | 9 (0.6%) |
| ^1^n (%) | |

***Table S20b. Regression of volunteering on childhood predictors for Turkey***

| Variable | Category | Risk ratio | 95% CI | Global p-value |
| --- | --- | --- | --- | --- |
| Relationship with mother (Ref: Very bad/Somewhat bad) | Very good/Somewhat good | 2.83 | (1.40, 5.67) | <.001 |
| Relationship with father (Ref: Very bad/Somewhat bad) | Very good/Somewhat good | 0.78 | (0.51, 1.18) | 0.001 |
| Parent marital status (Ref: Parents married) | No, divorced | 1.31 | (0.65, 2.61) | 0.985 |
|  | Never | 1.35 | (0.40, 4.53) |  |
|  | No, one or both of them had died | 1.31 | (0.55, 3.13) |  |
| Subjective financial status of family growing up (Ref: Got by) | Lived comfortably | 1.20 | (0.87, 1.63) | 0.927 |
|  | Found it difficult | 1.03 | (0.69, 1.54) |  |
|  | Found it very difficult | 0.86 | (0.42, 1.78) |  |
| Abuse (Ref: No) | Yes | 1.27 | (0.82, 1.99) | 0.252 |
| Outsider growing up (Ref: No) | Yes | 1.38 | (0.92, 2.05) | 0.017 |
| Self-rated health growing up (Ref: Good) | Excellent | 1.08 | (0.78, 1.52) | 1 |
|  | Very good | 1.05 | (0.72, 1.53) |  |
|  | Fair | 0.90 | (0.55, 1.47) |  |
|  | Poor | 0.79 | (0.31, 2.00) |  |
| Immigration status (Ref: Born in this country) | Born in another country | 1.58 | (0.88, 2.87) | 0.022 |
| Age 12 religious service attendance (Ref: Never) | At least 1/week | 1.13 | (0.77, 1.64) | 0.157 |
|  | 1-3/month | 1.19 | (0.76, 1.84) |  |
|  | <1/month | 1.52 | (1.00, 2.30) |  |
| Age group (Ref: 1998-2005; current age: 18-24) | 1993-1998; age: 25-29 | 1.03 | (0.67, 1.60) | 0.329 |
|  | 1983-1993; age: 30-39 | 0.91 | (0.62, 1.34) |  |
|  | 1973-1983; age 40-49 | 0.86 | (0.58, 1.28) |  |
|  | 1963-1973; age 50-59 | 1.09 | (0.68, 1.76) |  |
|  | 1953-1963; age 60-69 | 0.43 | (0.19, 0.96) |  |
|  | 1943-1953; age 70-79 | 0.33 | (0.07, 1.44) |  |
|  | 1943 or earlier; age 80+ | 0.41 | (0.06, 2.91) |  |
| Gender (Ref: Male) | Female | 0.78 | (0.57, 1.06) | 0.015 |
| Religious affiliation at age 12 (Ref: Islam) | Some other religion | 2.01 | (1.00, 4.05) | 0.001 |
| Race and ethnicity plurality (Ref: Majority) | Minority | 1.05 | (0.79, 1.41) | 0.975 |

***Table S20c. Sensitivity to unmeasured confounding of childhood predictors in Turkey***

| Variable | Category | E-value for Estimate | E-value for 95% CI |
| --- | --- | --- | --- |
| Relationship with mother (Ref: Very bad/Somewhat bad) | Very good/Somewhat good | 5.08 | 2.15 |
| Relationship with father (Ref: Very bad/Somewhat bad) | Very good/Somewhat good | 1.89 | 1.00 |
| Parent marital status (Ref: Parents married) | No, divorced | 1.94 | 1.00 |
|  | Never | 2.04 | 1.00 |
|  | No, one or both of them had died | 1.95 | 1.00 |
| Subjective financial status of family growing up (Ref: Got by) | Lived comfortably | 1.68 | 1.00 |
|  | Found it difficult | 1.22 | 1.00 |
|  | Found it very difficult | 1.59 | 1.00 |
| Abuse (Ref: No) | Yes | 1.87 | 1.00 |
| Outsider growing up (Ref: No) | Yes | 2.09 | 1.00 |
| Self-rated health growing up (Ref: Good) | Excellent | 1.40 | 1.00 |
|  | Very good | 1.27 | 1.00 |
|  | Fair | 1.46 | 1.00 |
|  | Poor | 1.86 | 1.00 |
| Immigration status (Ref: Born in this country) | Born in another country | 2.56 | 1.00 |
| Age 12 religious service attendance (Ref: Never) | At least 1/week | 1.50 | 1.00 |
|  | 1-3/month | 1.64 | 1.00 |
|  | <1/month | 2.40 | 1.00 |
| Age group (Ref: 1998-2005; current age: 18-24) | 1993-1998; age: 25-29 | 1.22 | 1.00 |
|  | 1983-1993; age: 30-39 | 1.42 | 1.00 |
|  | 1973-1983; age 40-49 | 1.59 | 1.00 |
|  | 1963-1973; age 50-59 | 1.40 | 1.00 |
|  | 1953-1963; age 60-69 | 4.13 | 1.26 |
|  | 1943-1953; age 70-79 | 5.61 | 1.00 |
|  | 1943 or earlier; age 80+ | 4.25 | 1.00 |
| Gender (Ref: Male) | Female | 1.89 | 1.00 |
| Religious affiliation at age 12 (Ref: Islam) | Some other religion | 3.44 | 1.01 |
| Race and ethnicity plurality (Ref: Majority) | Minority | 1.29 | 1.00 |

***Table S21a. Nationally representative descriptive statistics for United Kingdom***

| **Characteristic** | **N = 5,368**^1^ |
| --- | --- |
| **Relationship with mother** |  |
| Very good | 3,435 (64%) |
| Somewhat good | 1,338 (25%) |
| Somewhat bad | 325 (6.1%) |
| Very bad | 150 (2.8%) |
| Does not apply | 92 (1.7%) |
| Missing | 27 (0.5%) |
| **Relationship with father** |  |
| Very good | 2,907 (54%) |
| Somewhat good | 1,383 (26%) |
| Somewhat bad | 407 (7.6%) |
| Very bad | 321 (6.0%) |
| Does not apply | 321 (6.0%) |
| Missing | 29 (0.5%) |
| **Parent marital status** |  |
| Yes, married | 4,343 (81%) |
| No, divorced | 481 (9.0%) |
| Never married | 315 (5.9%) |
| No, one or both of them had died | 154 (2.9%) |
| Missing | 75 (1.4%) |
| **Subjective financial status of family growing up** |  |
| Lived comfortably | 2,552 (48%) |
| Got by | 1,933 (36%) |
| Found it difficult | 632 (12%) |
| Found it very difficult | 230 (4.3%) |
| Missing | 22 (0.4%) |
| **Abuse** |  |
| Yes | 864 (16%) |
| No | 4,455 (83%) |
| Missing | 49 (0.9%) |
| **Outsider growing up** |  |
| Yes | 1,017 (19%) |
| No | 4,308 (80%) |
| Missing | 43 (0.8%) |
| **Self-rated health growing up** |  |
| Excellent | 2,154 (40%) |
| Very good | 1,736 (32%) |
| Good | 995 (19%) |
| Fair | 332 (6.2%) |
| Poor | 130 (2.4%) |
| Missing | 20 (0.4%) |
| **Immigration status** |  |
| Born in this country | 4,659 (87%) |
| Born in another country | 682 (13%) |
| Missing | 27 (0.5%) |
| **Age 12 religious service attendance** |  |
| At least 1/week | 1,732 (32%) |
| 1-3/month | 733 (14%) |
| <1/month | 903 (17%) |
| Never | 1,972 (37%) |
| Missing | 28 (0.5%) |
| **Age group** |  |
| 1998-2005; current age: 18-24 | 490 (9.1%) |
| 1993-1998; age: 25-29 | 391 (7.3%) |
| 1983-1993; age: 30-39 | 946 (18%) |
| 1973-1983; age 40-49 | 827 (15%) |
| 1963-1973; age 50-59 | 949 (18%) |
| 1953-1963; age 60-69 | 889 (17%) |

| 1943-1953; age 70-79 | 711 (13%) |
| --- | --- |

| 1943 or earlier; age 80+ | 163 (3.0%) |
| --- | --- |
| Missing | 1 (<0.1%) |
| **Gender** |  |
| Male | 2,557 (48%) |
| Female | 2,789 (52%) |
| Other | 14 (0.3%) |
| Missing | 9 (0.2%) |
| **Religious affiliation at age 12** |  |
| Baha’i | 5 (<0.1%) |
| Buddhism | 15 (0.3%) |
| Christianity | 3,461 (64%) |
| Confucianism | 3 (<0.1%) |
| Hinduism | 88 (1.6%) |
| Islam | 230 (4.3%) |
| Jainism | 0 (<0.1%) |
| Judaism | 59 (1.1%) |
| No religion/Atheist/Agnostic | 1,409 (26%) |
| Primal, Animist, or Folk religion | 22 (0.4%) |
| Sikhism | 30 (0.6%) |
| Some other religion | 24 (0.5%) |
| Taoism | 2 (<0.1%) |
| Missing | 21 (0.4%) |
| **Race and ethnicity** |  |
| Asian | 426 (7.9%) |
| Black | 152 (2.8%) |
| Other | 96 (1.8%) |
| White | 4,647 (87%) |
| Missing | 47 (0.9%) |
| ^1^n (%) | |

***Table S21b. Regression of volunteering on childhood predictors for United Kingdom***

| Variable | Category | Risk ratio | 95% CI | Global p-value |
| --- | --- | --- | --- | --- |
| Relationship with mother (Ref: Very bad/Somewhat bad) | Very good/Somewhat good | 1.14 | (0.92, 1.40) | 0.001 |
| Relationship with father (Ref: Very bad/Somewhat bad) | Very good/Somewhat good | 1.16 | (0.96, 1.40) | <.001 |
| Parent marital status (Ref: Parents married) | No, divorced | 0.94 | (0.75, 1.18) | <.001 |
|  | Never | 0.66 | (0.47, 0.94) |  |
|  | No, one or both of them had died | 1.27 | (0.91, 1.76) |  |
| Subjective financial status of family growing up (Ref: Got by) | Lived comfortably | 1.22 | (1.07, 1.38) | <.001 |
|  | Found it difficult | 1.01 | (0.82, 1.23) |  |
|  | Found it very difficult | 0.87 | (0.60, 1.25) |  |
| Abuse (Ref: No) | Yes | 1.15 | (1.00, 1.32) | 0.001 |
| Outsider growing up (Ref: No) | Yes | 1.23 | (1.07, 1.41) | <.001 |
| Self-rated health growing up (Ref: Good) | Excellent | 1.02 | (0.86, 1.20) | 1 |
|  | Very good | 1.03 | (0.87, 1.22) |  |
|  | Fair | 0.94 | (0.70, 1.26) |  |
|  | Poor | 1.09 | (0.75, 1.60) |  |
| Immigration status (Ref: Born in this country) | Born in another country | 0.99 | (0.83, 1.17) | 0.999 |
| Age 12 religious service attendance (Ref: Never) | At least 1/week | 1.75 | (1.49, 2.05) | <.001 |
|  | 1-3/month | 1.68 | (1.39, 2.05) |  |
|  | <1/month | 1.39 | (1.15, 1.67) |  |
| Age group (Ref: 1998-2005; current age: 18-24) | 1993-1998; age: 25-29 | 0.84 | (0.65, 1.10) | <.001 |
|  | 1983-1993; age: 30-39 | 0.78 | (0.62, 0.98) |  |
|  | 1973-1983; age 40-49 | 0.76 | (0.60, 0.97) |  |
|  | 1963-1973; age 50-59 | 0.73 | (0.57, 0.93) |  |
|  | 1953-1963; age 60-69 | 0.55 | (0.43, 0.71) |  |
|  | 1943-1953; age 70-79 | 0.63 | (0.48, 0.81) |  |
|  | 1943 or earlier; age 80+ | 0.55 | (0.38, 0.82) |  |
| Gender (Ref: Male) | Female | 0.88 | (0.79, 0.99) | <.001 |
|  | Other | 0.00 | (0.00, 0.00) |  |
| Religious affiliation at age 12 (Ref: No religion/Atheist/Agnostic) | Christianity | 1.05 | (0.90, 1.23) | 0.249 |
|  | Islam | 0.77 | (0.55, 1.09) |  |
|  | Some other religion | 0.99 | (0.73, 1.34) |  |
| Race and ethnicity plurality (Ref: Majority) | Minority | 1.13 | (0.93, 1.37) | 0.146 |

***Table S21c. Sensitivity to unmeasured confounding of childhood predictors in United Kingdom***

| Variable | Category | E-value for Estimate | E-value for 95% CI |
| --- | --- | --- | --- |
| Relationship with mother (Ref: Very bad/Somewhat bad) | Very good/Somewhat good | 1.53 | 1.00 |
| Relationship with father (Ref: Very bad/Somewhat bad) | Very good/Somewhat good | 1.59 | 1.00 |
| Parent marital status (Ref: Parents married) | No, divorced | 1.33 | 1.00 |
|  | Never | 2.38 | 1.34 |
|  | No, one or both of them had died | 1.85 | 1.00 |
| Subjective financial status of family growing up (Ref: Got by) | Lived comfortably | 1.74 | 1.36 |
|  | Found it difficult | 1.08 | 1.00 |
|  | Found it very difficult | 1.57 | 1.00 |
| Abuse (Ref: No) | Yes | 1.57 | 1.00 |
| Outsider growing up (Ref: No) | Yes | 1.76 | 1.35 |
| Self-rated health growing up (Ref: Good) | Excellent | 1.15 | 1.00 |
|  | Very good | 1.22 | 1.00 |
|  | Fair | 1.33 | 1.00 |
|  | Poor | 1.42 | 1.00 |
| Immigration status (Ref: Born in this country) | Born in another country | 1.13 | 1.00 |
| Age 12 religious service attendance (Ref: Never) | At least 1/week | 2.89 | 2.33 |
|  | 1-3/month | 2.77 | 2.13 |
|  | <1/month | 2.12 | 1.57 |
| Age group (Ref: 1998-2005; current age: 18-24) | 1993-1998; age: 25-29 | 1.65 | 1.00 |
|  | 1983-1993; age: 30-39 | 1.89 | 1.19 |
|  | 1973-1983; age 40-49 | 1.95 | 1.21 |
|  | 1963-1973; age 50-59 | 2.11 | 1.37 |
|  | 1953-1963; age 60-69 | 3.02 | 2.15 |
|  | 1943-1953; age 70-79 | 2.58 | 1.77 |
|  | 1943 or earlier; age 80+ | 2.99 | 1.75 |
| Gender (Ref: Male) | Female | 1.52 | 1.13 |
|  | Other | 851,450.21 | 388,218.28 |
| Religious affiliation at age 12 (Ref: No religion/Atheist/Agnostic) | Christianity | 1.27 | 1.00 |
|  | Some other religion | 1.10 | 1.00 |
| Race and ethnicity plurality (Ref: Majority) | Minority | 1.50 | 1.00 |
|  | Islam | 1.92 | 1.00 |

***Table S22a. Nationally representative descriptive statistics for United States***

| **Characteristic** | **N = 38,312**^1^ |
| --- | --- |
| **Relationship with mother** |  |
| Very good | 20,590 (54%) |
| Somewhat good | 11,525 (30%) |
| Somewhat bad | 3,523 (9.2%) |
| Very bad | 1,874 (4.9%) |
| Does not apply | 694 (1.8%) |
| Missing | 106 (0.3%) |
| **Relationship with father** |  |
| Very good | 15,313 (40%) |
| Somewhat good | 12,665 (33%) |
| Somewhat bad | 4,879 (13%) |
| Very bad | 2,604 (6.8%) |
| Does not apply | 2,811 (7.3%) |
| Missing | 38 (0.1%) |
| **Parent marital status** |  |
| Yes, married | 27,415 (72%) |
| No, divorced | 6,325 (17%) |
| Never married | 3,048 (8.0%) |
| No, one or both of them had died | 1,024 (2.7%) |
| Missing | 500 (1.3%) |
| **Subjective financial status of family growing up** |  |
| Lived comfortably | 15,116 (39%) |
| Got by | 15,682 (41%) |
| Found it difficult | 5,152 (13%) |
| Found it very difficult | 2,342 (6.1%) |
| Missing | 19 (<0.1%) |
| **Abuse** |  |
| Yes | 10,026 (26%) |
| No | 28,045 (73%) |
| Missing | 242 (0.6%) |
| **Outsider growing up** |  |
| Yes | 10,185 (27%) |
| No | 27,714 (72%) |
| Missing | 413 (1.1%) |
| **Self-rated health growing up** |  |
| Excellent | 16,866 (44%) |
| Very good | 12,108 (32%) |
| Good | 6,444 (17%) |
| Fair | 2,303 (6.0%) |
| Poor | 520 (1.4%) |
| Missing | 71 (0.2%) |
| **Immigration status** |  |
| Born in this country | 34,865 (91%) |
| Born in another country | 3,020 (7.9%) |
| Missing | 427 (1.1%) |
| **Age 12 religious service attendance** |  |
| At least 1/week | 18,609 (49%) |
| 1-3/month | 6,644 (17%) |
| <1/month | 5,829 (15%) |
| Never | 7,085 (18%) |
| Missing | 145 (0.4%) |
| **Age group** |  |
| 1998-2005; current age: 18-24 | 2,682 (7.0%) |
| 1993-1998; age: 25-29 | 3,540 (9.2%) |
| 1983-1993; age: 30-39 | 7,284 (19%) |
| 1973-1983; age 40-49 | 5,649 (15%) |
| 1963-1973; age 50-59 | 6,745 (18%) |
| 1953-1963; age 60-69 | 6,832 (18%) |
| 1943-1953; age 70-79 | 4,054 (11%) |
| 1943 or earlier; age 80+ | 1,525 (4.0%) |
| Missing | 0 (0%) |
| **Gender** |  |
| Male | 18,222 (48%) |
| Female | 19,562 (51%) |
| Other | 392 (1.0%) |
| Missing | 136 (0.4%) |
| **Religious affiliation at age 12** |  |
| Baha’i | 4 (<0.1%) |
| Buddhism | 172 (0.4%) |
| Christianity | 30,444 (79%) |
| Confucianism | 8 (<0.1%) |
| Hinduism | 203 (0.5%) |
| Islam | 220 (0.6%) |
| Jainism | 18 (<0.1%) |
| Judaism | 787 (2.1%) |
| No religion/Atheist/Agnostic | 5,845 (15%) |
| Primal, Animist, or Folk religion | 67 (0.2%) |
| Shinto | 6 (<0.1%) |
| Sikhism | 47 (0.1%) |
| Some other religion | 359 (0.9%) |
| Taoism | 17 (<0.1%) |
| Missing | 115 (0.3%) |
| **Race and ethnicity** |  |
| Asian | 2,466 (6.4%) |
| Black | 4,501 (12%) |
| Hispanic | 6,724 (18%) |
| Other | 997 (2.6%) |
| White | 23,605 (62%) |
| Missing | 20 (<0.1%) |
| ^1^n (%) | |

***Table S22b. Regression of volunteering on childhood predictors for United States***

| Variable | Category | Risk ratio | 95% CI | Global p-value |
| --- | --- | --- | --- | --- |
| Relationship with mother (Ref: Very bad/Somewhat bad) | Very good/Somewhat good | 0.93 | (0.83, 1.06) | 0.006 |
| Relationship with father (Ref: Very bad/Somewhat bad) | Very good/Somewhat good | 1.12 | (1.00, 1.25) | <.001 |
| Parent marital status (Ref: Parents married) | No, divorced | 0.82 | (0.73, 0.92) | <.001 |
|  | Never | 0.95 | (0.74, 1.24) |  |
|  | No, one or both of them had died | 0.85 | (0.66, 1.10) |  |
| Subjective financial status of family growing up (Ref: Got by) | Lived comfortably | 1.04 | (0.97, 1.11) | 0.099 |
|  | Found it difficult | 1.06 | (0.94, 1.20) |  |
|  | Found it very difficult | 1.22 | (1.01, 1.48) |  |
| Abuse (Ref: No) | Yes | 1.11 | (1.01, 1.21) | <.001 |
| Outsider growing up (Ref: No) | Yes | 0.93 | (0.84, 1.03) | 0.035 |
| Self-rated health growing up (Ref: Good) | Excellent | 1.22 | (1.09, 1.37) | <.001 |
|  | Very good | 1.13 | (1.00, 1.28) |  |
|  | Fair | 0.93 | (0.73, 1.19) |  |
|  | Poor | 0.99 | (0.68, 1.43) |  |
| Immigration status (Ref: Born in this country) | Born in another country | 0.81 | (0.68, 0.96) | <.001 |
| Age 12 religious service attendance (Ref: Never) | At least 1/week | 1.55 | (1.36, 1.78) | <.001 |
|  | 1-3/month | 1.31 | (1.13, 1.53) |  |
|  | <1/month | 1.20 | (1.02, 1.40) |  |
| Age group (Ref: 1998-2005; current age: 18-24) | 1993-1998; age: 25-29 | 0.76 | (0.57, 1.01) | <.001 |
|  | 1983-1993; age: 30-39 | 0.74 | (0.58, 0.95) |  |
|  | 1973-1983; age 40-49 | 0.92 | (0.73, 1.16) |  |
|  | 1963-1973; age 50-59 | 0.82 | (0.65, 1.04) |  |
|  | 1953-1963; age 60-69 | 0.86 | (0.68, 1.08) |  |
|  | 1943-1953; age 70-79 | 0.90 | (0.71, 1.13) |  |
|  | 1943 or earlier; age 80+ | 0.75 | (0.58, 0.97) |  |
| Gender (Ref: Male) | Female | 1.07 | (1.00, 1.15) | 0.026 |
|  | Other | 1.04 | (0.63, 1.70) |  |
| Religious affiliation at age 12 (Ref: No religion/Atheist/Agnostic) | Christianity | 0.94 | (0.81, 1.09) | 0.304 |
|  | Judaism | 1.03 | (0.85, 1.25) |  |
|  | Some other religion | 1.14 | (0.85, 1.51) |  |
| Race and ethnicity plurality (Ref: Majority) | Minority | 0.87 | (0.79, 0.95) | <.001 |

***Table S22c. Sensitivity to unmeasured confounding of childhood predictors in United States***

| Variable | Category | E-value for Estimate | E-value for 95% CI |
| --- | --- | --- | --- |
| Relationship with mother (Ref: Very bad/Somewhat bad) | Very good/Somewhat good | 1.34 | 1.00 |
| Relationship with father (Ref: Very bad/Somewhat bad) | Very good/Somewhat good | 1.49 | 1.07 |
| Parent marital status (Ref: Parents married) | No, divorced | 1.73 | 1.40 |
|  | Never | 1.27 | 1.00 |
|  | No, one or both of them had died | 1.62 | 1.00 |
| Subjective financial status of family growing up (Ref: Got by) | Lived comfortably | 1.23 | 1.00 |
|  | Found it difficult | 1.32 | 1.00 |
|  | Found it very difficult | 1.74 | 1.08 |
| Abuse (Ref: No) | Yes | 1.44 | 1.12 |
| Outsider growing up (Ref: No) | Yes | 1.36 | 1.00 |
| Self-rated health growing up (Ref: Good) | Excellent | 1.74 | 1.40 |
|  | Very good | 1.52 | 1.05 |
|  | Fair | 1.36 | 1.00 |
|  | Poor | 1.14 | 1.00 |
| Immigration status (Ref: Born in this country) | Born in another country | 1.77 | 1.24 |
| Age 12 religious service attendance (Ref: Never) | At least 1/week | 2.48 | 2.05 |
|  | 1-3/month | 1.96 | 1.52 |
|  | <1/month | 1.67 | 1.14 |
| Age group (Ref: 1998-2005; current age: 18-24) | 1993-1998; age: 25-29 | 1.97 | 1.00 |
|  | 1983-1993; age: 30-39 | 2.02 | 1.28 |
|  | 1973-1983; age 40-49 | 1.40 | 1.00 |
|  | 1963-1973; age 50-59 | 1.73 | 1.00 |
|  | 1953-1963; age 60-69 | 1.60 | 1.00 |
|  | 1943-1953; age 70-79 | 1.47 | 1.00 |
|  | 1943 or earlier; age 80+ | 1.99 | 1.22 |
| Gender (Ref: Male) | Female | 1.35 | 1.05 |
|  | Other | 1.24 | 1.00 |
| Religious affiliation at age 12 (Ref: No religion/Atheist/Agnostic) | Christianity | 1.32 | 1.00 |
|  | Some other religion | 1.52 | 1.00 |
| Race and ethnicity plurality (Ref: Majority) | Minority | 1.57 | 1.30 |
|  | Judaism | 1.20 | 1.00 |

***Table S23. Population weighted meta-analysis of regression results***

| Variable | Category | RR | 95% CI |
| --- | --- | --- | --- |
| Relationship with mother | (Ref: Very bad/somewhat bad) |  |  |
|  | Very good/somewhat good | 1.10 | (0.97,1.26) |
| Relationship with father | (Ref: Very bad/somewhat bad) |  |  |
|  | Very good/somewhat good | 1.19 | (1.06,1.33) |
| Parent marital status | (Ref: Parents married) |  |  |
|  | No, divorced | 0.95 | (0.86,1.05) |
|  | Single, never married | 1.02 | (0.94,1.11) |
|  | No, one or both of them had died | 0.99 | (0.92,1.07) |
| Subjective financial status of family growing up | (Ref: Got by) |  |  |
|  | Lived comfortably | 1.11 | (1.06,1.17) |
|  | Found it difficult | 1.00 | (0.95,1.06) |
|  | Found it very difficult | 1.01 | (0.93,1.10) |
| Abuse | (Ref: No) |  |  |
|  | Yes | 1.15 | (1.10,1.21) |
| Outsider growing up | (Ref: No) |  |  |
|  | Yes | 1.22 | (1.16,1.28) |
| Self-rated health growing up | (Ref: Good) |  |  |
|  | Excellent | 1.10 | (1.04,1.17) |
|  | Very good | 1.11 | (1.05,1.18) |
|  | Fair | 1.10 | (1.03,1.17) |
|  | Poor | 1.16 | (1.04,1.30) |
| Immigration status | (Ref: Born in this country) |  |  |
|  | Born in another country | 0.61 | (0.49,0.76) |
| Age 12 religious service attendance | (Ref: Never) |  |  |
|  | At least 1/week | 1.53 | (1.42,1.65) |
|  | 1-3/month | 1.45 | (1.34,1.57) |
|  | <1/month | 1.26 | (1.16,1.37) |
| Year of birth | (Ref: 1998-2005; age 18-24) |  |  |
|  | 1993-1998; age 25-29 | 0.96 | (0.90,1.02) |
|  | 1983-1993; age 30-39 | 0.96 | (0.91,1.02) |
|  | 1973-1983; age 40-49 | 1.00 | (0.95,1.06) |
|  | 1963-1973; age 50-59 | 0.95 | (0.89,1.01) |
|  | 1953-1963; age 60-69 | 0.94 | (0.87,1.02) |
|  | 1943-1953; age 70-79 | 0.88 | (0.78,0.99) |
|  | 1943 or earlier; age 80+ | 0.60 | (0.46,0.77) |
| Gender | (Ref: Male) |  |  |
|  | Female | 0.86 | (0.83,0.89) |
|  | Other | 0.45 | (0.35,0.57) |

***Table S24. Population weighted meta-analysis of E-values***

| Variable | Category | *E*-value for Estimate | *E*-value for 95% CI |
| --- | --- | --- | --- |
| Relationship with mother | (Ref: Very bad/somewhat bad) |  |  |
|  | Very good/somewhat good | 1.44 | 1.00 |
| Relationship with father | (Ref: Very bad/somewhat bad) |  |  |
|  | Very good/somewhat good | 1.66 | 1.31 |
| Parent marital status | (Ref: Parents married) |  |  |
|  | No, divorced | 1.30 | 1.00 |
|  | Single, never married | 1.17 | 1.00 |
|  | No, one or both of them had died | 1.10 | 1.00 |
| Subjective financial status of family growing up | (Ref: Got by) |  |  |
|  | Lived comfortably | 1.46 | 1.32 |
|  | Found it difficult | 1.03 | 1.00 |
|  | Found it very difficult | 1.12 | 1.00 |
| Abuse | (Ref: No) |  |  |
|  | Yes | 1.57 | 1.42 |
| Outsider growing up | (Ref: No) |  |  |
|  | Yes | 1.74 | 1.58 |
| Self-rated health growing up | (Ref: Good) |  |  |
|  | Excellent | 1.44 | 1.24 |
|  | Very good | 1.47 | 1.30 |
|  | Fair | 1.43 | 1.21 |
|  | Poor | 1.59 | 1.23 |
| Immigration status | (Ref: Born in this country) |  |  |
|  | Born in another country | 2.68 | 1.97 |
| Age 12 religious service attendance | (Ref: Never) |  |  |
|  | At least 1/week | 2.43 | 2.19 |
|  | 1-3/month | 2.26 | 2.02 |
|  | <1/month | 1.83 | 1.59 |
| Year of birth | (Ref: 1998-2005; age 18-24) |  |  |
|  | 1993-1998; age 25-29 | 1.24 | 1.00 |
|  | 1983-1993; age 30-39 | 1.24 | 1.00 |
|  | 1973-1983; age 40-49 | 1.03 | 1.00 |
|  | 1963-1973; age 50-59 | 1.29 | 1.00 |
|  | 1953-1963; age 60-69 | 1.32 | 1.00 |
|  | 1943-1953; age 70-79 | 1.53 | 1.09 |
|  | 1943 or earlier; age 80+ | 2.74 | 1.93 |
| Gender | (Ref: Male) |  |  |
|  | Female | 1.61 | 1.51 |
|  | Other | 3.91 | 2.91 |


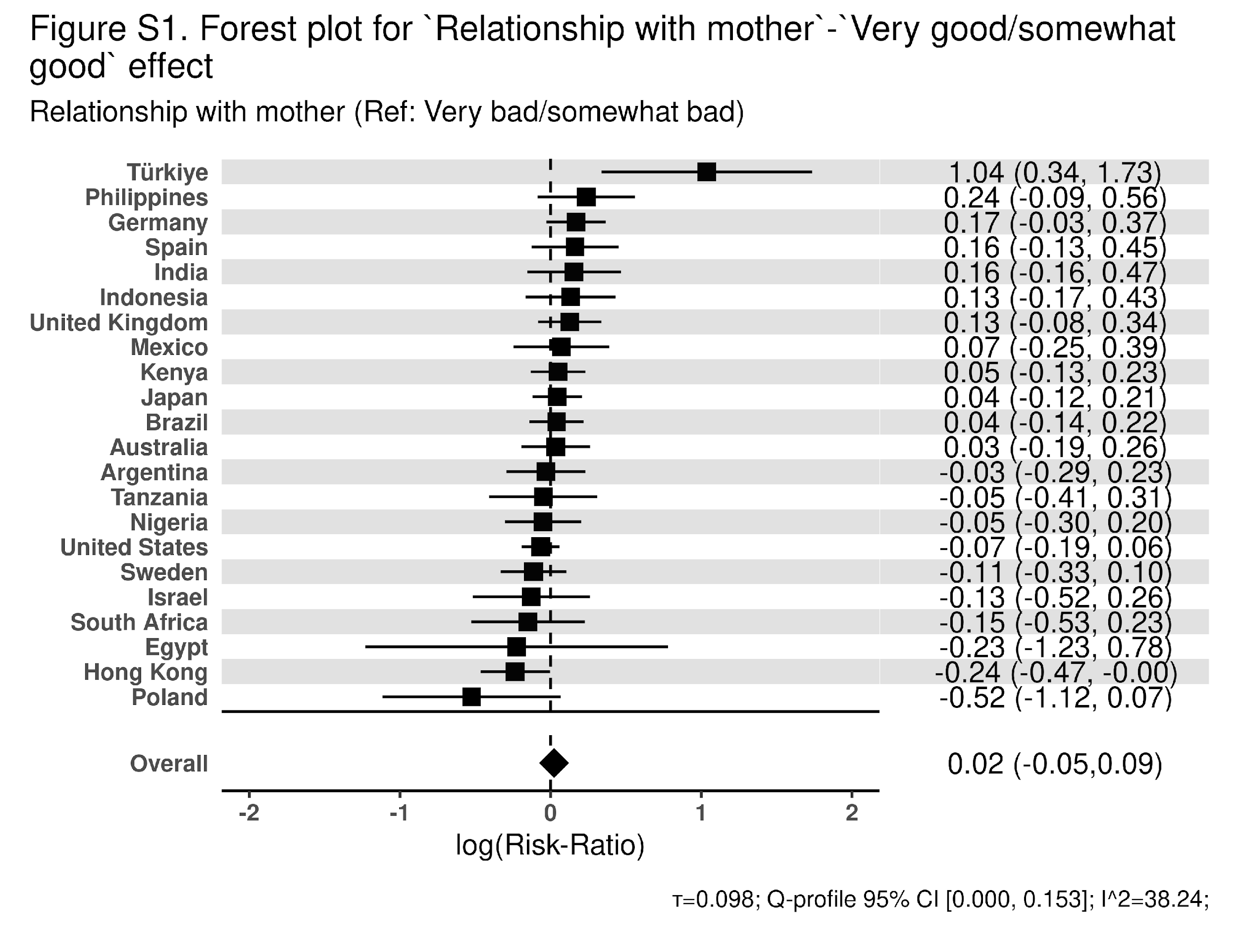

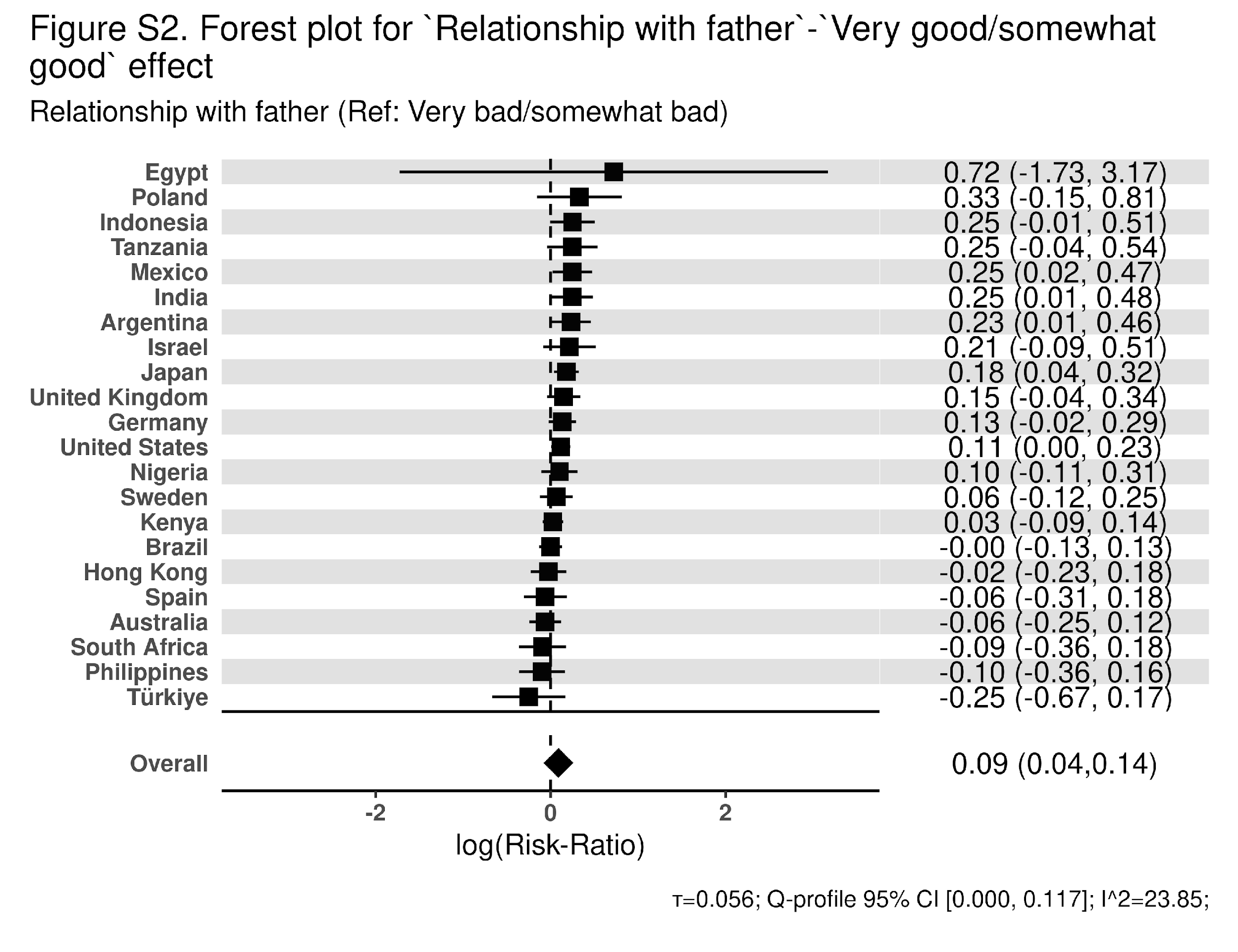

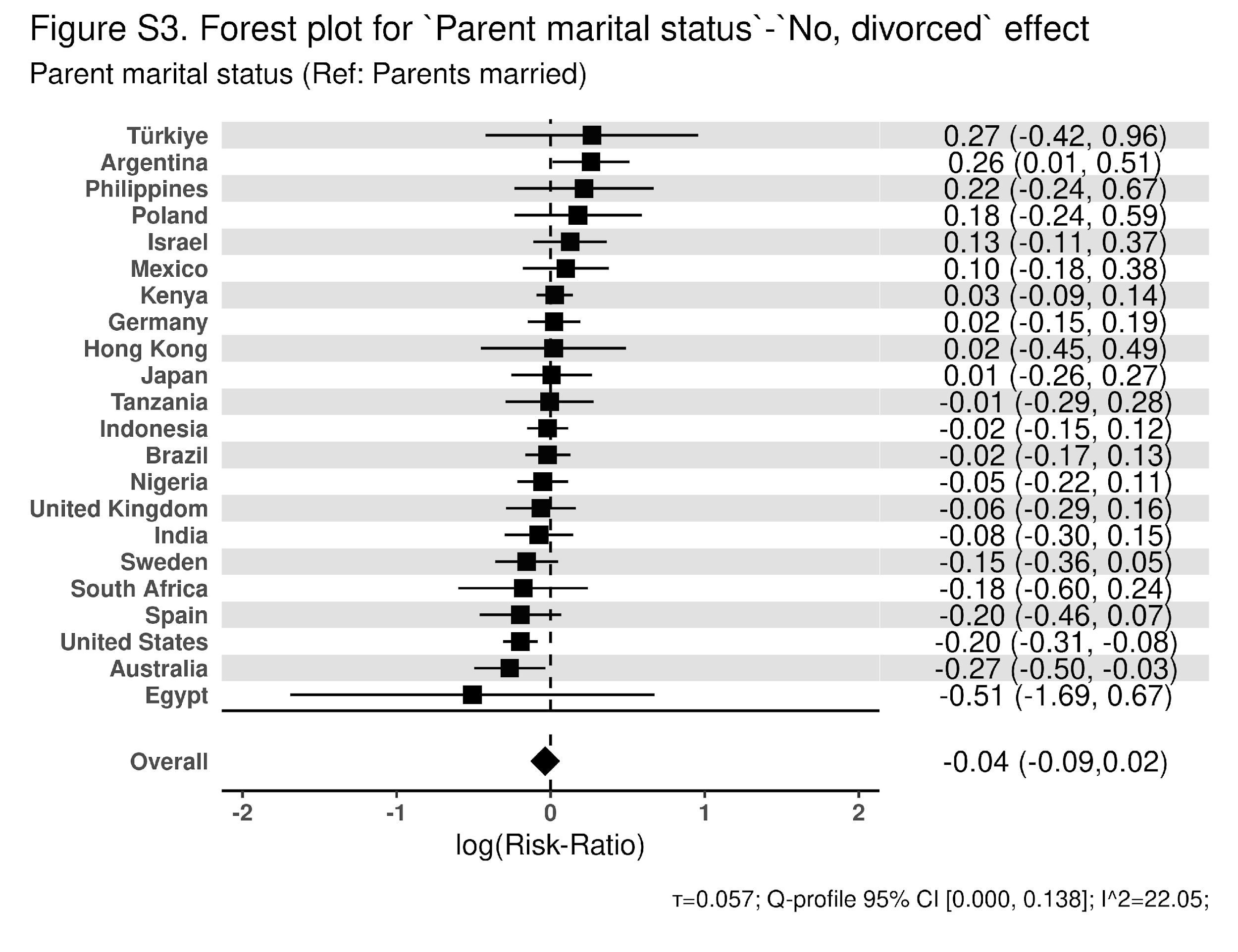

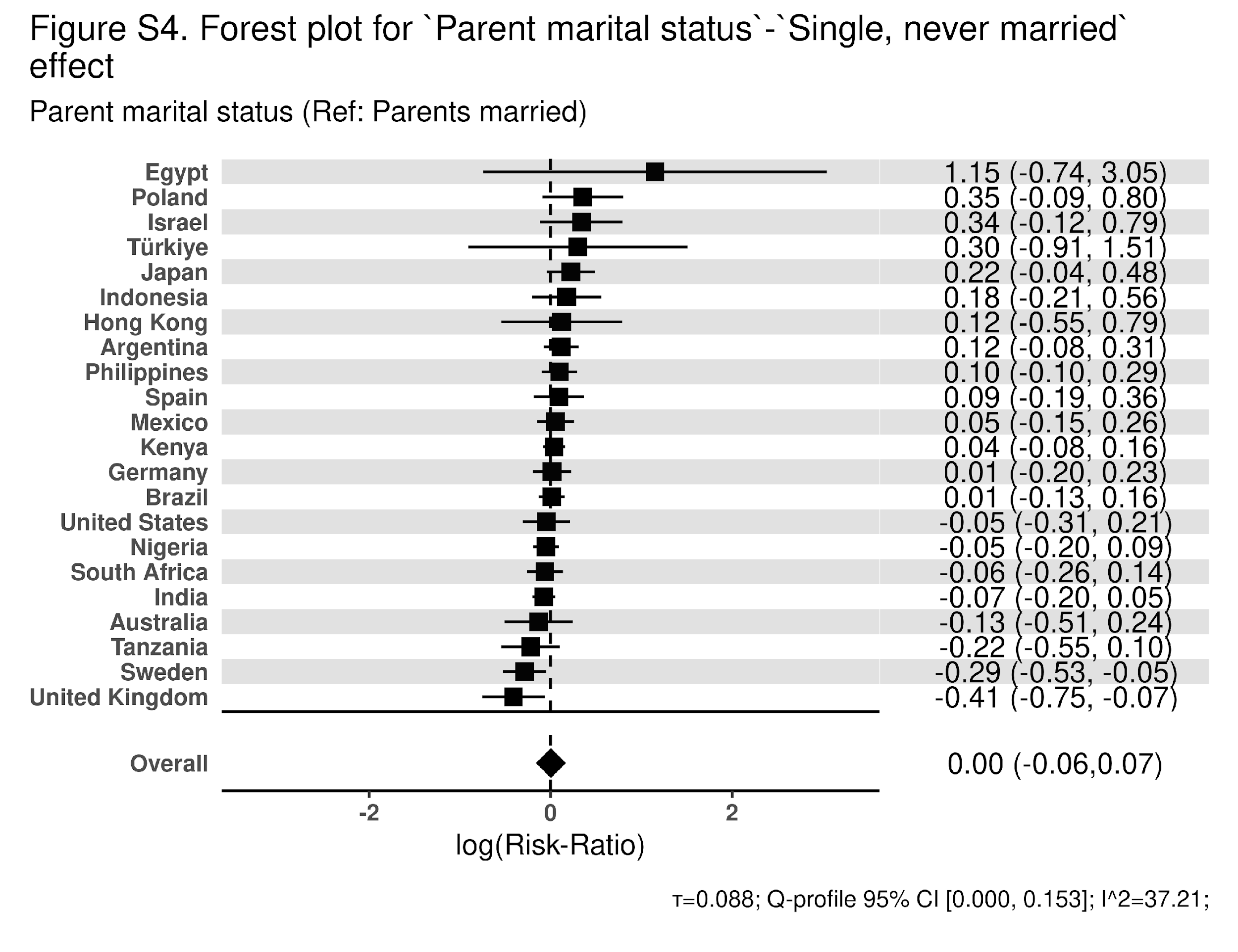

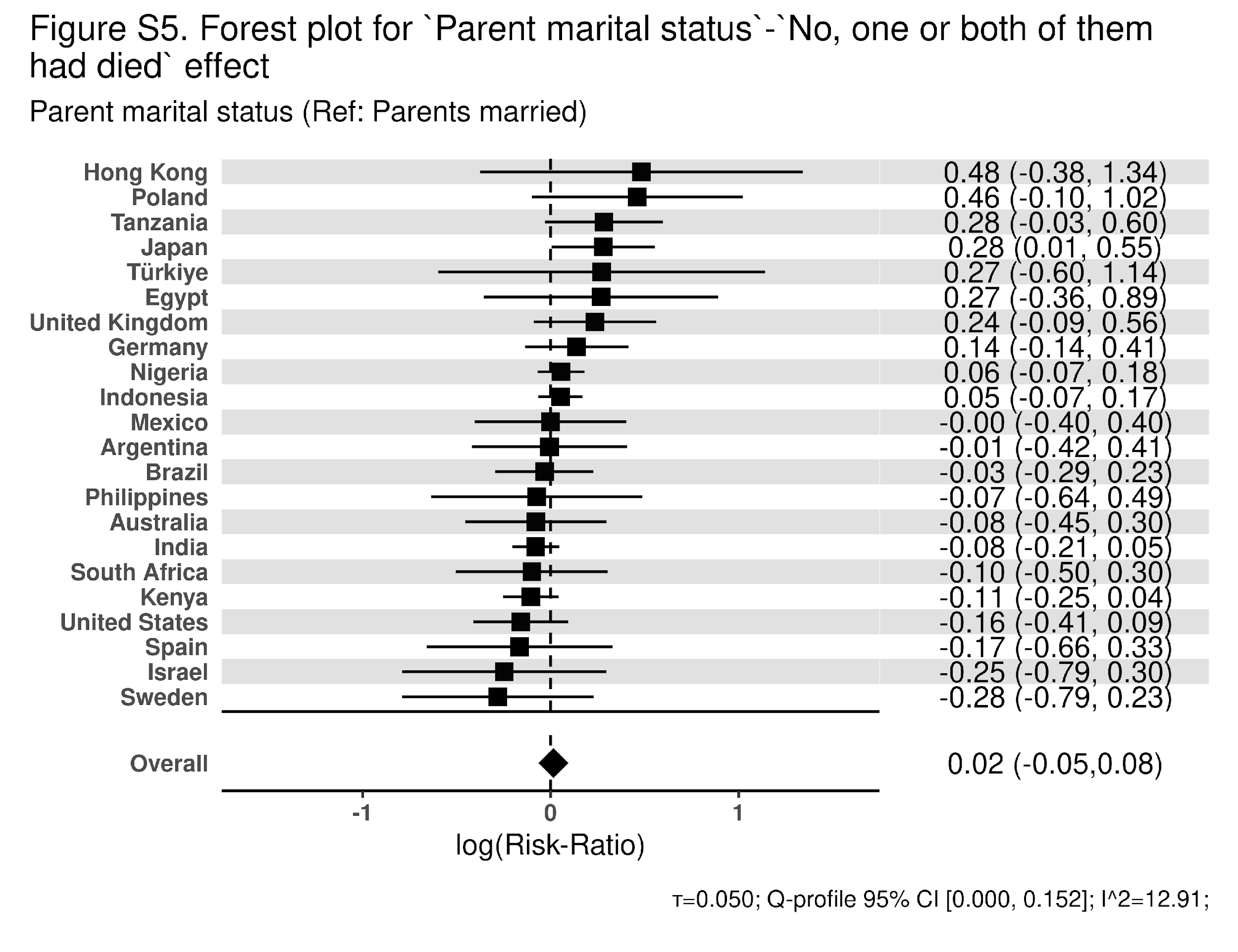

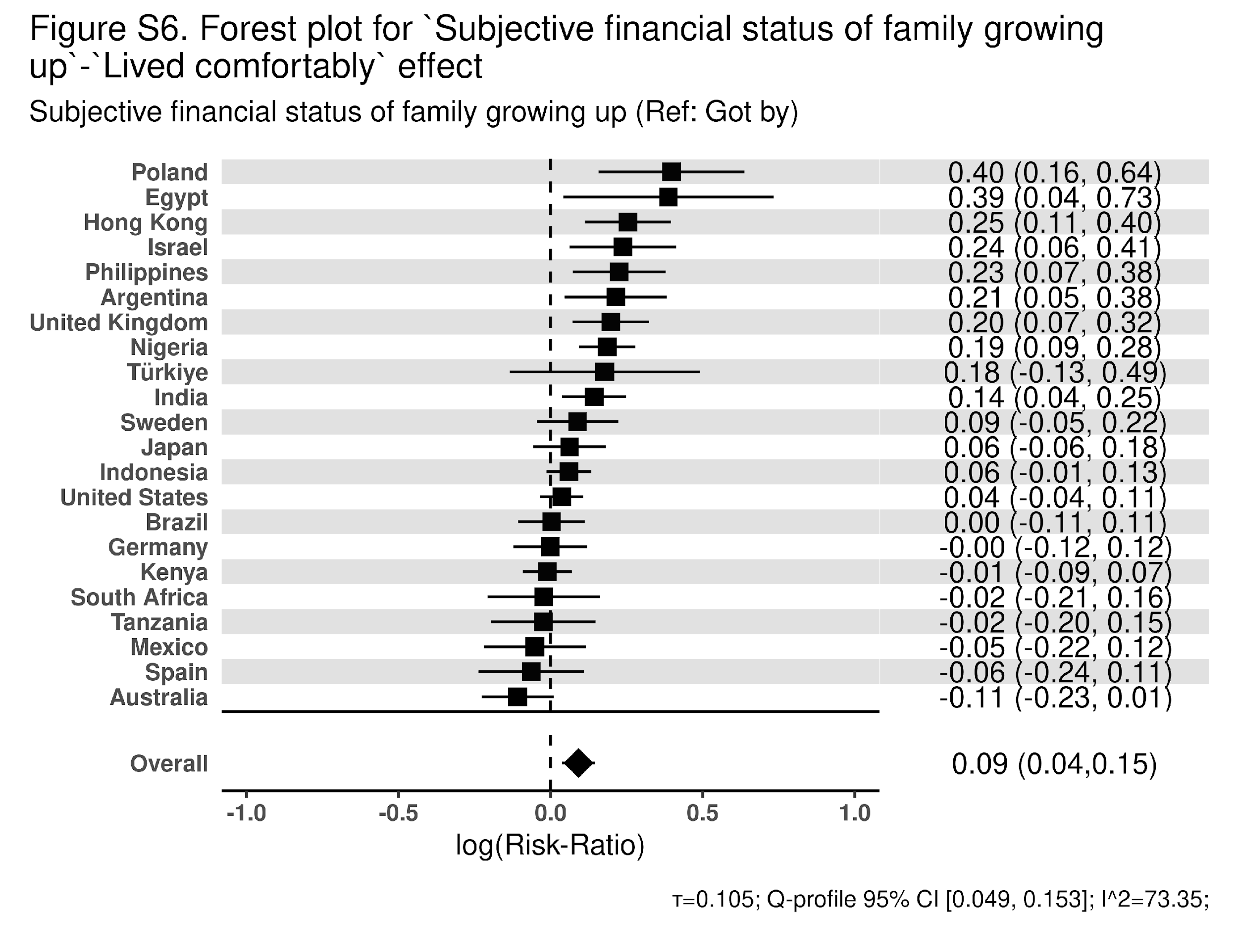

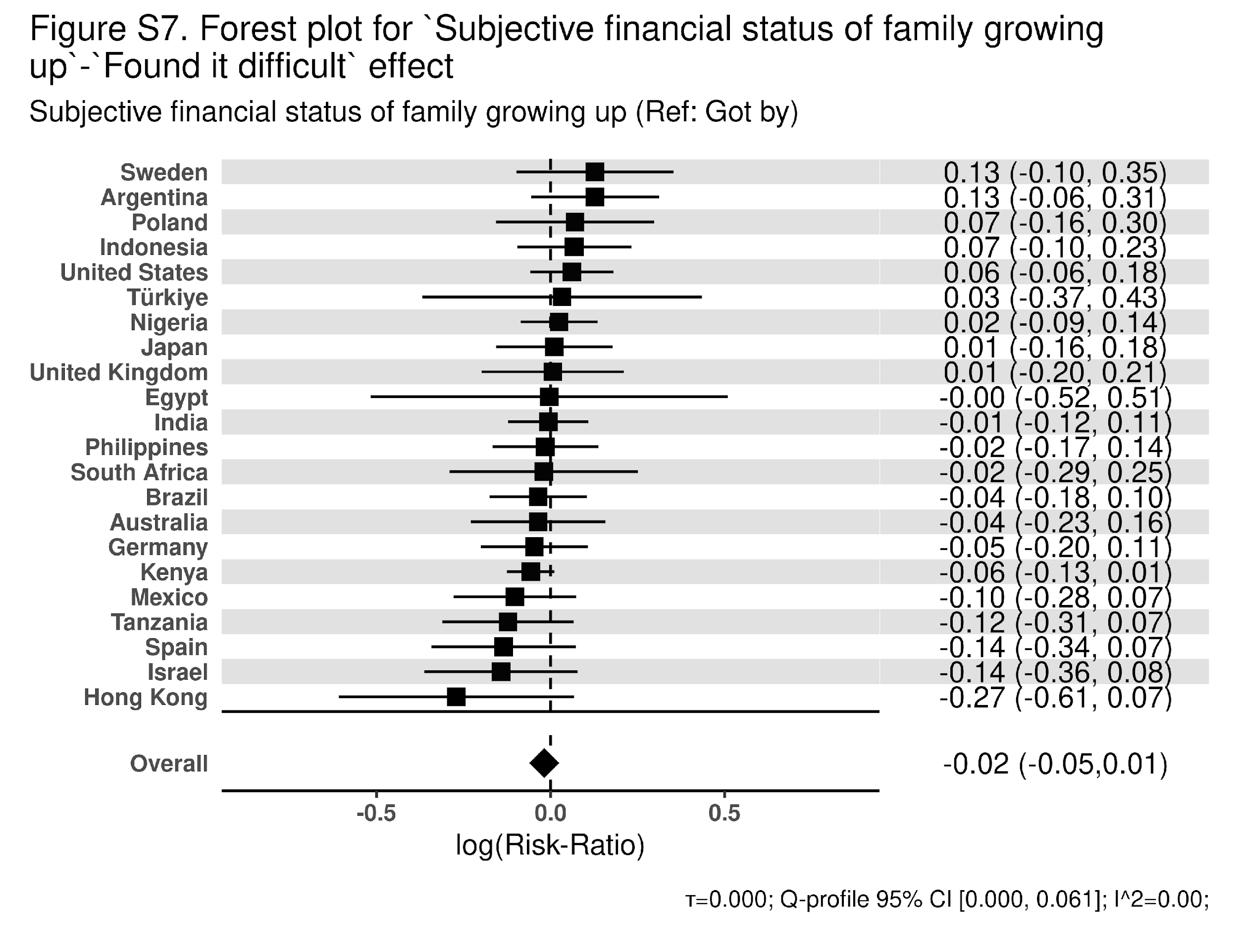

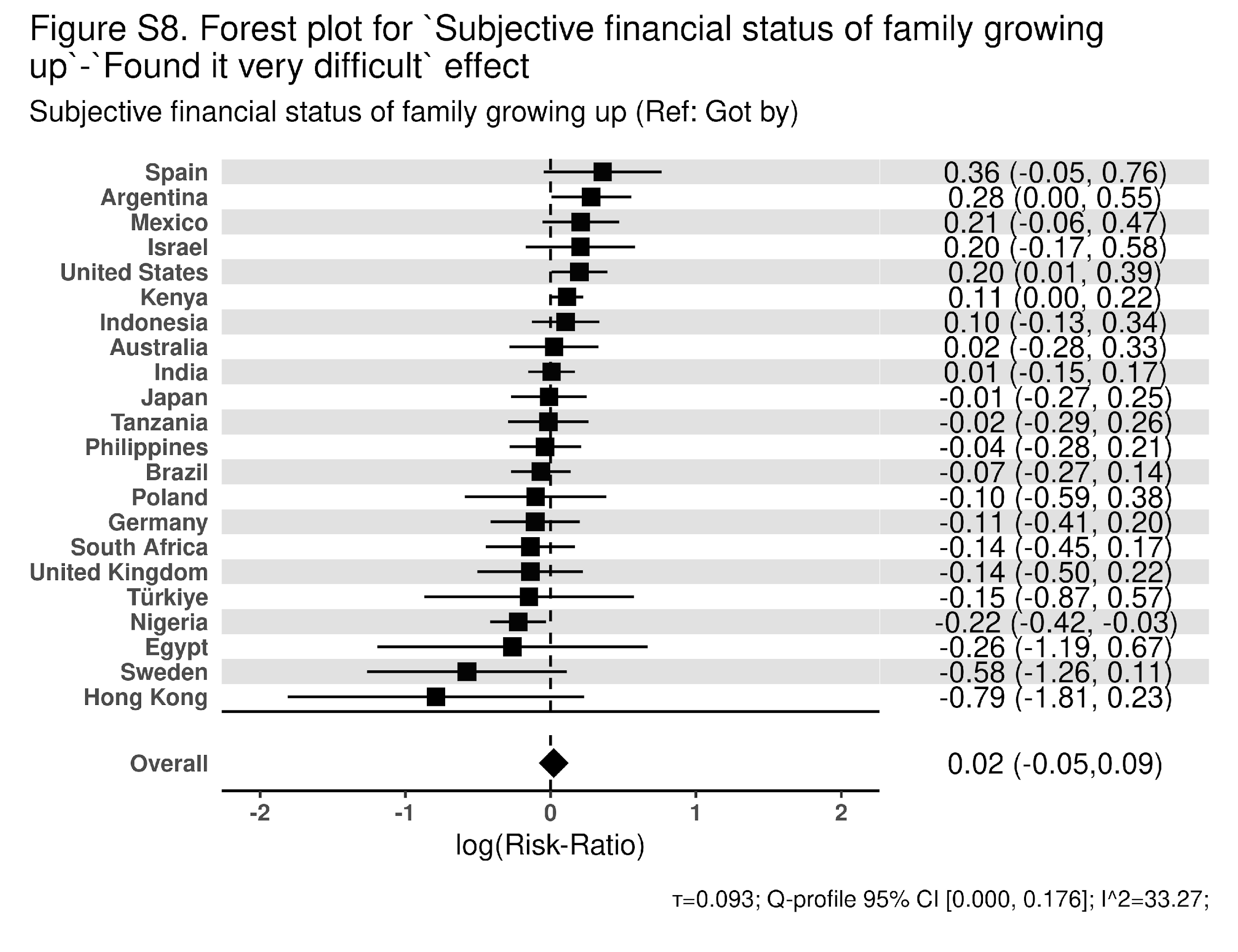

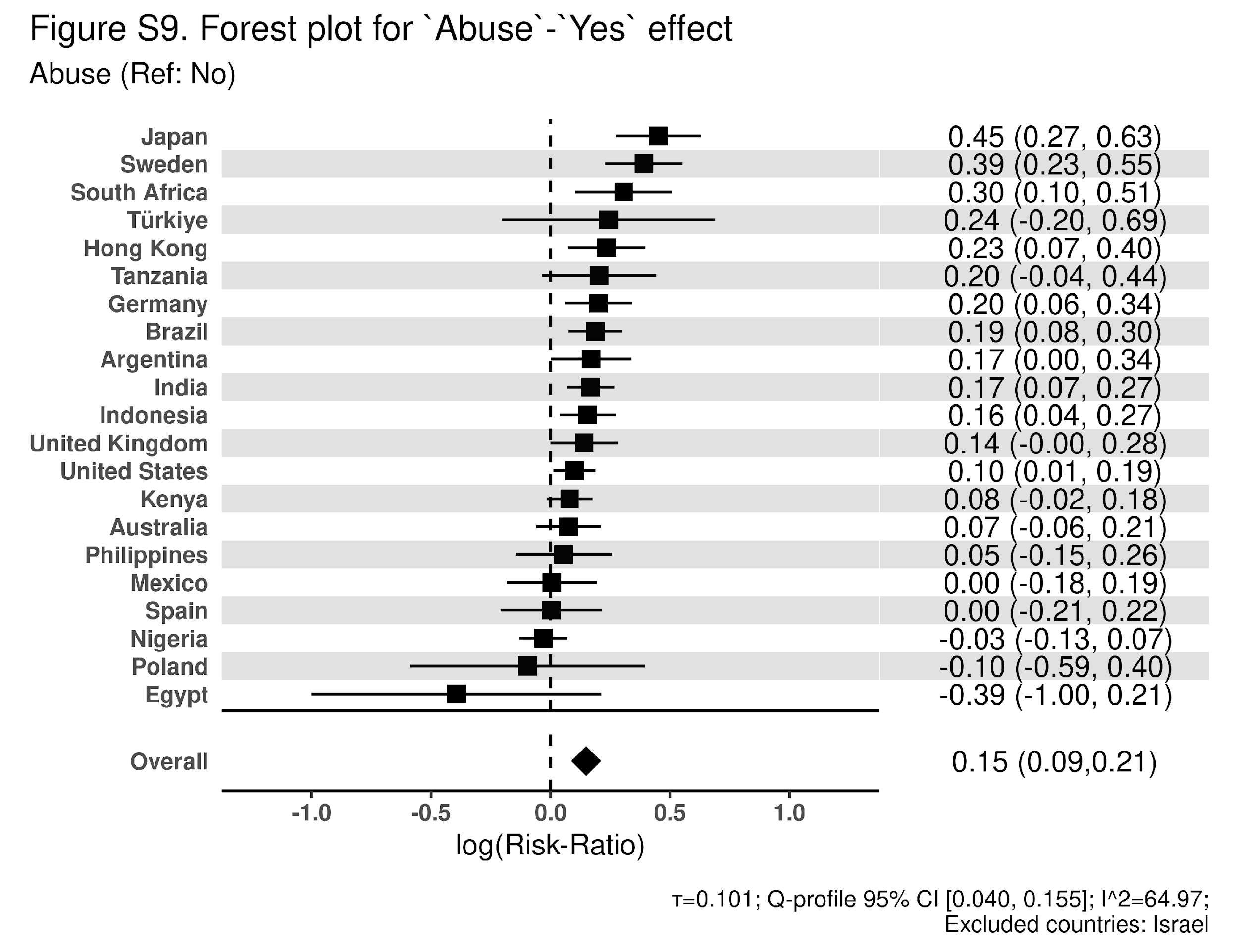

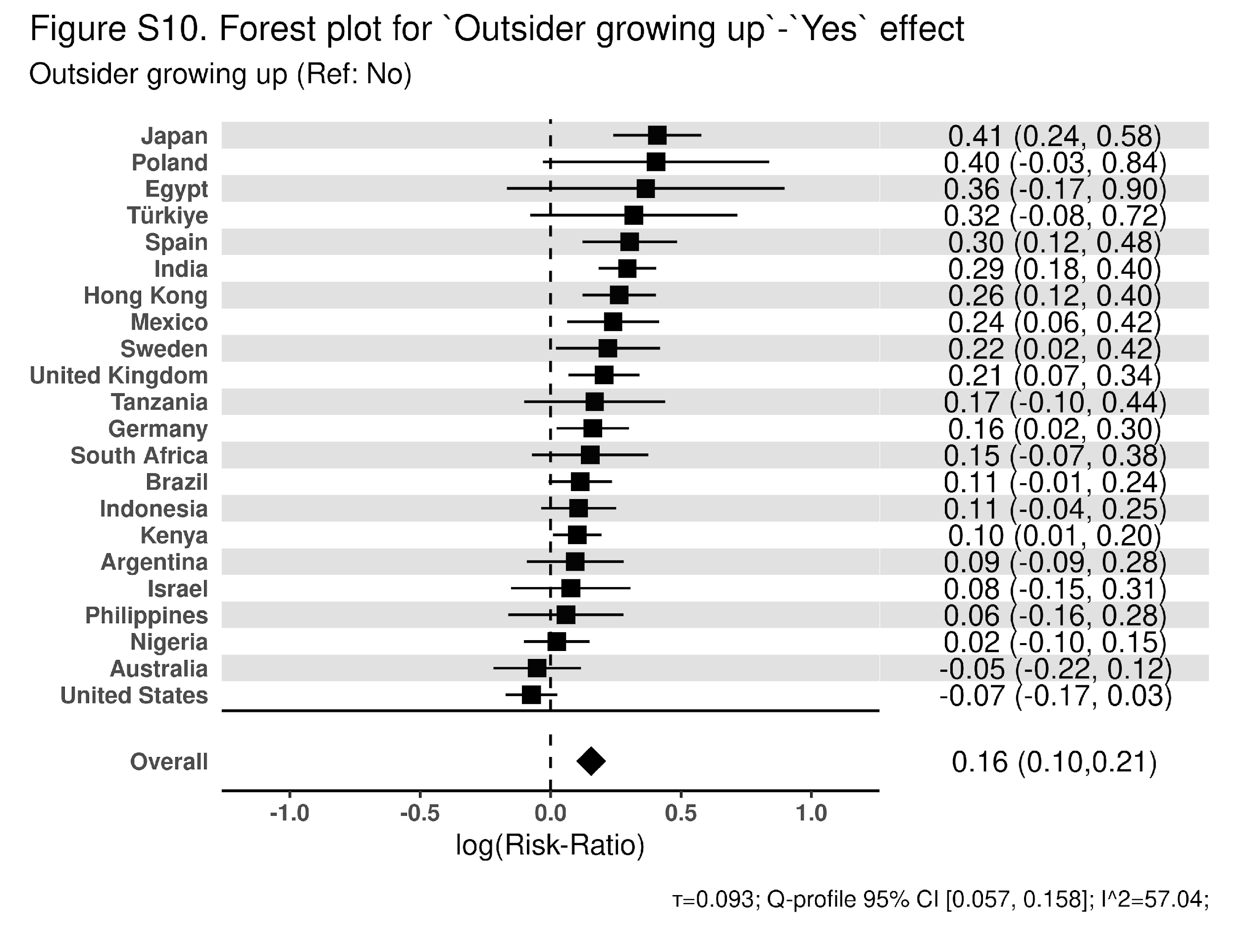

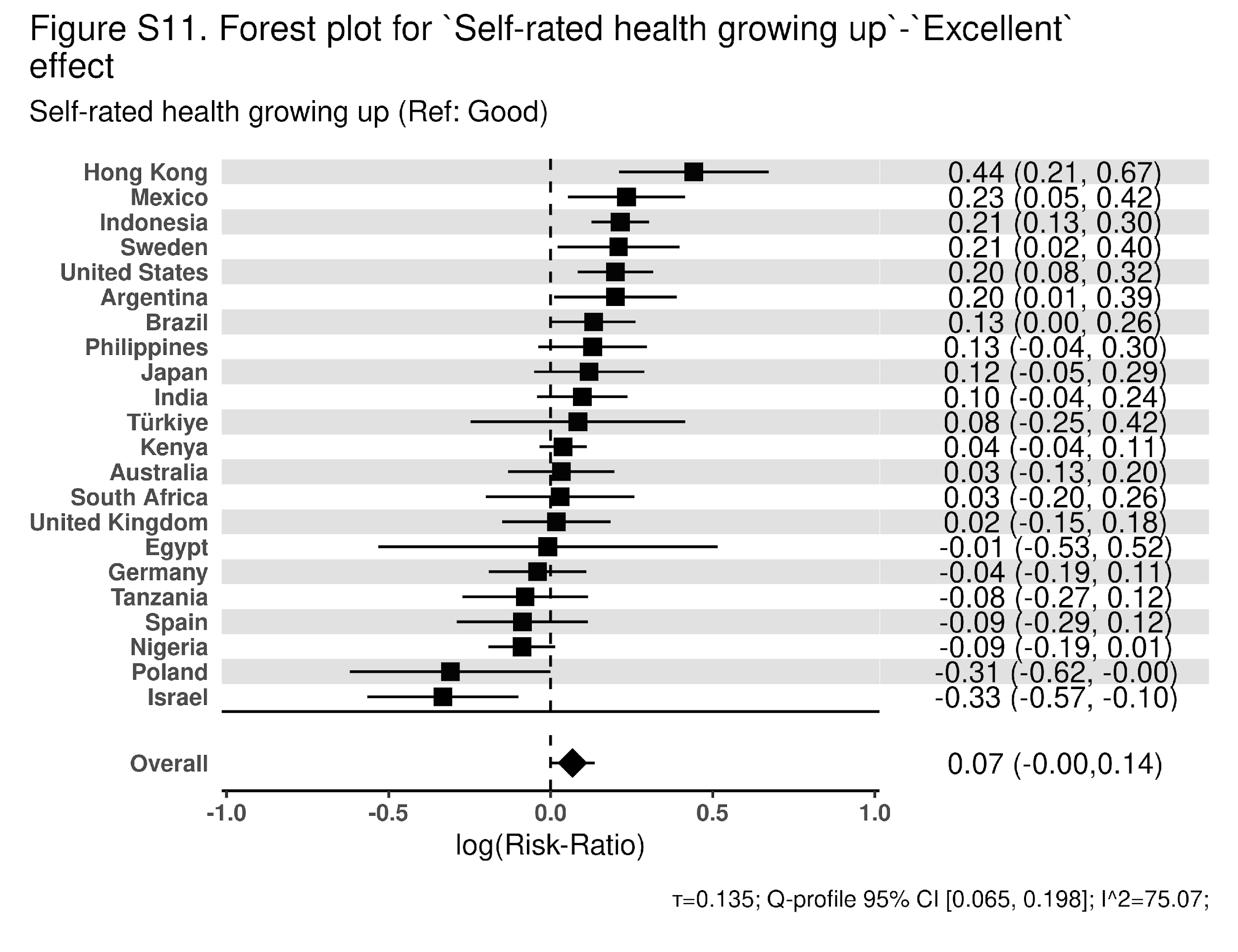

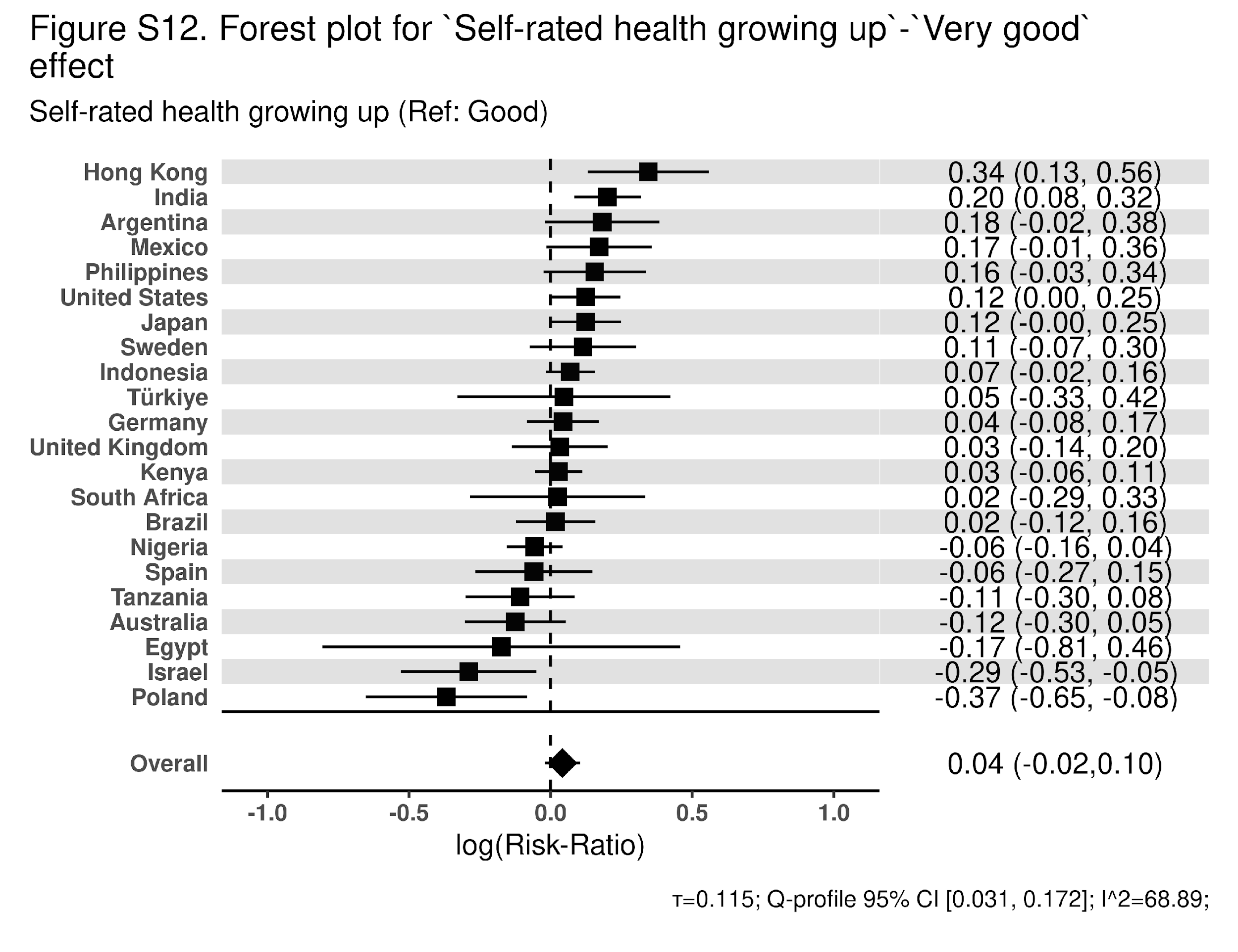

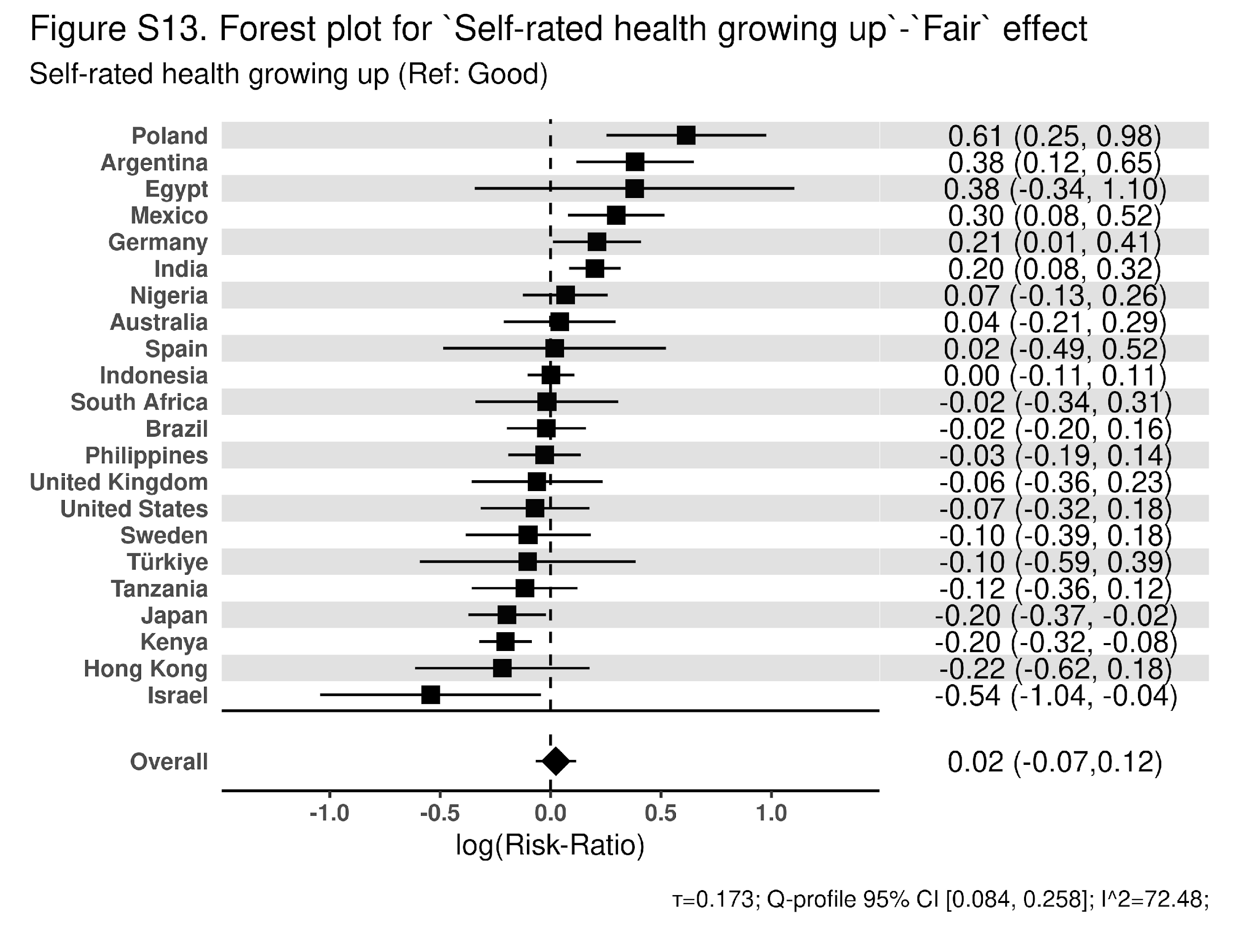

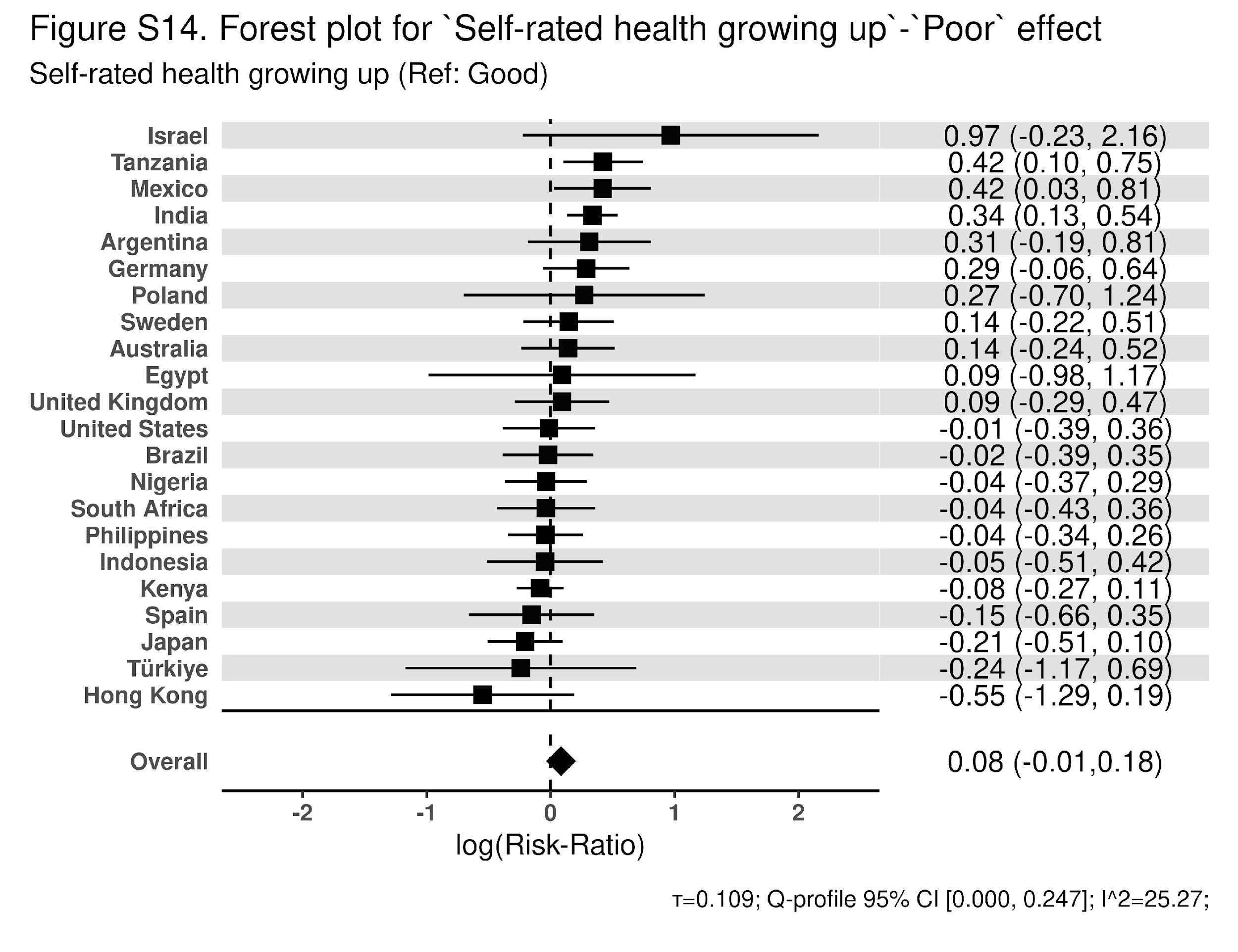

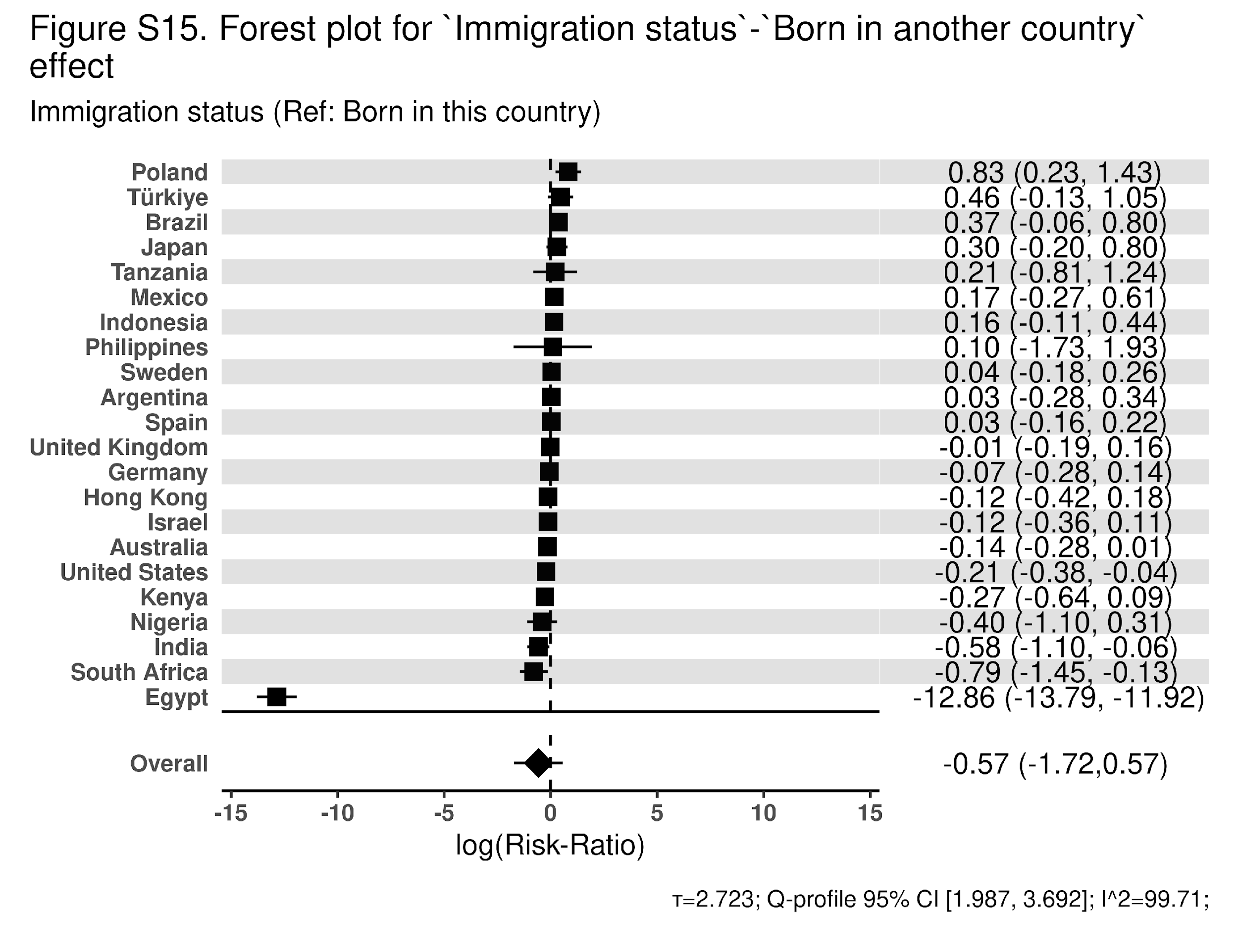

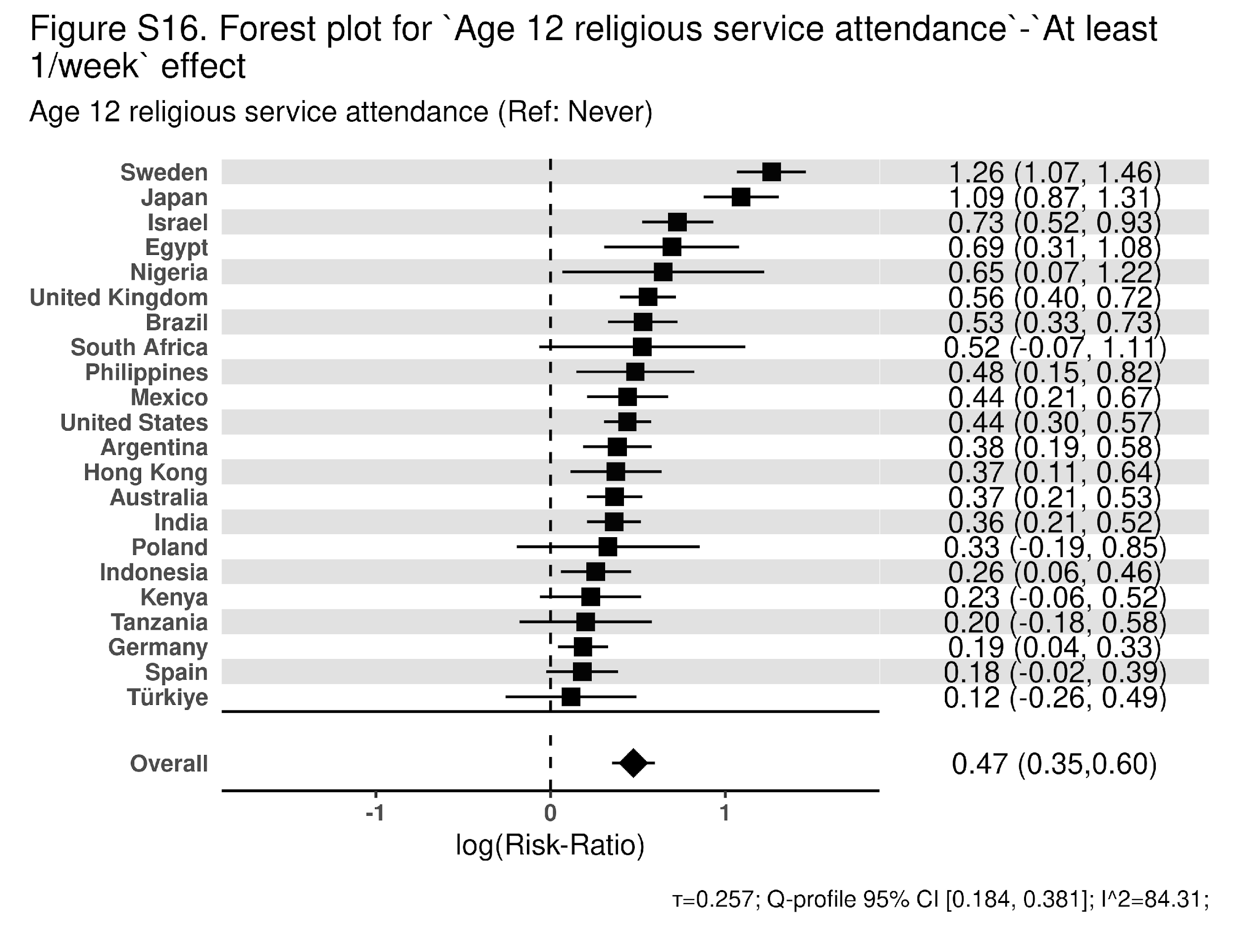

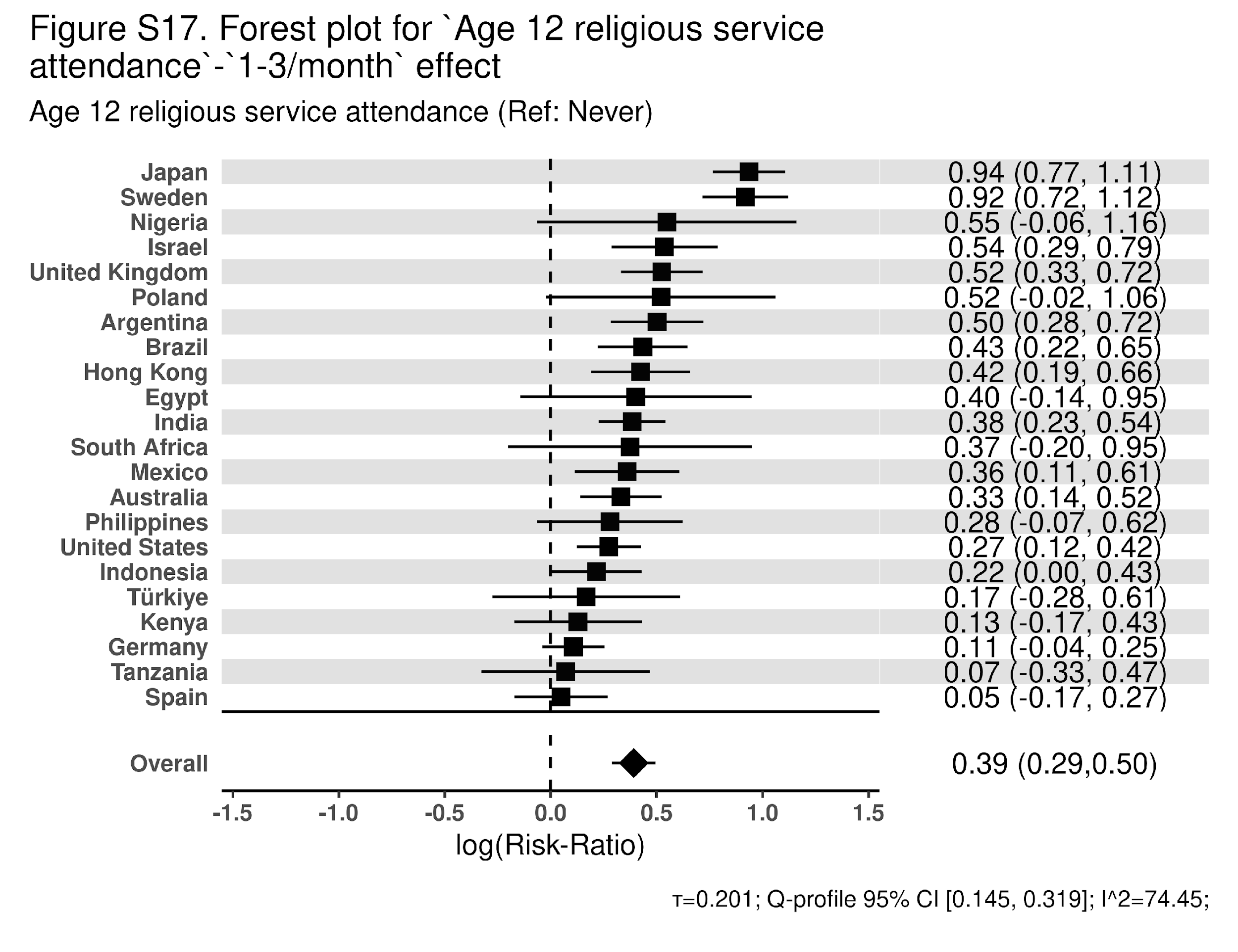

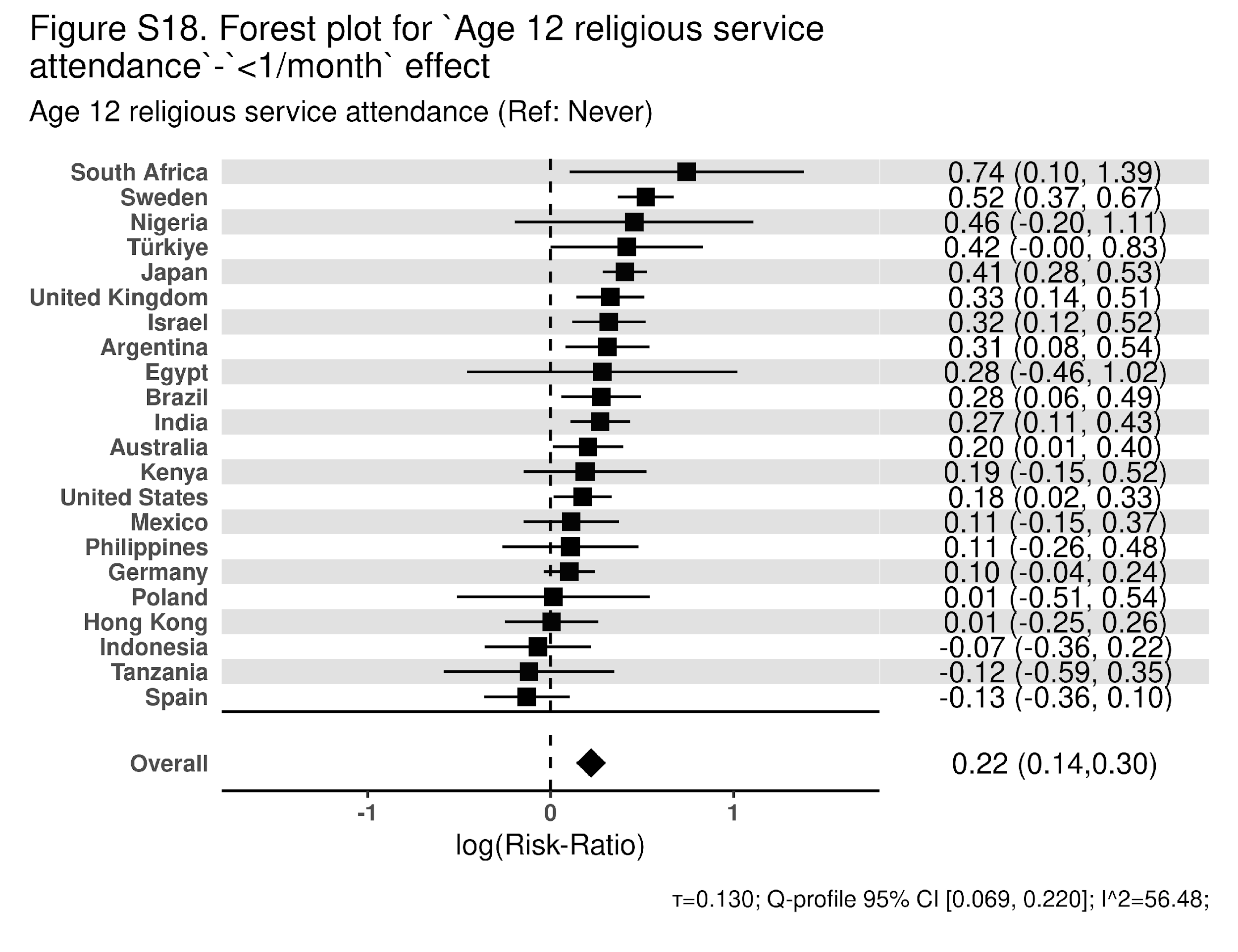

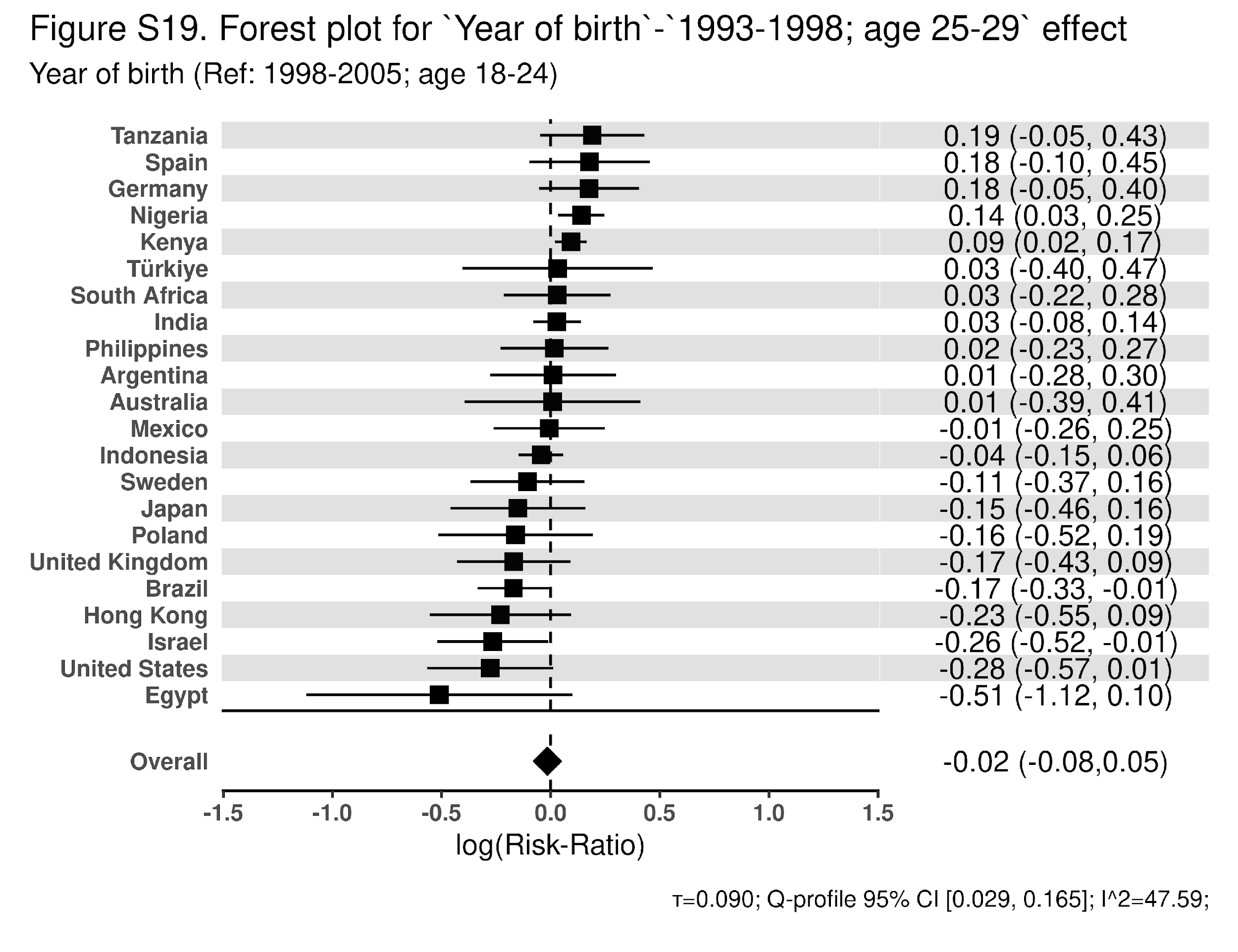

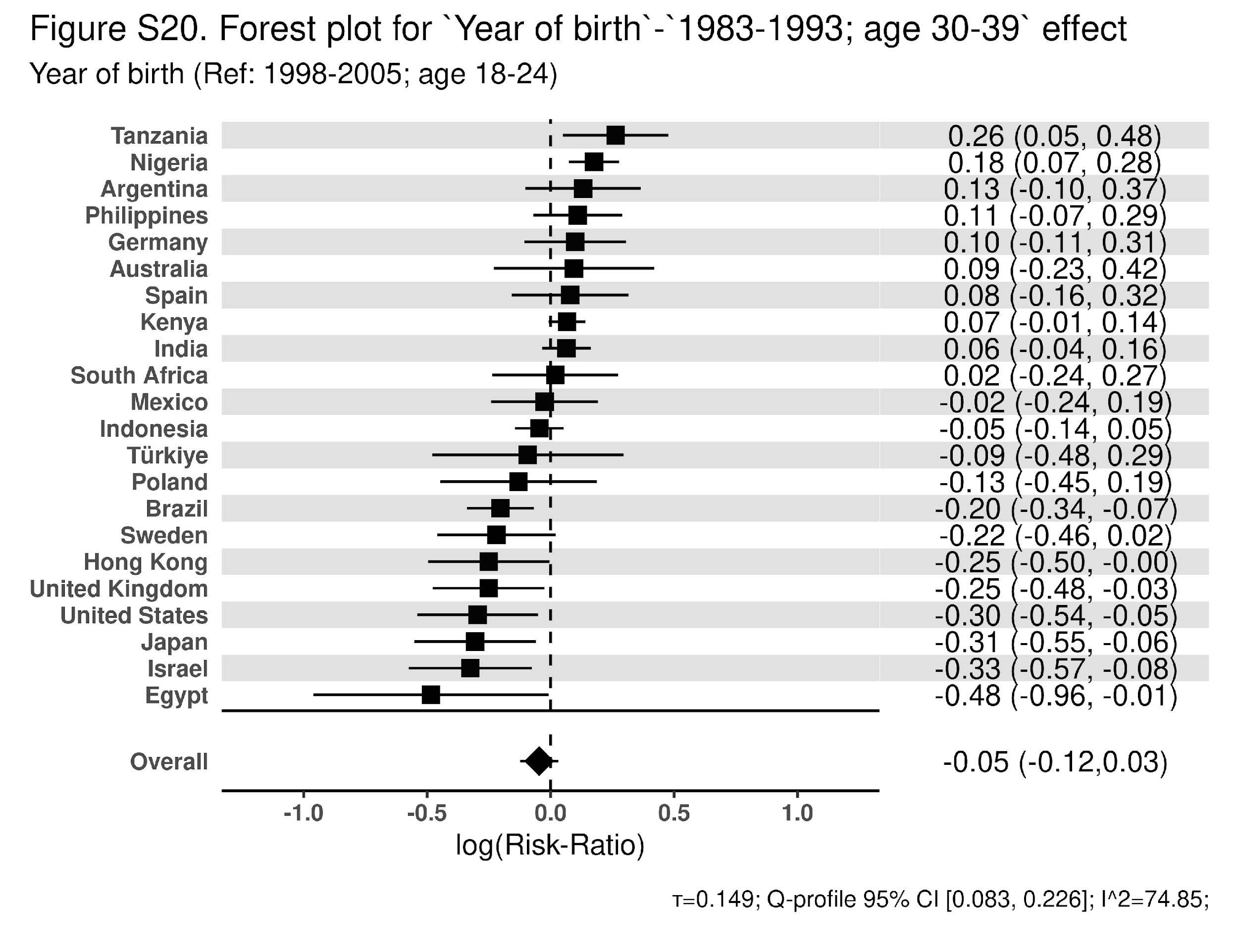

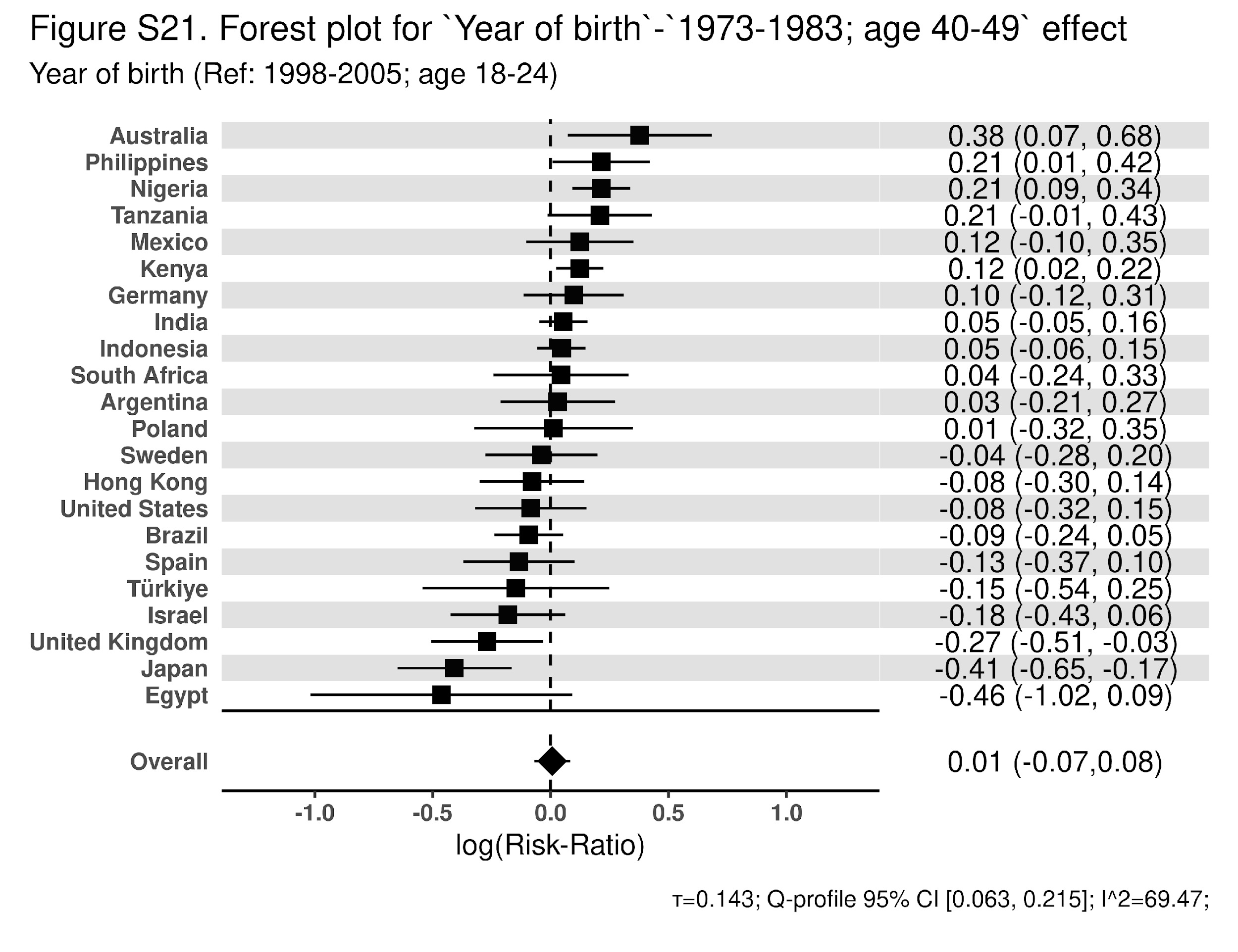

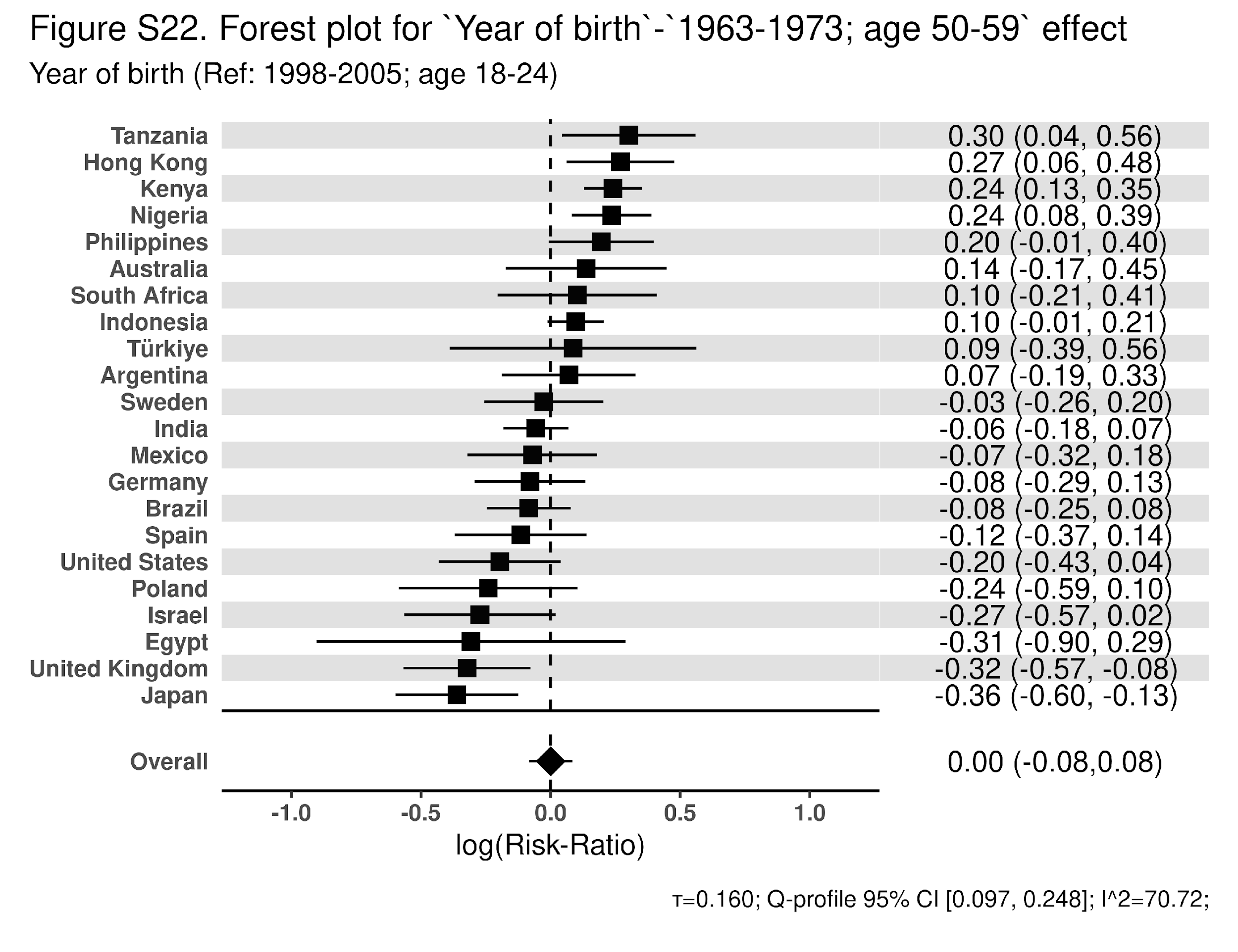

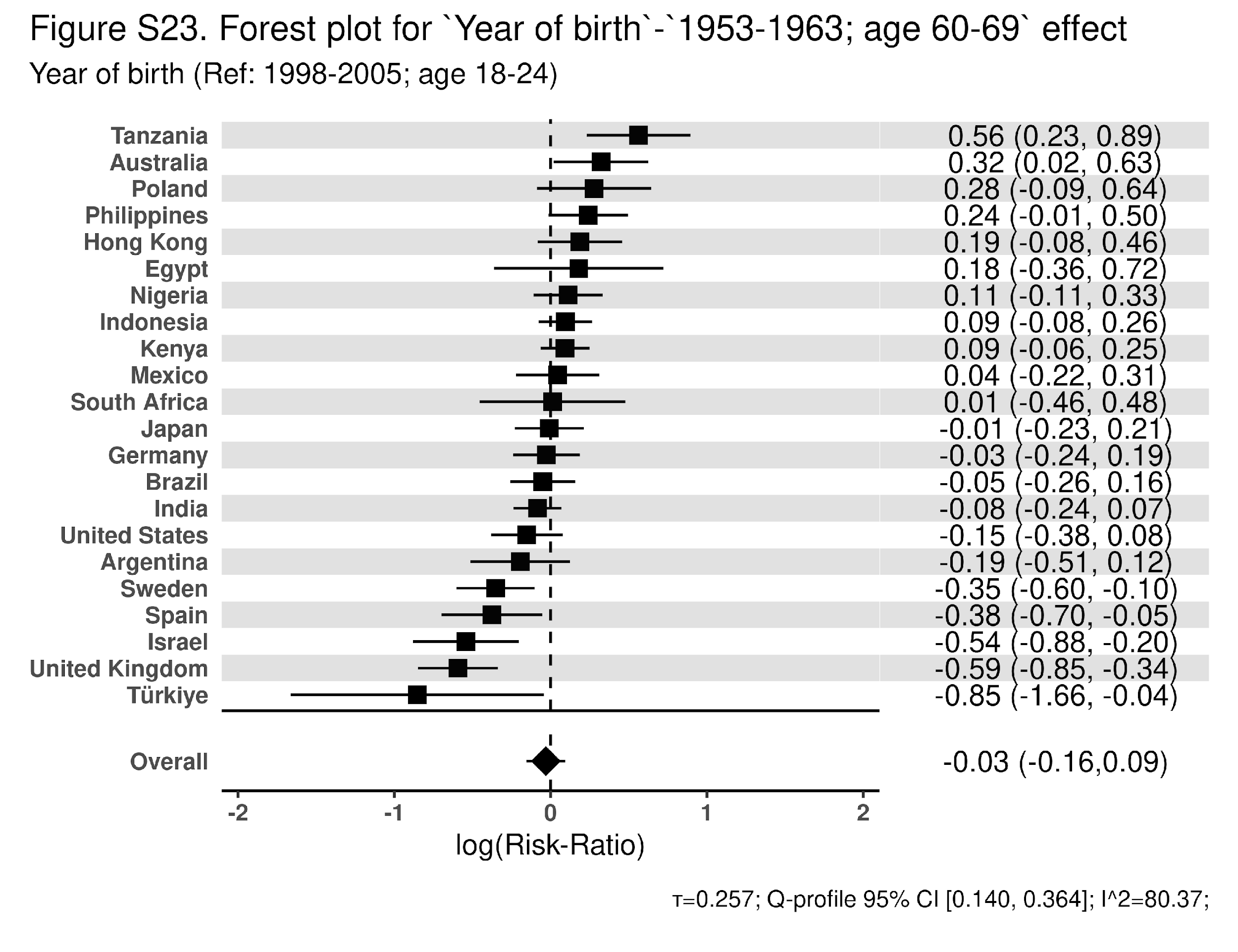

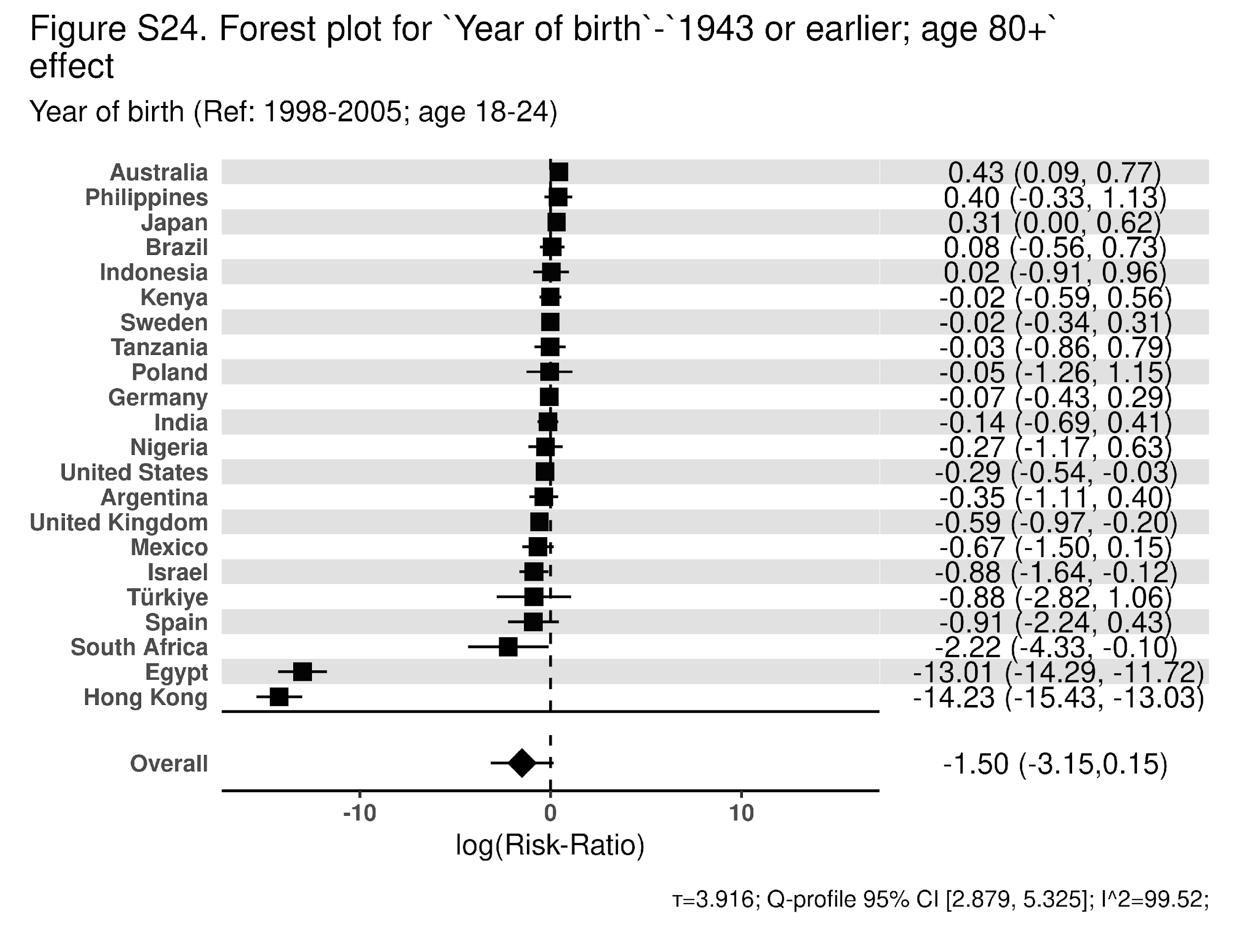

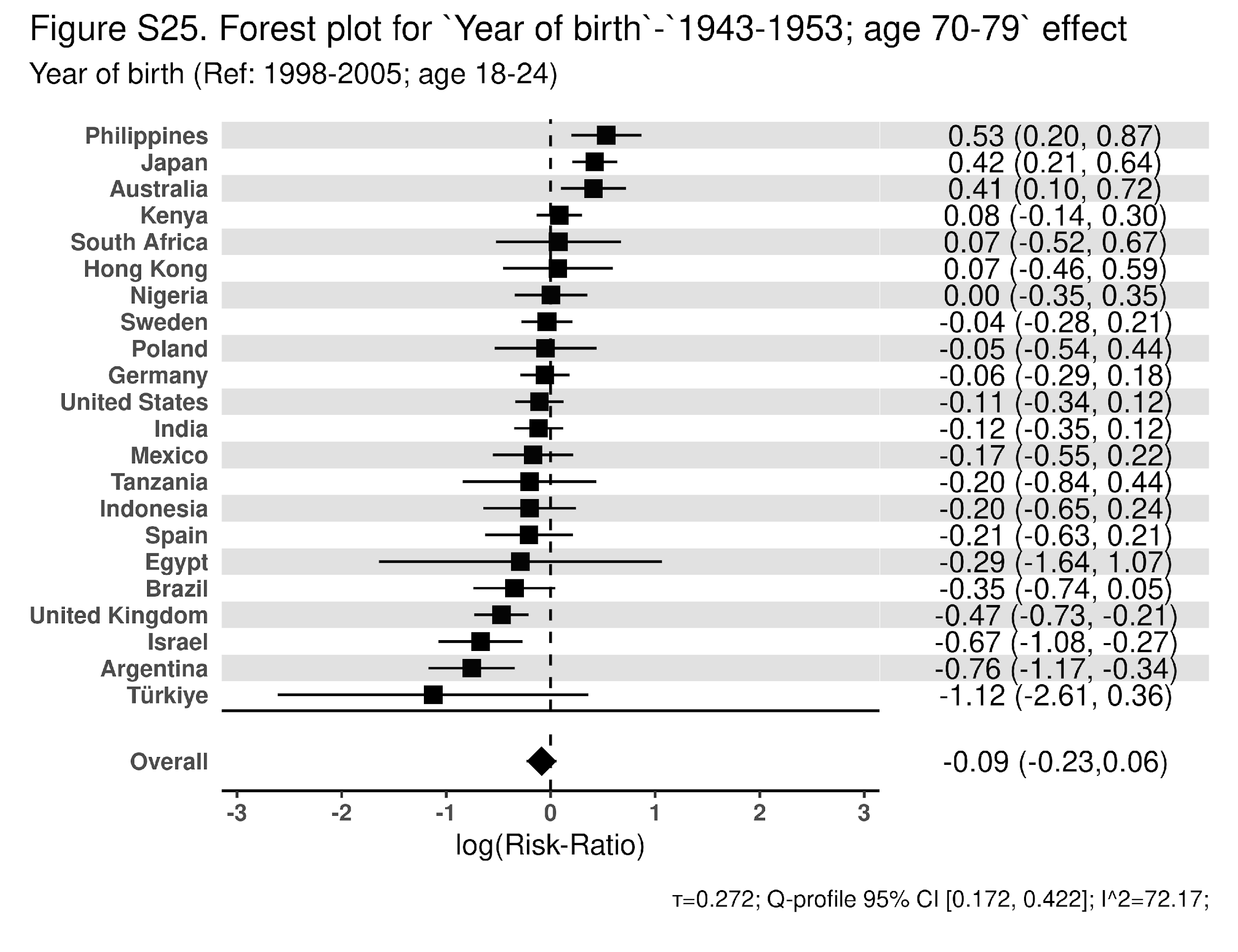

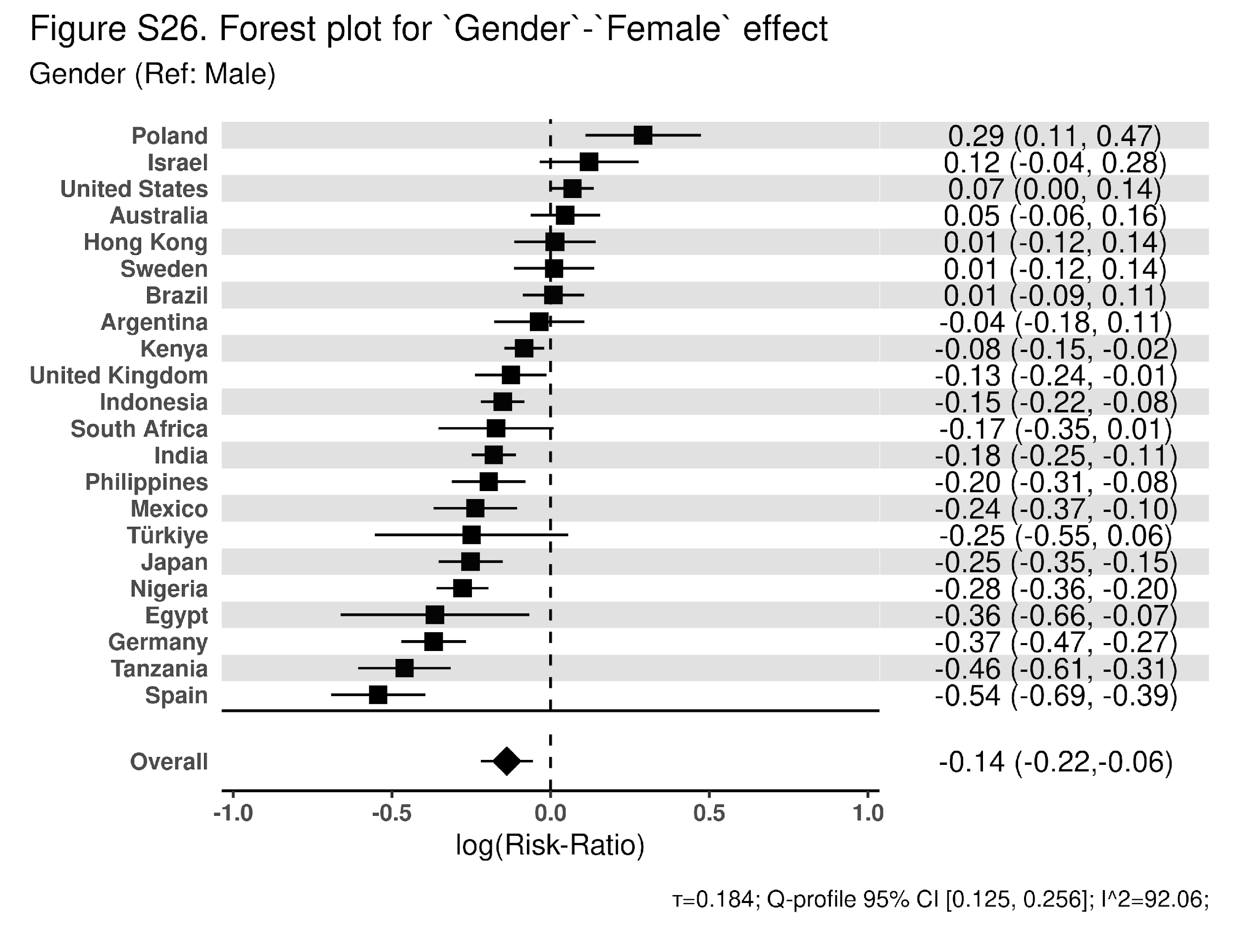

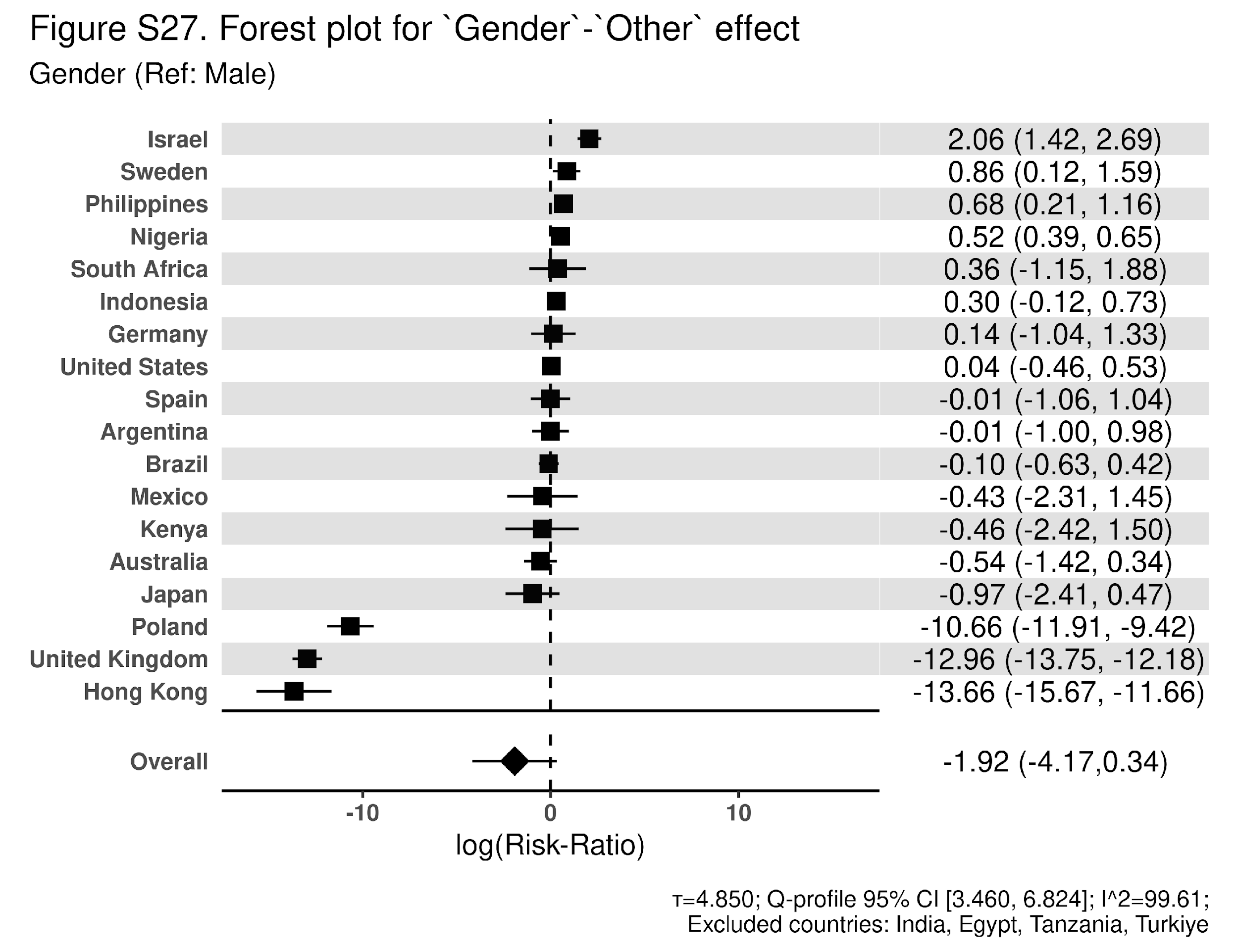

Supplement: Supplementary file 1 — Supplementary Material 1 [file 41598_2024_81639_MOESM1_ESM.docx]
